# Baicalin Relieves *Glaesserella parasuis*-Triggered Immunosuppression Through Polarization via MIF/CD74 Signaling Pathway in Piglets

**DOI:** 10.3390/biom15050640

**Published:** 2025-04-29

**Authors:** Shulin Fu, Qiaoli Dong, Yunjian Fu, Ronghui Luo, Jingyang Li, Yamin Sun, Siyu Liu, Yinsheng Qiu, Ling Guo, Jin Hu

**Affiliations:** 1 Wuhan Engineering and Technology Research Center of Animal Disease-Resistant Nutrition, School of Animal Science and Nutritional Engineering, Wuhan Polytechnic University, Wuhan 430023, China; 20230411045@whpu.edu.cn (Q.D.); 20230411033@whpu.edu.cn (Y.F.); 20230411035@whpu.edu.cn (R.L.); 20220411050@whpu.edu.cn (J.L.); 20230412014@whpu.edu.cn (Y.S.); 20220411030@whpu.edu.cn (S.L.); qiuyinsheng6405@whpu.edu.cn (Y.Q.); guoling1101@whpu.edu.cn (L.G.); hujin@whpu.edu.cn (J.H.); 2 Hubei Key Laboratory of Animal Nutrition and Feed Science, School of Animal Science and Nutritional Engineering, Wuhan Polytechnic University, Wuhan 430023, China

**Keywords:** baicalin, *Glaesserella parasuis*, inflammation, MIF/CD74, autophagy, polarization, immunosuppression

## Abstract

*Glaesserella parasuis* (*G. parasuis*) infection is responsible for Glässer’s disease in pigs. *G. parasuis* could trigger piglet immunosuppression, but the mechanism of inducing immunosuppression by *G. parasuis* remains unknown. Macrophage migration inhibitory factor (MIF)/CD74 axis has been shown to participate in inflammation response and immunosuppression, but the function of MIF/CD74 during immunosuppression elicited by *G. parasuis* has not been fully explored. This experiment explored the efficacy of baicalin on immunosuppression elicited by *G. parasuis* alleviation through regulating polarization via the MIF/CD74 signaling pathway. Our data indicated that baicalin reduced IL-1β, IL-6, IL-8, IL-18, TNF-α, and COX-2 expression, and regulated MIF/CD74 axis expression in the spleen. Immunohistochemistry analysis showed that baicalin enhanced CD74 protein levels in the spleen of piglets induced by *G. parasuis*. Baicalin regulated the PI3K/Akt/mTOR signaling pathway and RAF/MEK/ERK signaling activation, modified the expression of the autophagy-related proteins Beclin-1, P62, and LC3B, promoted M2 polarization to M1 polarization, and enhanced CD3, CD4, CD8, and TIM3 levels in the spleen of piglets elicited by *G. parasuis*. Our study reveals the important functions of the MIF/CD74 axis in *G. parasuis*-induced immunosuppression and may offer a new therapeutic method to control *G. parasuis* infection.

## 1. Introduction

*Glaesserella parasuis* (*G. parasuis*) is a significant pathogen responsible for Glässer’s disease in pigs [1]. The symptoms caused by *G. parasuis* are fibrotic polyserositis, arthritis, and meningitis [2]. So far, 15 serovars have been verified by using gel immunodiffusion determination; however, more than 20% of isolates have not been serotyped using this method [3]. Serovars 4, 5, and 13 were considered as the highly virulent strains [4]. *G. parasuis* is a bacterium that colonizes the upper respiratory tract of pigs, which can spread rapidly within a herd [5]. Once inside the host, *G. parasuis* has the potential to invade and multiply within the tissues, leading to an acute systemic inflammatory response [6]. This infectious condition poses a considerable threat to swine production worldwide, contributing to substantial economic losses to the pig farms [7]. Prevention and control measures are crucial to mitigate the harmful effects of *G. parasuis*. However, the diversity of *G. parasuis* serovars lacks cross-serovar protection, contributing to limiting the effectiveness of vaccines [8]. Thus, it is urgent to find new drugs to prevent and control *G. parasuis* infection.

Infection of piglets with *G. parasuis* can result in immunosuppression in piglets [9]; however, the mechanism of immunosuppression elicited by *G. parasuis* in piglets is still unknown. Leukocyte differentiation antigen 74 (CD74), the MHC II-associated invariant chain, is a type II transmembrane glycoprotein [10]. CD74 promoted the formation of the tumor microenvironment through enhancing S100A8 and S100A9 production in the pancreatic cancer [11]. CD74 aided the accumulation and efficacy of regulatory T cells in tumors [12] and was involved in *Staphylococcus aureus*-induced dose-dependent immunosuppression [13]. In addition, CD74 was considered the receptor for the macrophage migration inhibitory factor (MIF) [14]. As a receptor for CD74, MIF can regulate the innate and adaptive immune response [15]. MIF has been shown to play a key role in immune system regulation and is involved in autoimmune disorders and tumorigenesis [16]. Furthermore, some studies indicate that MIF contributes to human melanoma myeloid-derived suppressor cell induction and T-cell immunosuppression [17]. Research has reported that MIF/CD74 damaged LECs’ barrier via inhibiting tight junction proteins in an ERK1/2-dependent manner [18]. Interaction between CD74 and MIF enhances the secretion of proinflammatory mediators and promotes unactivated CD4^+^ T cell infection, leading to viral spreading [19]. Blockade with immunosuppressive MIF/CD74 on macrophages and dendritic cells contributed to the downregulation of immunosuppressive factors from MOs and the upregulation of the capacity of DCs to activate cytotoxic T cells [20]. However, whether MIF/CD74 signaling was related to immunosuppression in piglets induced by *G. parasuis* has not been evaluated.

Bacterial antibiotic resistance is becoming more widespread and increasingly serious due to excessive utilization of antibiotics in healthcare and agriculture [21]. A Chinese veterinary drug was thought to be a good antibiotic alternative to relieve the negative impacts of infectious diseases [22]. Levamisole, a compound with established immunomodulatory properties, has been used as an immunostimulant and anti-cancer drug in humans and farm animals, making it an effective control in this study [23]. Baicalin, a flavonoid compound, is extracted from the root of *Scutellaria baicalensis* Georgi [24]. Baicalin possesses anti-inflammatory, antibacterial, antioxidant, and immunomodulatory properties [25]. It has been reported that baicalin suppressed macrophage JNK/SIRT1/p65-triggered adipose tissue inflammation [26] and diminished hepatocyte injury resulting from aflatoxin B_1_ through TP53-related ferroptosis signaling [27]. In addition, baicalin reduced inflammatory responses to reduce lung cancer by acting on SOCS1/NF-κB/STAT3 axis [28], ameliorated *Mycoplasma gallisepticum*-elicited inflammatory damage by diminishing ceramide accumulation in DF-1 cells [29] and alleviated lung inflammatory responses and fibrosis elicited by silica through diminishing TLR4/NF-kB pathway in rats [30]. Baicalin also weakened porcine extraintestinal pathogenic *Escherichia coli* virulence through reducing the LuxS/AI-2 quorum-sensing system [31], and attenuated intestinal inflammatory damage triggered by LPS through diminishing NF-κB and NLRP3 pathways [32]. Baicalin inhibited PANX-1/P2Y6 signaling pathway activation in porcine aortic vascular endothelial cells infected by *G. parasuis* [33]. Although baicalin has important biological functions, the mechanism by which baicalin can alleviate *G. parasuis* elicited piglet immunosuppression via MIF/CD74 signaling has not been thoroughly studied.

In this study, the molecular mechanism of MIF/CD74-induced host immunosuppression via polarization in piglet spleen triggered by *G. parasuis* and whether baicalin could alleviate immunosuppression was investigated. This study deepens our knowledge about the molecular mechanism of inducing immunosuppression and provides new targets to prevent *G. parasuis* infection.

## 2. Materials and Methods

### 2.1. Ethics Statement

The animal study was approved by the Animal Care and Use Committee of Wuhan Polytechnic University, Hubei Province, China (WPU202308003), 2 August 2023. All experimental animals were euthanised at the end of the experiment.

### 2.2. Bacteria and Drugs

The *G. parasuis* SH0165 strain, serovar 5, used in this study, is highly virulent. It was isolated from the lung of a commercial pig which had the symptoms of arthritis, fibrinous polyserositis, hemorrhagic pneumonia, and meningitis [34]. The *G. parasuis* bacteria were grown on TSA or cultured in TSB at 37 °C. Simultaneously, 10 μg/mL of NAD (Guangzhou Saiguo Biotech Co., Ltd., Guangzhou, China) and 10% foetal bovine serum (Sijiqing, Hangzhou, China) were added to the culture.

Baicalin was obtained from Sichuan Xieli Pharmaceutical Co., Ltd. (Pengzhou, Sichuan, China). Levamisole was obtained from Beijing Solarbio Science & Technology Co., Ltd. (Beijing, China).

### 2.3. Design of the Experiment

The spleen tissue of piglets was obtained from our previous animal experiment [35]. Briefly, sixty 30-day-old naturally farrowed early-weaned piglets were randomly divided into the control group, the infection group, the levamisole group, the 25 mg/kg baicalin group, the 50 mg/kg baicalin group, and the 100 mg/kg baicalin group. All piglets were pretreated with an intramuscular injection of 15 mg/kg body weight (BW) levamisole, 25 mg/kg BW baicalin, 50 mg/kg BW baicalin, or 100 mg/kg BW baicalin, except the control group and the infection group. A total of 2 h later, after the first day of administration, all piglets were intraperitoneally infected with 1 × 10^8^ CFU *G. parasuis* in 1 mL of TSB except the control group. The piglets from the control group were intraperitoneally injected with an equivalent volume of TSB. After being challenged by *G. parasuis* for 6 h, the groups were injected with the same drugs, except the control group. Every treatment was administered twice a day for 2 days. All groups were observed for 7 days after *G. parasuis* infection, and the spleens were collected for analysis.

### 2.4. RT-PCR

The spleens were collected from all groups for cytokine IL-1β, IL-6, IL-8, IL-10, IL-18, TNF-α, and COX-2 production measurement by RT-PCR [36]. Briefly, the RNAs from the spleens were extracted by using the TRIzol reagent (Invitrogen, Carlsbad, CA, USA). After analysis of the concentration and quality using a Qubit 2.0 fluorometer (Thermo Fisher Scientific, Waltham, MA, USA), the RNA was reverse transcribed to complementary DNA by using a reverse transcriptase (Abclonal, Wuhan, China) and PCR determination was implemented by employing a SYBR Green PCR Kit (Abclonal, Wuhan, China). The transcriptions of each sample were repeated at least three times. GAPDH was employed as the internal control. The relative expression levels of genes were quantified employing the threshold cycle (CT) method. The gene relative expression fold changes were performed employing the 2^−ΔΔCT^ CT formula. The RT-PCR primers used in this study are listed in Table 1.

### 2.5. Determination of Baicalin on CD3 Expression by Immunofluorescence

Immunofluorescence analysis was employed to assess the function of baicalin and levamisole on the CD3 level in the spleen tissue [37]. Briefly, the spleen tissues were fixed with paraformaldehyde and then treated with 0.5% Triton X-100 (Sangon Biotech, Shanghai, China). Following blocking with 5% BSA at 37 °C for 30 min, the samples were incubated with a primary antibody against CD3 (SouthernBiotech, Birmingham, AL, USA) for 12 h at 4 °C. The nuclei were counterstained with DAPI (Servicebio, Wuhan, China). Afterwards, the tissues were labeled and washed, the fluorescent photographs were obtained using a Nikon Eclipse C1 microscope (Nikon, Tokyo, Japan). Fluorescence intensity and cell surface area were measured by utilizing the Case Viewer software (CaseViewer 3.3, 3DHISTECH, Budapest, Hungary).

### 2.6. Detection of CD74 Expression by Immunohistochemistry

The CD74 expression level was also determined by using immunohistochemistry analysis [38]. Briefly, spleen tissues were prepared from paraffin-embedded blocks. Then, slices were cut and incubated with an anti-CD74 antibody (Abclonal, Wuhan, China, 1:500, Cat: A24027) for immunohistochemistry (IHC) at 4 °C overnight. CD74 was reacted with goat anti-mouse antibody (1:200) and visualized using diaminobenzidine (DAB) (Servicebio, Wuhan, China). The negative control was only added with the second antibody. Following counterstaining with hematoxylin (Servicebio, Wuhan, China) for 3 min, the microscopic observation was carried out, and the Image 1.52a software was employed to determine the optical density sum of the brown area (Olympus BX43, Tokyo, Japan).

### 2.7. Western Blotting

The protein levels were estimated by the Western blotting method [39]. Briefly, the tissue proteins were extracted by employing the RIPA Lysis Buffer (Beyotime Biotechnology, Shanghai, China). The protein concentration was determined by employing an Enhanced BCA Protein Assay Kit (Beyotime Biotechnology, Shanghai, China), and then isolated by employing 10% SDS-PAGE and transferred onto a PVDF (polyvinylidene difluoride) membrane. Flowing being blocked by using BSA for 120 min and washed five times using TBST, the membrane was incubated with the primary antibody of MIF (Boster biological technology, Wuhan, China, 1:2000, Cat: BA2058), CD74 (ABclonal, 1:10,000, Cat: A24027), PI3K (ABclonal,1:500, Cat: A4992), p-PI3K (ABclonal, 1:1000, Cat: AP0427), Akt (proteintech, 1:2000, Cat: 10176-2), p-Akt (proteintech, 1:2000, Cat: 66444-1), mTOR (proteintech, 1:5000, Cat: 66888-1), p-mTOR (proteintech, 1:20,000, Cat: 67778-1), RAF (Abmart, Shanghai, China, 1:2000, Cat: T55225), MEK1/2 (ABclonal, 1:6000, Cat: A4868), p-MEK1/2 (ABclonal, 1:10,000, Cat: AP1349), ERK1/2 (ABclonal, 1:5000, Cat: A22447), p-ERK1/2 (ABclonal, 1:2000, Cat: AP0485), P62 (ABclonal, 1:40,000, Cat: A19700), LC3B (ABclonal, 1:4000, Cat: A19665), Beclin-1 (ABclonal, 1:10,000, Cat: A21191), or GAPDH (proteintech, 1:5000, Cat:10494-1-AP) for 12 h at 4 °C, respectively. The membrane was washed with TBST five times and incubated with corresponding HRP Goat Anti-Rabbit IgG (MIF, CD74, PI3K, p-PI3K, Akt, RAF, MEK1/2, p-MEK1/2, ERK1/2, p-ERK1/2, P62, LC3B, Beclin-1, GAPDH) (Abbkine, Wuhan, China, 1:10,000) or HRP Goat Anti-Mouse IgG (H+L) (p-Akt, mTOR, p-mTOR) (ABclonal, 1:5000) at 37 °C for 1 h, and were visualized with an ECL Enhanced Kit (ABclonal, Wuhan, China). The protein levels were quantified in a FluorChem™ FC2 AIC system (Alpha Innotech, Santa Clara, CA, USA) by employing ImageJ software (V1.8.0.112).

### 2.8. Statistical Analysis

The results are presented as the mean ± standard deviation. Statistical differences were carried out with one-way analysis of variance (ANOVA). ANOVA was used when three or more independent groups were compared. *p* < 0.05 was considered significantly different. ^##^ *p* < 0.01 vs. controls; ^###^ *p* < 0.001 vs. controls; * stands for *p* < 0.05; ** stands for *p* < 0.01; *** stands for *p* < 0.001.

## 3. Results

### 3.1. Baicalin Inhibited IL-1β, IL-6, IL-8, IL-18, TNF-α, and COX-2 Expression in Piglet Spleen Triggered by G. parasuis

**Figure 1:**
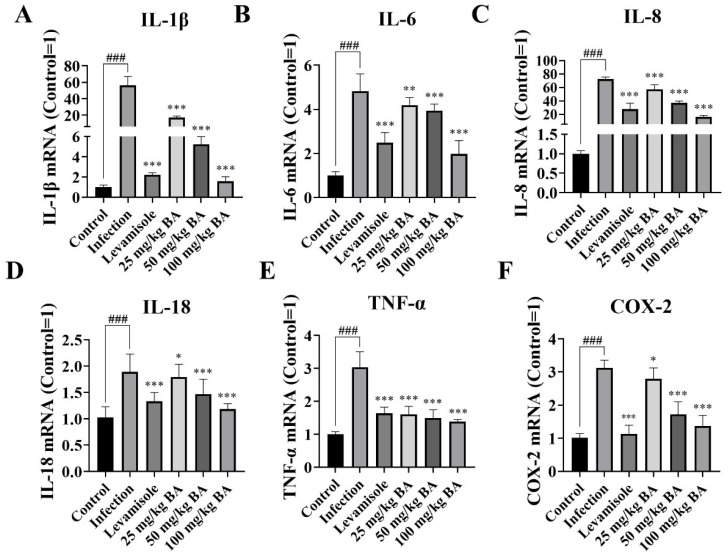
The effect of baicalin on cytokine production in piglet spleen triggered by *G. parasuis*. The IL-1β (**A**), IL-6 (**B**), IL-8 (**C**), IL-18 (**D**), TNF-α (**E**), and COX-2 (**F**) mRNA levels were determined by RT-PCR. BA: Baicalin; ^###^ *p* < 0.001 vs. controls; * stands for *p* < 0.05; ** stands for *p* < 0.01; *** stands for *p* < 0.001.

Cytokines are multifunctional mediators of cellular communication that are involved in inflammatory responses [40]; thus, we analyzed the effect of baicalin on cytokine production in piglets infected with *G. parasuis*. The data indicated that IL-1β, IL-6, IL-8, IL-18, TNF-α, and COX-2 mRNA levels in the spleen in the infection group were significantly upregulated (*p* < 0.001) (Figure 1). Levamisole significantly inhibited IL-1β, IL-6, IL-8, IL-18, TNF-α, and COX-2 mRNA expressions in the spleen compared to the infection group (*p* < 0.001) (Figure 1). In addition, 25–100 mg/kg baicalin reduced the mRNA expression levels of IL-1β, IL-6, IL-8, IL-18, TNF-α, and COX-2 in the spleen compared to the infection group (*p* < 0.05) (Figure 1).

### 3.2. Baicalin Improved MIF/CD74 Axis Levels in Piglet Spleen Elicited by G. parasuis

**Figure 2:**
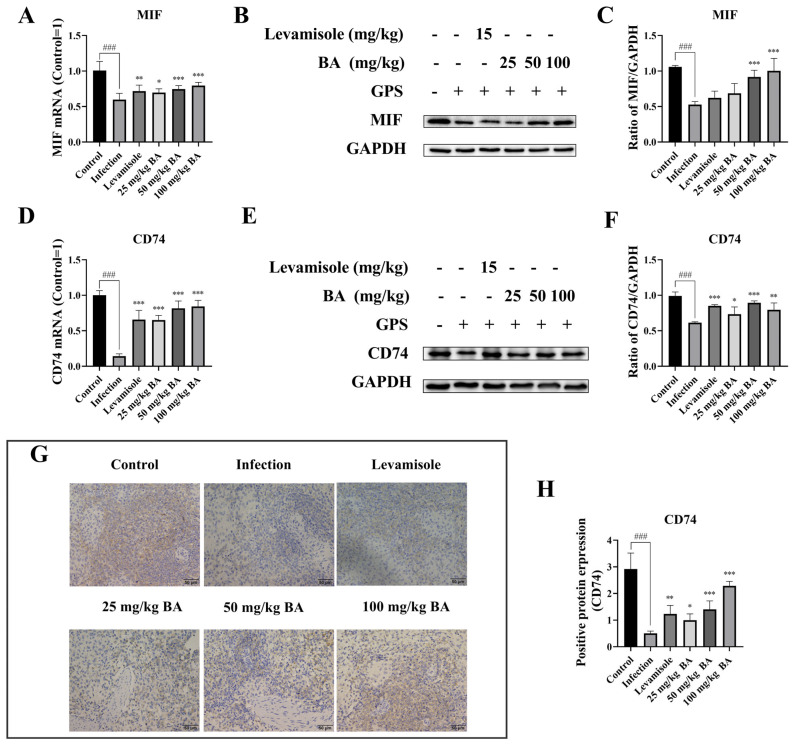
Detection of baicalin on MIF/CD74 axis expression in *G. parasuis* induced piglet spleen. (**A**): MIF mRNA expression level using RT-PCR; (**B**,**C**): MIF protein expression level using western blot; (**D**): CD74 mRNA level by RT-PCR; (**E**,**F**): CD74 protein expression level by western blot; (**G**,**H**): CD74 protein expression level by immunohistochemistry; BA: Baicalin; ^###^ *p* < 0.001 vs. controls; * stands for *p* < 0.05; ** stands for *p* < 0.01; *** stands for *p* < 0.001. Original Western blot images are provided in the Supplementary Materials.

MIF-CD74 signaling blockade on macrophages reduced the immunosuppressive factors contributing to restoring the antitumor immune response in metastatic melanoma [20]. When the piglets were infected with *G. parasuis*, the mRNA expression levels of MIF and CD74 in the spleen were significantly reduced (*p* < 0.001) (Figure 2), while levamisole enhanced the MIF and CD74 mRNA levels in the spleen compared to the infection group (*p* < 0.01) (Figure 2). Doses of 25–100 mg/kg baicalin also increased the mRNA expression levels of MIF and CD74 in the spleen compared to the infection group (*p* < 0.05) (Figure 2). The MIF and CD74 protein expression levels in the spleen were determined using the western blotting method. The data indicated that the protein expression levels of MIF and CD74 in the spleen were downregulated in the infection group (*p* < 0.001), while levamisole and 25–100 mg/kg baicalin could increase CD74 protein level in the spleen compared to the infection group (*p* < 0.05) (Figure 2). Treatment with 50–100 mg/kg baicalin upregulated MIF protein level in the spleen compared to the infection group (*p* < 0.001) (Figure 2).

The CD74 protein expression level was also assessed by immunohistochemistry. The data demonstrated that the *G. parasuis* inhibited the positive protein expression level of CD74 in the spleen compared to the control group, while levamisole and 25–100 mg/kg baicalin promoted the positive protein level of CD74 in the spleen compared to the infection group (*p* < 0.05) (Figure 2).

### 3.3. Baicalin Promoted PI3K/Akt/mTOR Pathway Activation in G. parasuis Triggered Piglet Spleen

**Figure 3:**
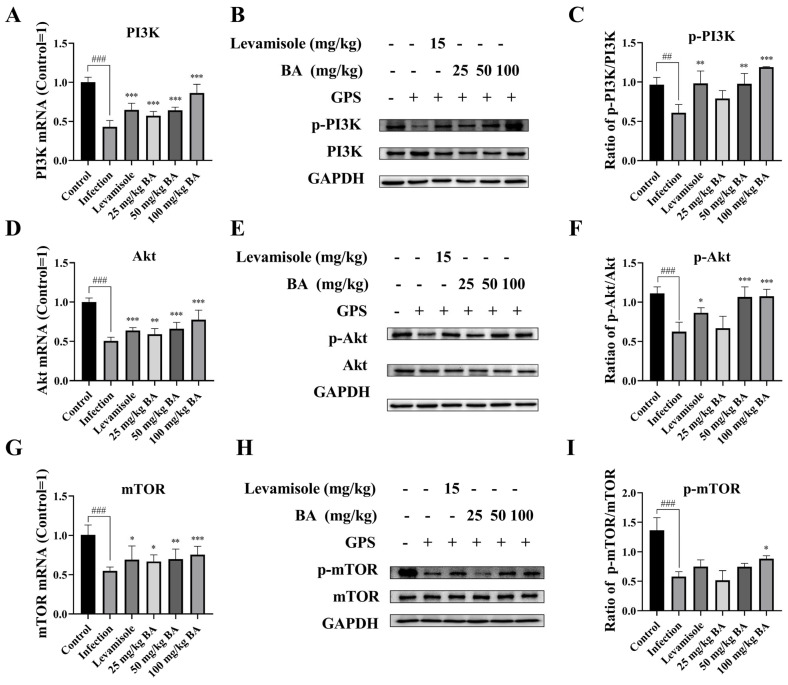
Measurement of the function of baicalin in PI3K/Akt/mTOR activation in *G. parasuis*-infected piglet spleen. (**A**): PI3K mRNA expression level using RT-PCR; (**B**,**C**): p-PI3K protein level using western blot; (**D**): Akt mRNA level using RT-PCR; (**E**,**F**): p-Akt protein expression level via western blot; (**G**): mTOR mRNA expression level via RT-PCR; (**H**,**I**): p-mTOR protein expression level via western blot; BA: Baicalin; ^##^ *p* < 0.01 vs. controls; ^###^ *p* < 0.001 vs. controls; * stands for *p* < 0.05; ** stands for *p* < 0.01; *** stands for *p* < 0.001. Original Western blot images are provided in the Supplementary Materials.

Pieces of evidence have shown that a large number of natural products were targeting PI3K/AKT/mTOR-induced autophagy, leading to diminishing tumor growth [41]; thus, we explored the efficacy of baicalin on PI3K/Akt/mTOR signaling activation. The data indicated that the PI3K, Akt, and mTOR mRNA levels were significantly decreased in the spleen when the piglets were challenged by *G. parasuis* (*p* < 0.001) (Figure 3). The PI3K, Akt, and mTOR mRNA levels treated with levamisole or baicalin were significantly increased compared to the infection group (*p* < 0.05) (Figure 3). In addition, p-PI3K, p-Akt, and p-mTOR protein levels were also declined in the infection group compared to the control group (*p* < 0.01) (Figure 3), while levamisole or 50–100 mg/kg baicalin alleviated the decrease in the protein levels of p-PI3Kand p-Akt (*p* < 0.05) (Figure 3). Dosages of 100 mg/kg baicalin increased p-mTOR protein levels, in contrast with the infection group (*p* < 0.05) (Figure 3).

### 3.4. Baicalin Regulated the RAF/MEK/ERK Signaling Activation in G. parasuis Triggered Piglet Spleen

**Figure 4:**
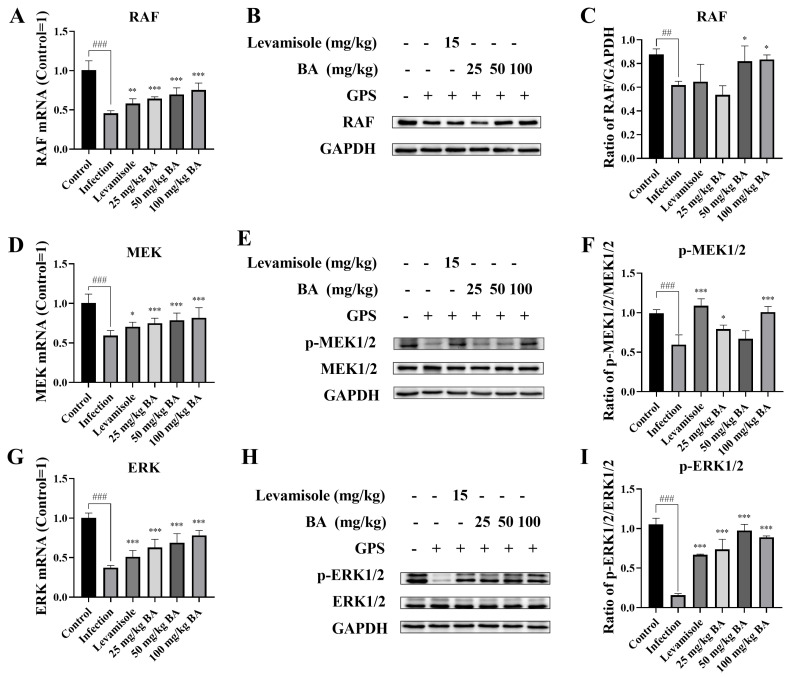
The efficacy of baicalin on RAF/MEK/ERK activation in *G. parasuis*-infected piglet spleen. (**A**): RAF mRNA expression level using RT-PCR; (**B**,**C**): RAF protein level using western blot; (**D**): MEK mRNA level using RT-PCR; (**E**,**F**): p-MEK1/2 protein level via western blot; (**G**): ERK mRNA level via RT-PCR; (**H**,**I**): p-ERK1/2 protein expression level via western blot; BA: Baicalin; ^##^ *p* < 0.01 vs. controls; ^###^ *p* < 0.001 vs. controls; * stands for *p* < 0.05; ** stands for *p* < 0.01; *** stands for *p* < 0.001. Original Western blot images are provided in the Supplementary Materials.

Previous research reported that MIF activated the CD74-dependent “sustained” activation of RAF/MEK/ERK involved in apoptosis and autophagy [42]. Thus, the RAF, MEK, and ERK levels were evaluated. The data demonstrated that *G. parasuis* challenge declined the RAF, MEK, and ERK mRNA levels in the spleen (*p* < 0.001) (Figure 4). Levamisole and 25–100 mg/kg baicalin promoted RAF, MEK, and ERK mRNA levels, in contrast with the infection group (*p* < 0.01) (Figure 4). The RAF, p-MEK, and p-ERK protein levels were suppressed, in contrast with the control group (*p* < 0.01) (Figure 4). Levamisole induced MEK and ERK phosphorylation levels in the spleen (*p* < 0.001) (Figure 4). Furthermore, 50–100 mg/kg baicalin also reversed the effect on RAF and p-ERK protein levels, in contrast with the infection group (*p* < 0.05) (Figure 4).

### 3.5. Baicalin Modified Autophagy-Related Proteins Beclin-1, P62, and LC3B Expression in G. parasuis-Triggered Piglet Spleens

**Figure 5:**
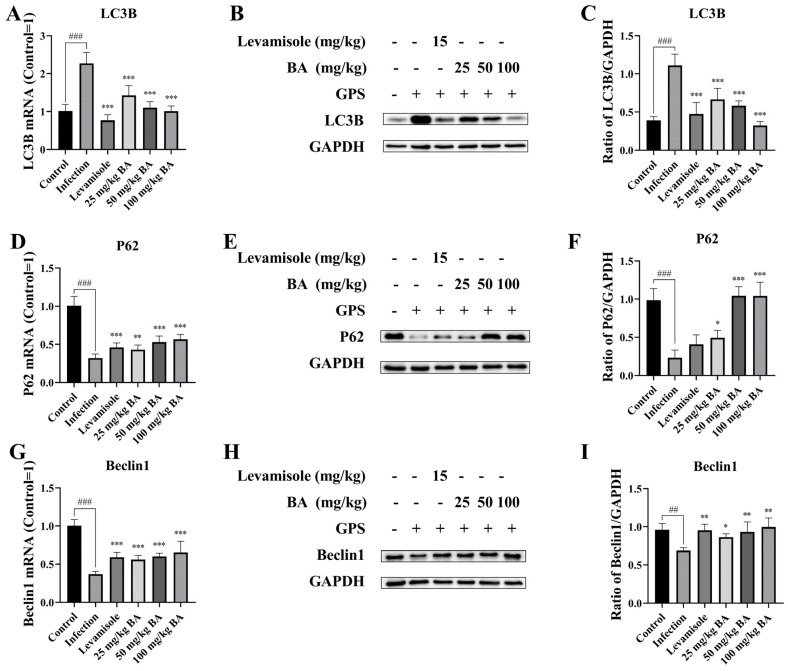
Assessment of the effect of baicalin on autophagy regulation in *G. parasuis*-infected piglet spleen. The levels of the autophagy-related proteins Beclin-1, P62, and LC3B were determined using RT-PCR and western blot. (**A**): LC3B mRNA expression level via RT-PCR; (**B**,**C**): LC3B protein expression level via western blot; (**D**): P62 mRNA expression level via RT-PCR; (**E**,**F**): P62 protein expression level via western blot; (**G**): Beclin-1 mRNA expression level via RT-PCR; (**H**,**I**): Beclin-1 protein level via western blot; BA: Baicalin; ^##^ *p* < 0.01 vs. controls; ^###^ *p* < 0.001 vs. controls; * stands for *p* < 0.05; ** stands for *p* < 0.01; *** stands for *p* < 0.001. Original Western blot images are provided in the Supplementary Materials.

PI3K/Akt/mTOR and RAF/MEK/ERK signaling activation could lead to autophagy [43]. The levels of the autophagy-related proteins Beclin-1, P62, and LC3B in the spleen were determined using the RT-PCR and western blotting methods. The data indicated that at the mRNA and protein level, *G. parasuis* induced the LC3B expression level and downregulated P62 and Beclin-1 expression levels in the spleen from the infection group, in contrast with the control group (*p* < 0.001) (Figure 5). Levamisole treatment reduced the LC3B mRNA and protein levels, upregulated Beclin-1 mRNA and protein levels, and increased P62 mRNA expression level in the spleen, in contrast with the infection group (*p* < 0.01) (Figure 5). Dosages of 25–100 mg/kg baicalin also suppressed the mRNA and protein levels of LC3B and triggered P62, Beclin-1 mRNA, and protein levels in the spleen (*p* < 0.05) (Figure 5).

### 3.6. Baicalin and Levamisole Promoted M2 Polarization to M1 Polarization in G. parasuis-Triggered Piglet Spleens

**Figure 6:**
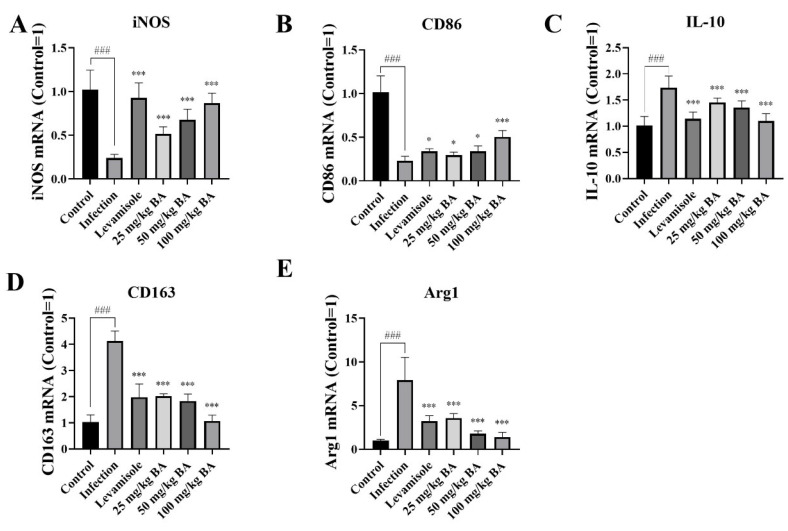
Determination of the effect of baicalin on polarization change in *G. parasuis*-infected piglet spleen. The iNOS (**A**), CD86 (**B**) (M1 markers), IL-10 (**C**), CD163 (**D**), and Arg1 (**E**) (M2 markers) mRNA levels were determined via RT-PCR. BA: Baicalin; ^###^ *p* < 0.001 vs. controls; * stands for *p* < 0.05; *** stands for *p* < 0.001.

Targeting MIF-CD74 enhanced microglia M1 polarization in non-small cell lung cancer [44]. To investigate the immunomodulatory effect of the MIF/CD74 axis in the spleen of *G. parasuis*-infected piglets, the M1 and M2 phenotype markers were detected. The RT-PCR results demonstrated that the *G. parasuis* remarkably decreased iNOS and CD86 expression (M1 markers) and increased ARG1, IL-10, and CD163 expression (M2 markers) in the spleen (*p* < 0.05) (Figure 6). Levamisole and 25–100 mg/kg baicalin upregulated the mRNA expression of iNOS and CD86 and attenuated the levels of ARG1, IL-10, and CD163 in the spleen, in contrast with the infection group (*p* < 0.05) (Figure 6).

### 3.7. Baicalin Enhanced CD3, CD4, CD8, and TIM3 Levels in G. parasuis-Triggered Piglet Spleens

**Figure 7:**
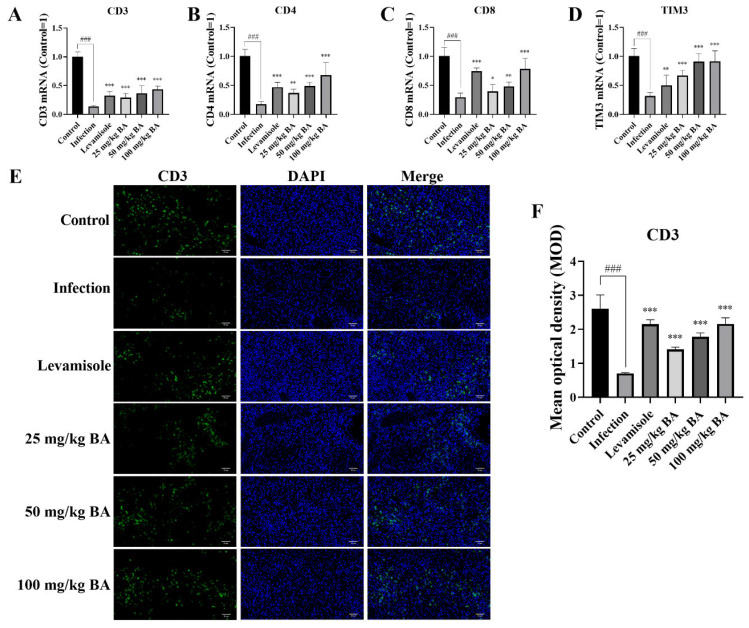
The effect of baicalin on CD3, CD4, CD8, and TIM3 levels in *G. parasuis*-infected piglet spleen. The CD3 (**A**), CD4 (**B**), CD8 (**C**), and TIM3 (**D**) levels were determined using RT-PCR. The CD3 protein expression level (**E**,**F**) was measured via immunofluorometric assay. BA: Baicalin; ^###^ *p* < 0.001 vs. controls; * stands for *p* < 0.05; ** stands for *p* < 0.01; *** stands for *p* < 0.001.

Previous research reported that tumor-associated M2 macrophages promoted tumor growth and suppressed immune response [45]. The decrease in the number of CD3^+^, CD4^+,^ and CD8^+^ T cells is one important reason causing host immunosuppression [46]; thus, we determined the CD3, CD4, CD8, and TIM3 levels in *G. parasuis*-infected spleen. The results showed that, after *G. parasuis* challenge, the CD3, CD4, CD8, and TIM3 mRNA levels were decreased, in contrast with the control group (*p* < 0.001) (Figure 7). When the piglets were pretreated with levamisole or 25–100 mg/kg baicalin, the CD3, CD4, CD8, and TIM3 mRNA levels were increased, in contrast with the infection group (*p* < 0.05) (Figure 7), suggesting that levamisole and baicalin could modify T cell differentiation.

We also detected CD3 expression using an immunofluorometric assay. The results demonstrated that the mean optical density of CD3 in the infection group was significantly decreased compared to that in the control group (*p* < 0.001) (Figure 7). Levamisole and 25–100 mg/kg baicalin enhanced the mean optical density of CD3 compared to the control group (*p* < 0.001) (Figure 7).

## 4. Discussion

*G. parasuis* is a Gram-negative bacterium, and it is a common flora in the upper respiratory tract of pigs [47]. Under certain stress conditions, *G. parasuis* can cause systemic infections characterized by arthritis, meningitis, and polyserositis, known as Glässer’s disease [48]. This disease is considered a major cause of pig deaths, resulting in serious losses to the global pig production [1]. *G. parasuis* infection can cause piglet immunosuppression; however, the immunosuppression mechanism remains unknown; thus, we used the piglet model to assess the molecular mechanism of immunosuppression caused by *G. parasuis*.

Our previous studies have shown that the activation of the PD-1/PD-L1 axis triggered by *G. parasuis* could lead to piglet immunosuppression [9]. In this study, we found that MIF/CD74 axis activation induced by *G. parasuis* could result in piglet immunosuppression. MIF/CD74 axis, as an immune checkpoint, has been confirmed to promote immunosuppression and evade immune surveillance [49]. MIF-CD74 signaling blockade on macrophages restored the antitumor immune response against metastatic melanoma [20]. Known as a macrophage migration inhibitor, MIF attracted and retained activated immune cells from the periphery to inflammatory tissue [50]. MIF is involved in immune regulation, inflammatory response, and cell proliferation [51]. The biological function of MIF is mainly mediated by its main receptor, CD74 [52]. As a receptor for MIF, CD74 is mainly expressed in macrophages [53]. MIF and the receptor CD74 can be used as a promising target to treat Ewing sarcoma, which is infiltrated by immunosuppressive myeloid populations [54]. CD74 promoted immunosuppressive tumor microenvironment formation in breast cancer in mice; therefore, CD74 might be considered a novel therapeutic target in breast cancer [55]. Therefore, in this study, we investigated the effect of baicalin on MIF/CD74 axis expression in the spleen triggered by *G. parasuis*. Our results indicated that baicalin regulated MIF/CD74 axis expression in the spleen triggered by *G. parasuis*, which suggested that MIF and CD74 might serve as new targets to treat *G. parasuis* infection.

Autophagy is a process by which cells degrade and recycle proteins and organelles to maintain intracellular homeostasis [30]. Autophagy is a key molecular pathway to preserve cellular and organismal homeostasis [56]. Autophagy offers an adaptive function to protect organisms against diverse pathologies, such as infections [57]. LC3B, Beclin-1, and p62 play key functions in the induction of autophagy during the disease state [58]. The autophagy-related protein LC3B accelerates PRMT1 mRNA decay, thereby enhancing autophagy [59]. P62 has been demonstrated to regulate autophagy [60]. This study showed that *G. parasuis* induced autophagy, and baicalin and levamisole treatment inhibited the autophagy phenomenon. Previous reports showed that MIF promoted cardiac stem cell proliferation and endothelial differentiation via PI3K/Akt/mTOR pathway activation [61]. The PI3K-Akt-mTOR pathway activation promoted necrotic cell death by suppressing autophagy [62]. Scoparone reduced hepatic inflammation and regulated autophagy in nonalcoholic steatohepatitis mice through PI3K/AKT/mTOR pathway regulation in macrophages [63]. Thus, we assessed the effect of baicalin on the PI3K/AKT/mTOR pathway and autophagy activation in the spleen of piglets challenged with *G. parasuis*. Our data demonstrated that baicalin regulated the PI3K/AKT/mTOR pathway and reduced autophagy in piglets challenged with *G. parasuis*, which enhances the new function of baicalin in autophagy regulation.

The RAF-MEK-ERK pathway is a characterized MAPK signaling pathway that participates in cell proliferation and survival [64]. Fibroblast GSK-3α promoted fibrosis via the RAF-MEK-ERK pathway in the injured heart [65]. The EIF3H-HAX1 increased RAF-MEK-ERK signaling activity to enhance colorectal cancer progression [66]. In addition, many studies reported that RAF/MEK/ERK signaling is associated with autophagy modification [67]. Geniposide alleviated lipopolysaccharide-induced murine kidney podocyte apoptosis via promoting RAF/MEK/ERK-mediated autophagy [68]. The use of 5-aminolevulinic acid photodynamic therapy diminished HPV viral load through autophagy by modifying RAF/MEK/ERK in HeLa cells [69]. Thus, we inferred that baicalin could modify autophagy through RAF/MEK/ERK pathway regulation, but the specific mechanism needs further research.

Macrophages are usually classified into two categories, which are named M1 macrophages and M2 macrophages [70]. Previous research reported that M1 macrophages and M2 macrophages are associated with inflammation responses [71]. M1 and M2 macrophage polarization imbalance might trigger pathological consequences and lead to diseases [72]. Previous research showed that CD5L promoted M2 macrophage polarization by an autophagy-mediated ID3 increase [73]. Puerarin induced macrophage M2 polarization to display antinon-alcoholic steatohepatitis activity through autophagy activation [74]. Baicalin induces M1 polarization to exert antiviral effects [75]. Thus, we explored the levels of M1 markers (iNOS, CD86) and M2 markers (ARG1, IL-10, CD163). Immunotherapy-activated T cells could recruit and skew late-stage activated M1-like macrophages, critical for cancer immunotherapy [76]. Wnt5a triggered the M2 polarization of tumor-associated macrophages by modifying CaKMII-ERK1/2-STAT3-mediated IL-10 production, leading to the induction of tumor growth and the metastasis of colorectal cancer [77]. The results indicated that M1 was downregulated and M2 was upregulated in the *G. parasuis*-infected spleen, suggesting that infection with *G. parasuis* in piglets induces macrophage polarization towards the M2 phenotype, while baicalin could reverse the M2 phenotype to the M1 phenotype.

Previous research reported that nicotinamide phosphoribosyltransferase-driven tumor-associated macrophage M2 polarization results in an immunosuppressive microenvironment in colorectal cancer [78]. Macrophages activated by the histamine receptor H1 could polarize toward the M2-like immunosuppressive phenotype with the dysfunction of T cells [79]. T cells have an important effect in modifying the immune system balance [80]. Sepsis-associated immunosuppression was correlated to a reduction in the number of T cells [81]. CD4+ T cells have important functions in immunosuppression in inflammatory diseases [82]. Metformin improved cancer immunotherapy by remedying tumor-infiltrating CD8 T lymphocytes from the immunosuppression triggered by hypoxia [83]. T-cell exhaustion is a major reason for immunosuppression in the tumor microenvironment [84]. The T cell immunoglobulin domain and mucin domain 3 (TIM3) served as targets for CD8+ T cell exhaustion [7]. Therefore, the expression levels of CD3, CD4, CD8, and TIM3 in *G. parasuis*-infected piglet spleens were measured, and our results confirmed that infection with *G. parasuis* in piglets could lead to host immunosuppression, and baicalin could alleviate the immunosuppression in piglets caused by *G. parasuis* [35].

Taken together, our study showed that *G. parasuis* infection could promote the MIF/CD74 axis and regulate the PI3K/Akt/mTOR and RAF/MEK/ERK signaling pathways induced autophagy, thereby transforming M1 towards M2 polarization in the spleen, contributing to host immunosuppression, whereas baicalin could reverse this signaling activation in order to reduce host immunosuppression. Our study is the first to report that baicalin could alleviate host immunosuppression by regulating the MIF/CD74 axis-induced polarization of macrophages, which might provide some novel targets to relieve the immunosuppression and inflammation caused by *G. parasuis.* Our future study will explore the therapeutic potential of baicalin in combination with other treatments.

## Figures and Tables

**Table 1:** Primer sequences for qRT-PCR analysis.

Gene		Nucleotide Sequence (5′-3′)	Tm (°C)	Length (bp)
IL-1β	Forward	TCTGCATGAGCTTTGTGCAAG	59.7	155
	Reverse	ACAGGGCAGACTCGAATTCAAC	60.9	
IL-6	Forward	CTTCTGGTGATGGCTACTG	52.7	134
	Reverse	TTGCCGAGGATGTACTTAA	50.0	
IL-8	Forward	ACAGCAGTAACAACAACAAG	50.2	117
	Reverse	GACCAGCACAGGAATGAG	53.2	
IL-10	Forward	CGTGGAGGAGGTGAAGAGTG	55.4	178
	Reverse	TTAGTAGAGTCGTCATCCTGGAAG	55.6	
IL-18	Forward	AGTAACCATATCTGTGCAGTGT	54.0	155
	Reverse	TCTTATCACCATGTCCAGGAAC	53.0	
TNF-α	Forward	CGCTCTTCTGCCTACTGCACTTC	60.7	164
	Reverse	CTGTCCCTCGGCTTTGACATT	57.8	
CD74	Forward	CTGGGAGTGTACCCGAAGC	58.6	104
	Reverse	CGCAGCCAGTTCTCAAAGAG	56.6	
MIF	Forward	GTGCGCCCTTTGCAGTCT	59.5	146
	Reverse	CCGCGTTCATGTCGTAGTAGTT	57.1	
COX-2	Forward	TGTGAAAGGGAGGAAAGA	49.7	133
	Reverse	CTGATGGGTGAAGTGCTG	53.5	
TIM-3	Forward	TCAAGCCTCATCACTTTGG	53.7	145
	Reverse	TGACGGAGCAGTAACACTC	50.8	
PI3K	Forward	TTGCTACAATCAATCGCCAGGAGAC	59.3	147
	Reverse	CTTCCCGTTGTTGCCATCGTTTG	59.7	
Akt	Forward	GGACGGGCACATCAAGATCACTG	60.8	126
	Reverse	TAGTCGTTGTCCTCCAGCACCTC	61.2	
mTOR	Forward	AGTACCTCCAGGACACCATGAACC	60.9	108
	Reverse	CAGACCTCACAGCCACAGAAAGC	61.0	
GAPDH	Forward	GGCACAGTCAAGGCGGAGAAC	61.9	105
	Reverse	AGCACCAGCATCACCCCATTTG	61.0	
LC3B	Forward	AGCCTTCTTCCTGTTAGTG	51.3	135
	Reverse	TTCATTCCGAAAGTCTCC	48.4	
P62	Forward	CCCCAATGTGATCTGTGATG	53.4	126
	Reverse	TTGCTGTGCTCCTTGTGAAT	54.5	
Beclin-1	Forward	TGAGGATACCCAAGCAAG	51.2	146
	Reverse	ATGTGGAGAAAGGCAAGA	50.4	
Inos	Forward	TGGAAGCGGTAACAAAGGA	53.7	482
	Reverse	CACGAGGTCAGGAGGGATT	56.8	
ARG1	Forward	GGACCTGTGCTTTGCTGAT	55.6	122
	Reverse	TTCCGTTCTTCTTGATTTCTG	49.7	
RAF	Forward	CAACACTGATGCTGCTGGTAA	55.4	113
	Reverse	CAGATGGCGACTTGGAATG	53.8	
MEK	Forward	CGTGAATGAGCCACCTCCC	60.7	149
	Reverse	CCACCTCGGACCGTTTGAT	60.2	
ERK	Forward	GCTCTTGAAGACGCAGCAC	58.6	108
	Reverse	CAGCAGGTTGGAAGGTTTG	58.5	
CD86	Forward	TGTGGGATGGTGTCCTTTG	55.0	106
	Reverse	GTTCACTCGCCTTCCTGTT	55.2	
CD3	Forward	GTTTGCTGATGGTGGTGTA	52.4	144
	Reverse	TGGGCTCATAGTCTGGATT	52.5	
CD4	Forward	AGCCTCAGTTACCGAGTT	52.7	138
	Reverse	ATCCTCTTGTCTTCCACTTC	51.1	
CD8	Forward	GTTACATCTCTGGTTACAAGG	49.9	139
	Reverse	AAGAAGACGGACATGAAGTT	50.7	
CD163	Forward	TGCTGTAGTCGCTGTTCT	55.9	117
	Reverse	ACTTTCACCTCCACTCTTC	54.0	

## Data Availability

All relevant data are within the manuscript.

## Supplementary Materials

The following supporting information can be downloaded at: https://www.mdpi.com/article/10.3390/biom15050640/s1. Original Western blot images.
