# Supplementary material for: Baicalin Relieves Glaesserella parasuis-Triggered Immunosuppression Through Polarization via MIF/CD74 Signaling Pathway in Piglets
Source: Biomolecules. 2025 Apr 29;15(5):640. doi: 10.3390/biom15050640 (PMC12108920; doi:10.3390/biom15050640)

Fig S2

Fig S2. B:

GAPDH (repeat 1)

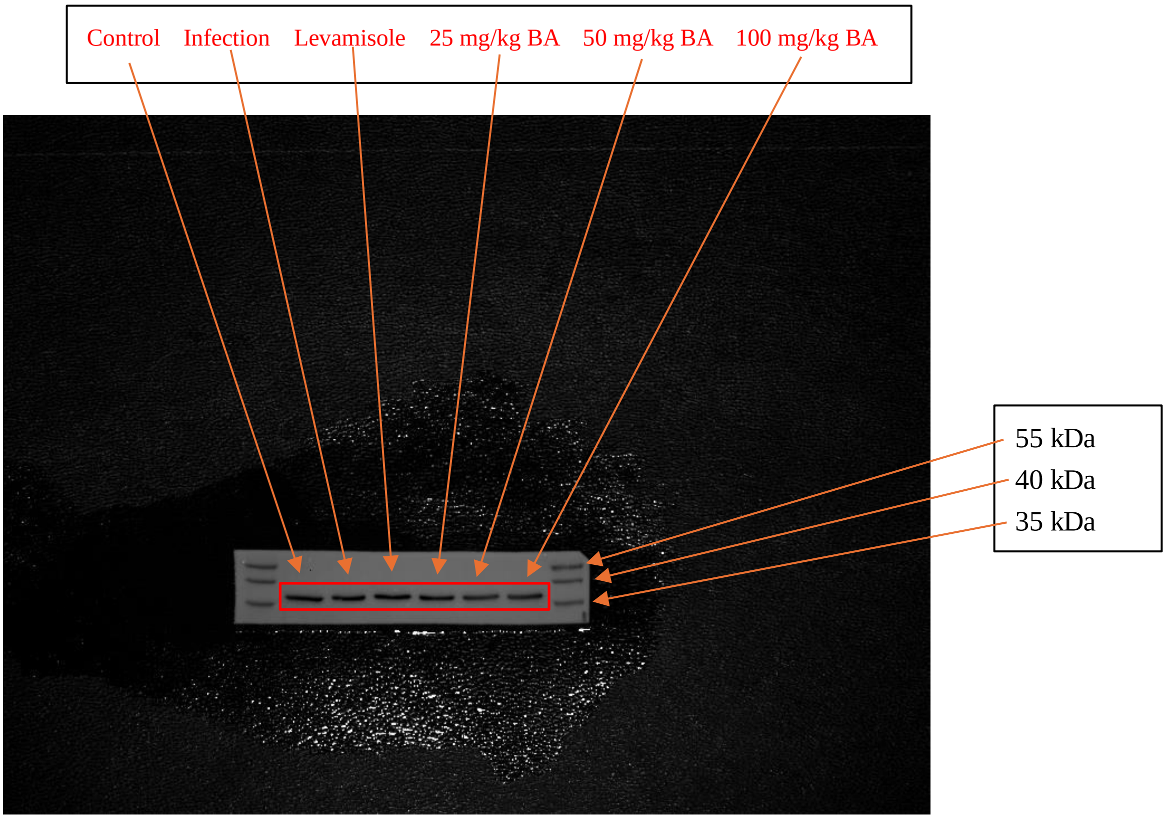

Fig S2

Fig S2. B:

GAPDH (repeat 2)

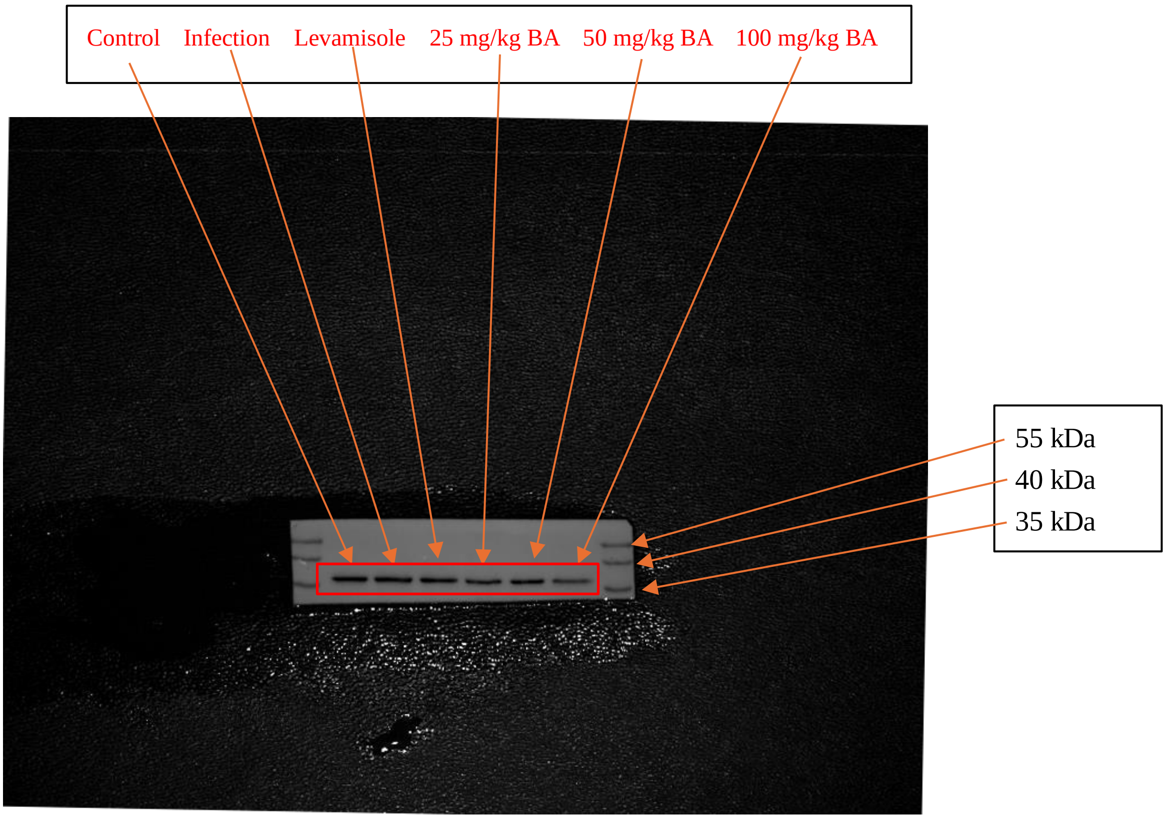

Fig 2

Fig 2. B:

GAPDH (repeat 3)

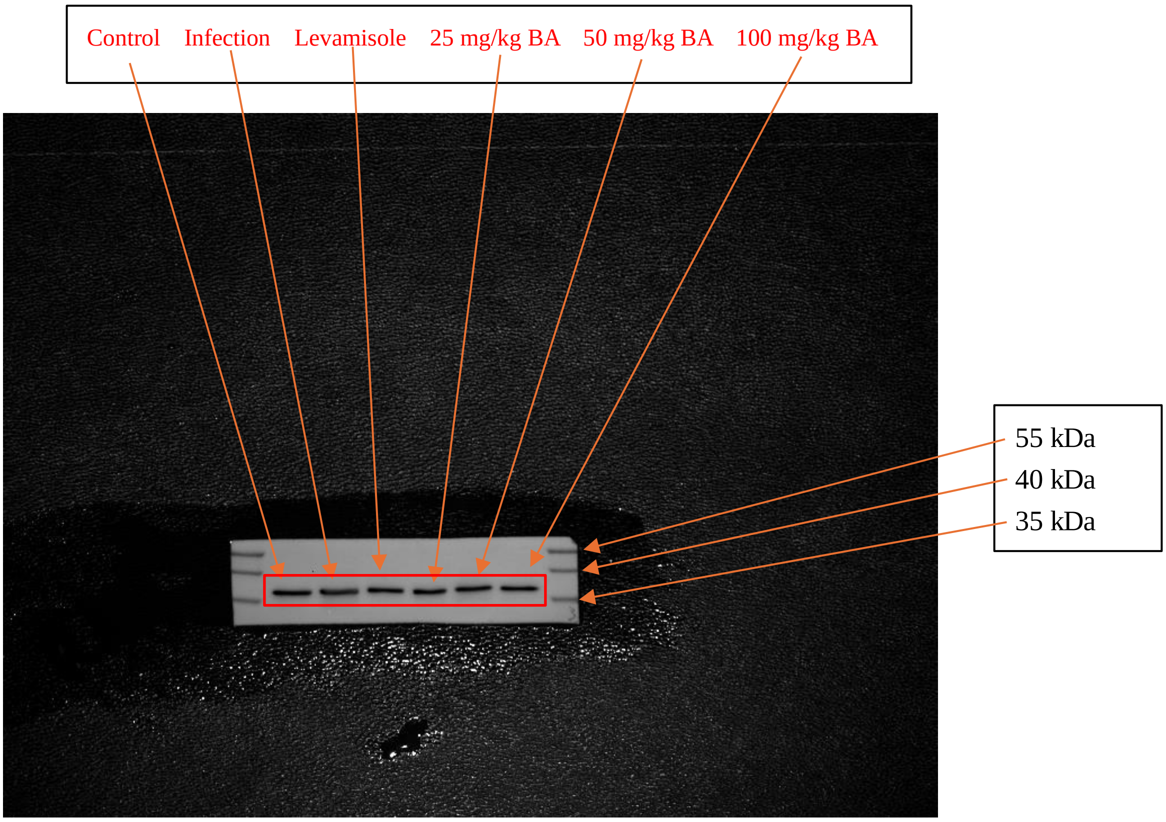

Fig S2

Fig S2. B:

MIF (repeat 1)

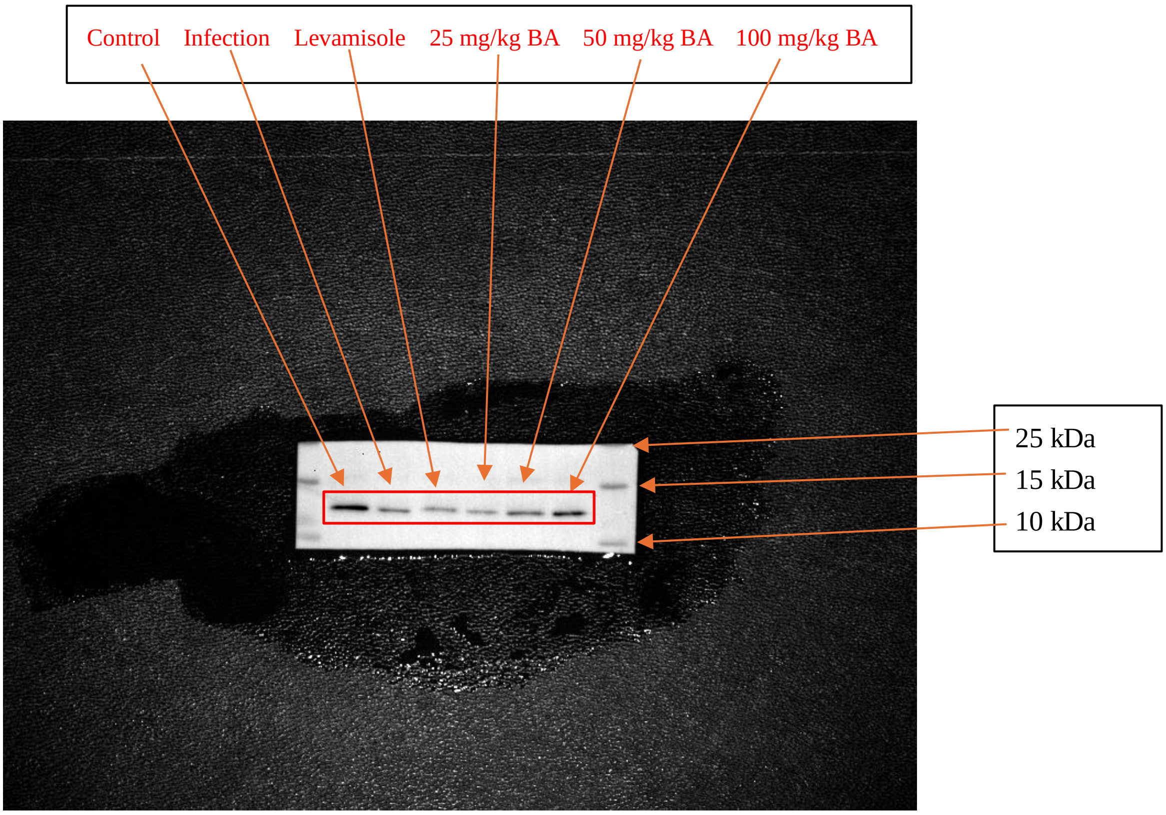

Fig S2

Fig S2. B:

MIF (repeat 2)

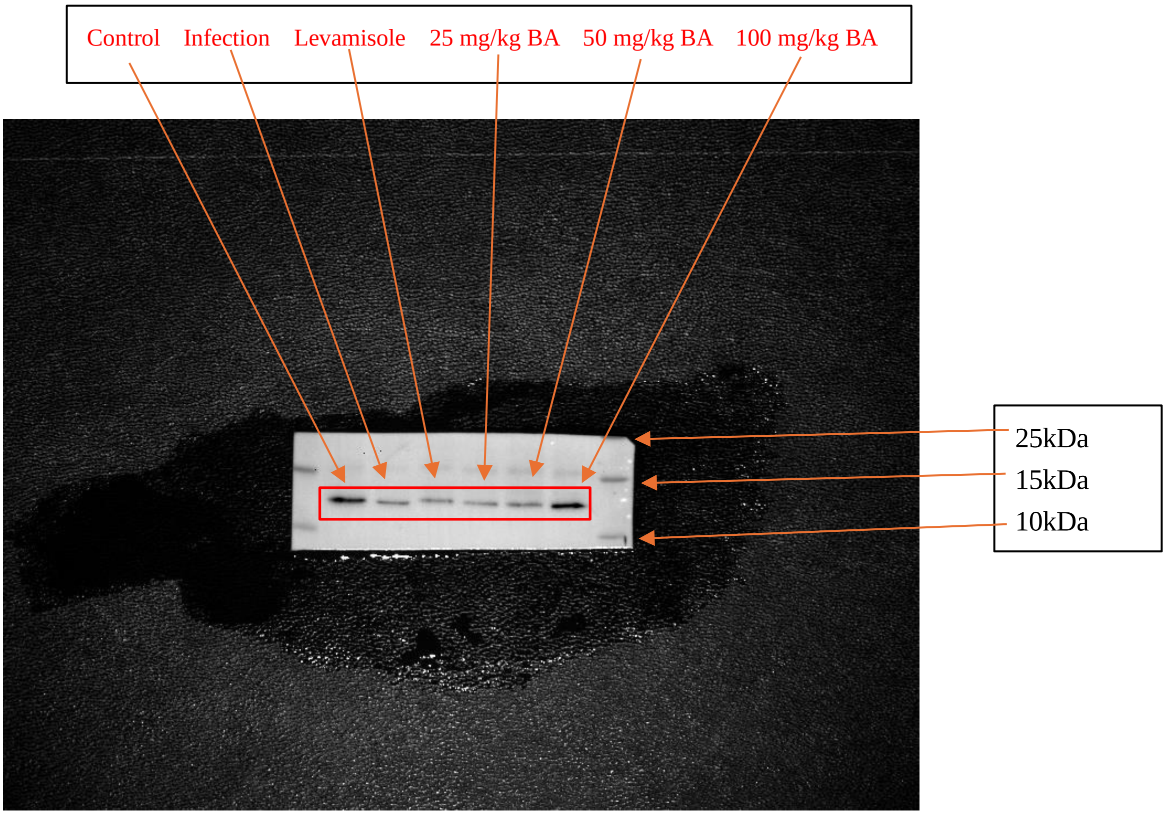

Fig S2

Fig S2. B:

MIF (repeat 3)

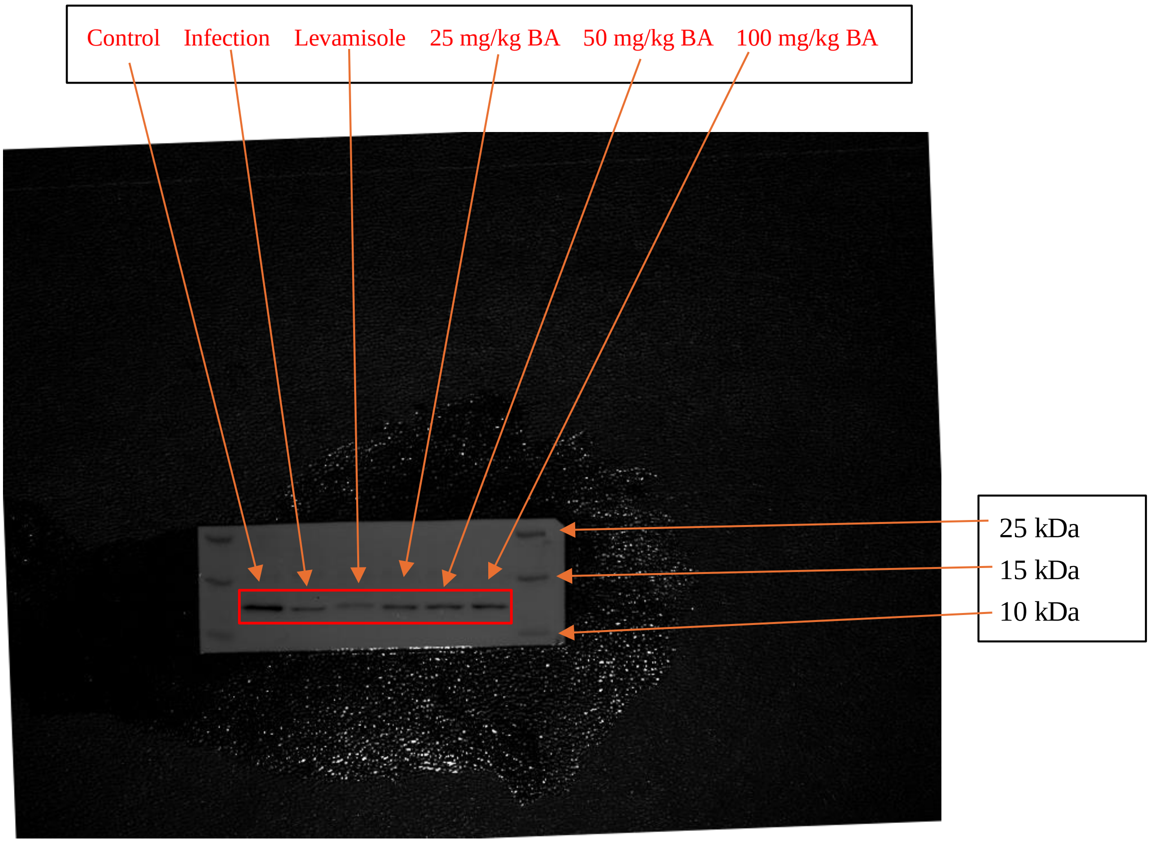

Fig S2

Fig S2. E:

GAPDH (repeat 1)

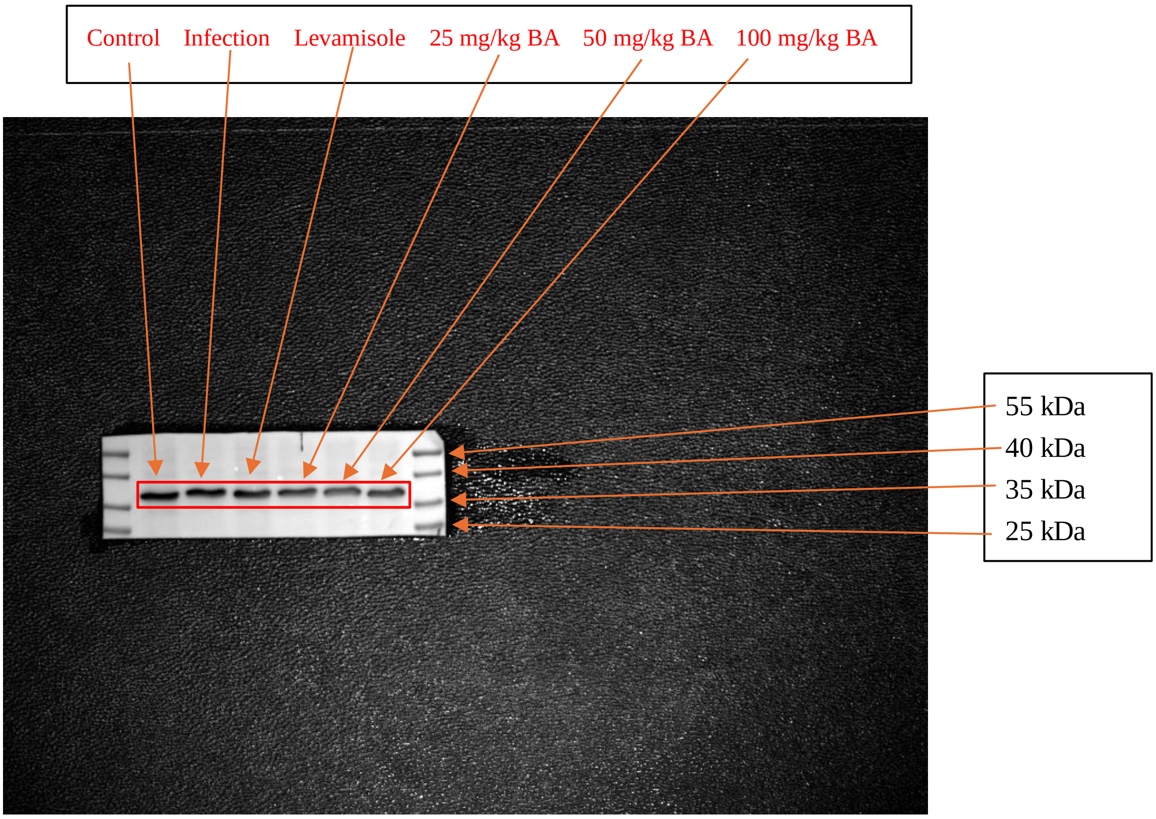

Fig S2

Fig S2. E:

GAPDH (repeat 2)

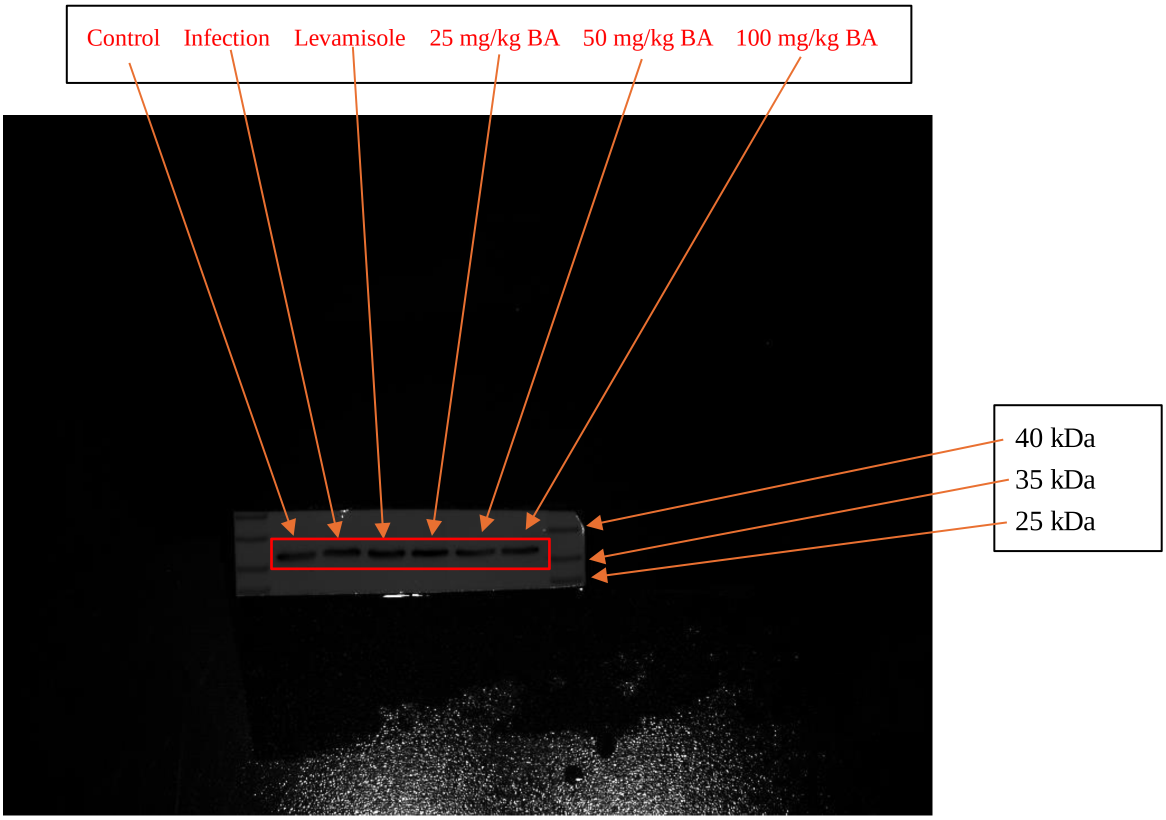

Fig S2

Fig S2. E:

GAPDH (repeat 3)

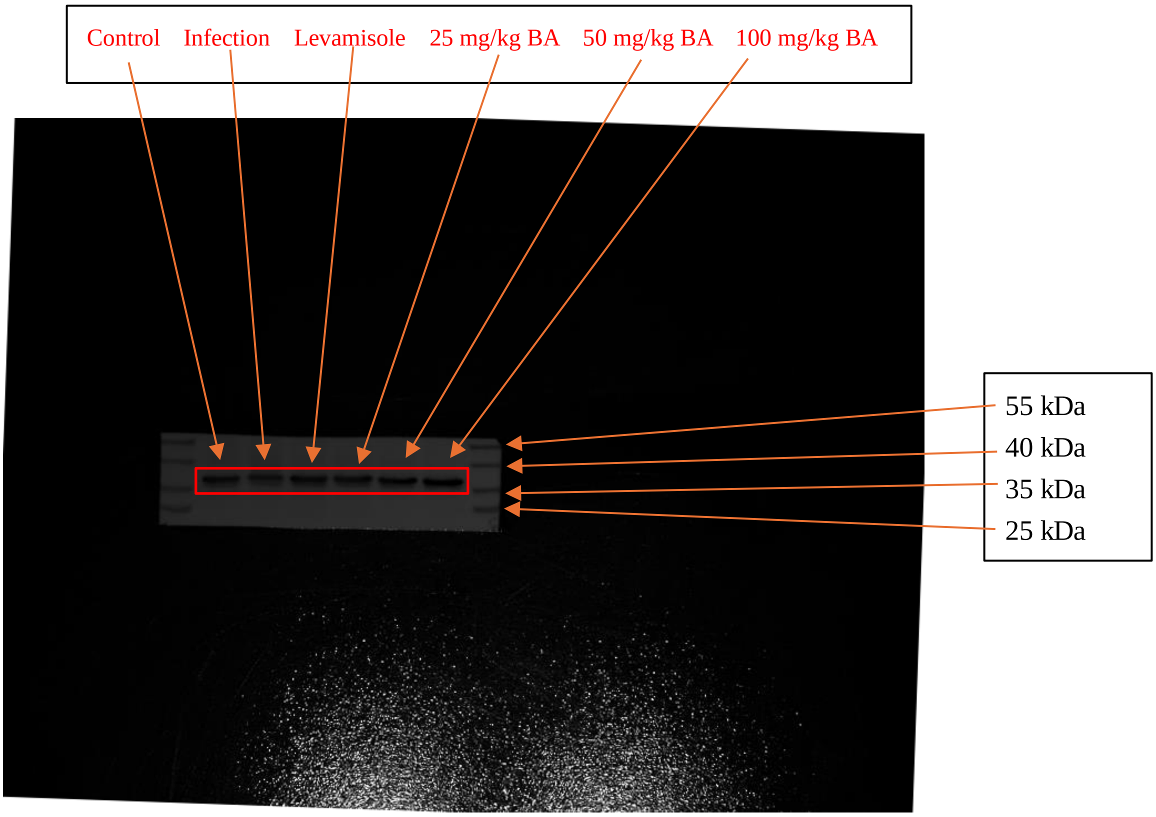

Fig S2

Fig S2. E:

CD74 (repeat 1)

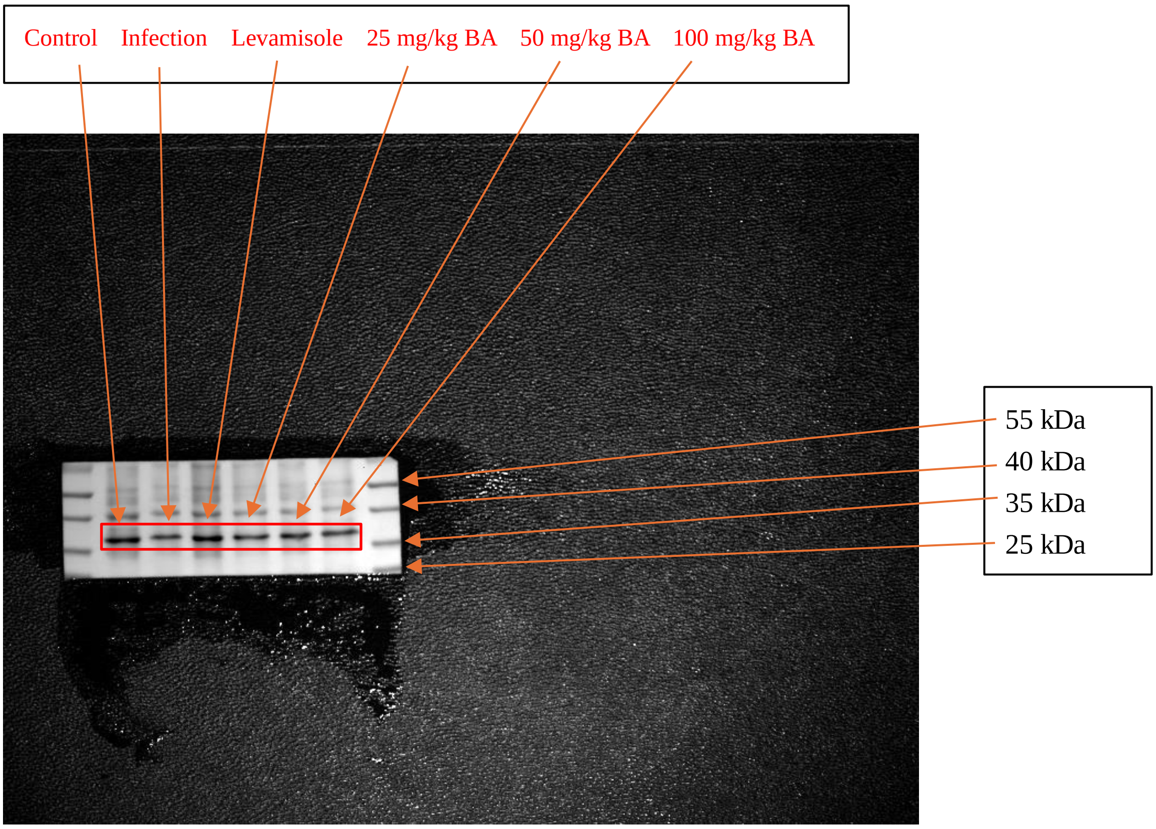

Fig S2

Fig S2. E:

CD74 (repeat 2)

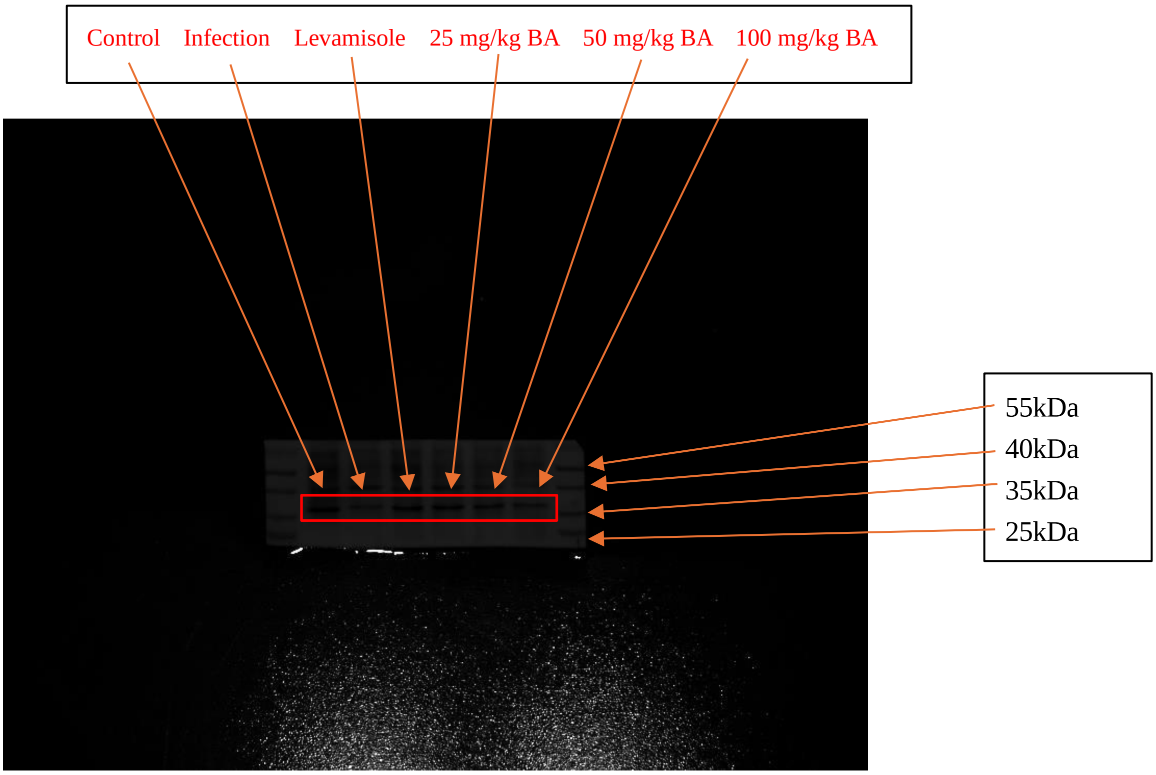

Fig S2

Fig S2. E:

CD74 (repeat 3)

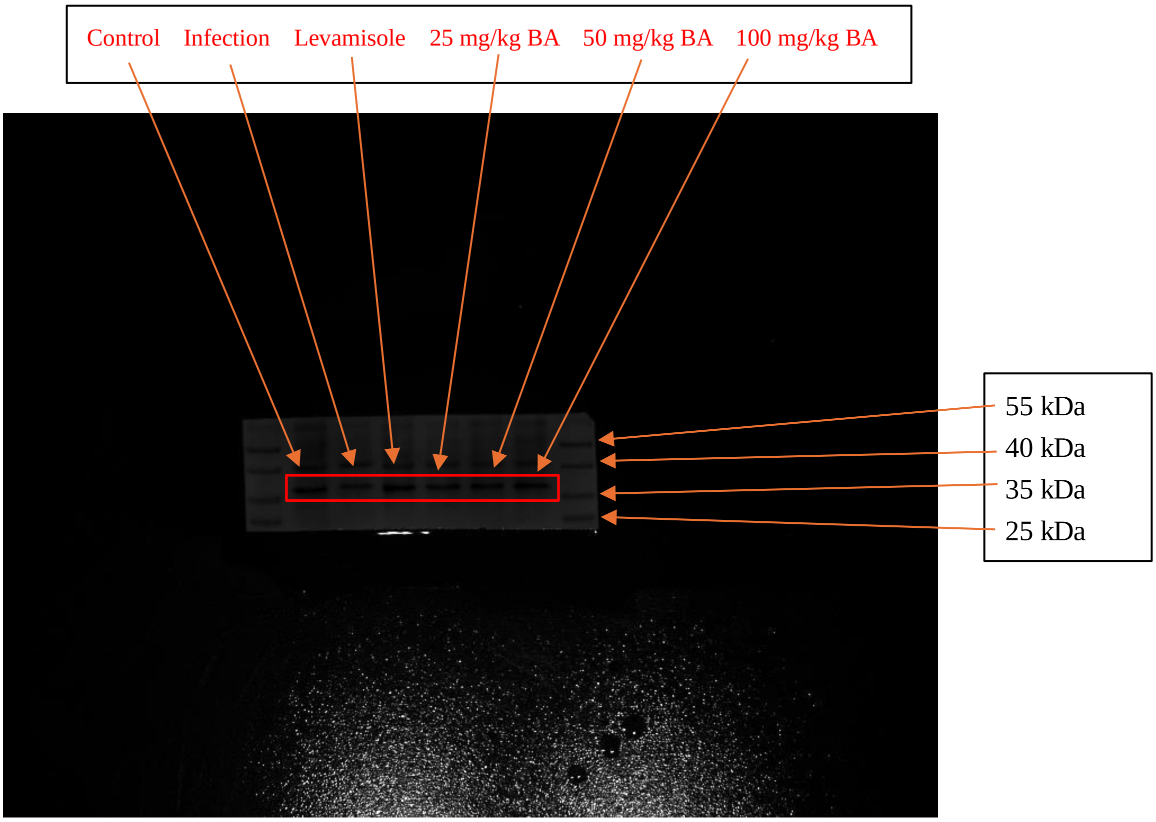

Fig S3

Fig S3. B:

GAPDH (repeat1)

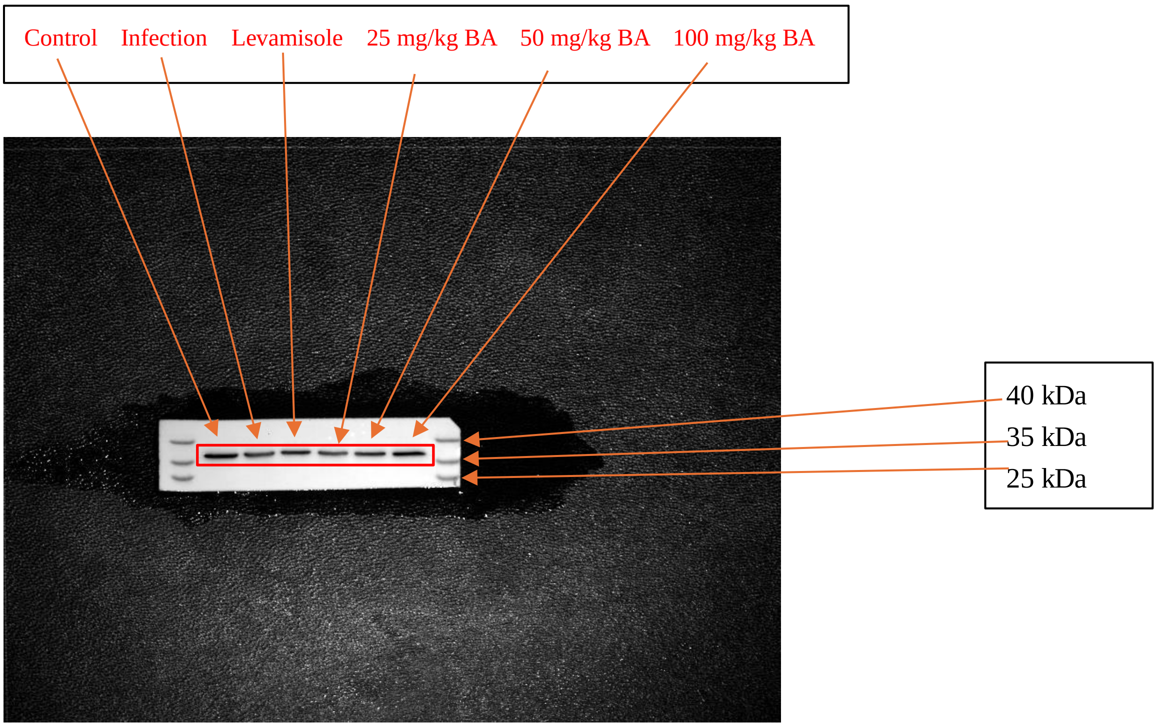

Fig S3

Fig S3. B:

GAPDH (repeat 2)

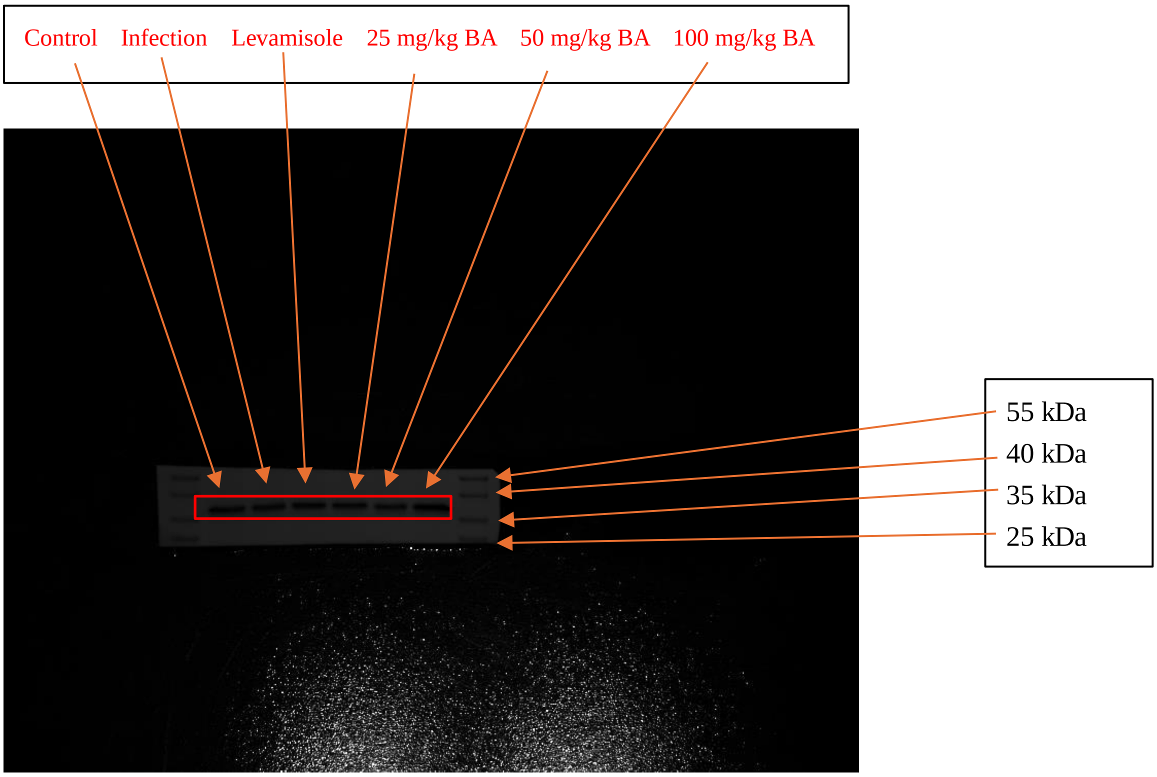

Fig S3

Fig S3. B:

GAPDH (repeat 3)

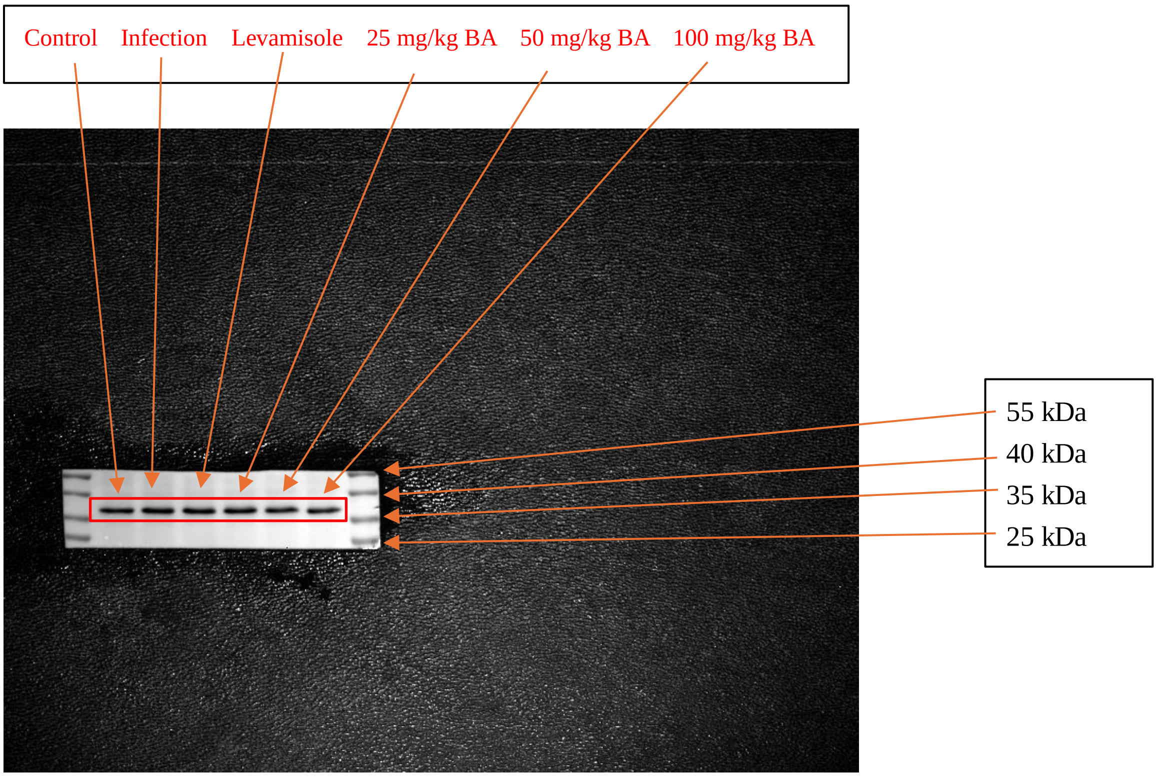

Fig S3

Fig S3. B:

PI3K (repeat 1)

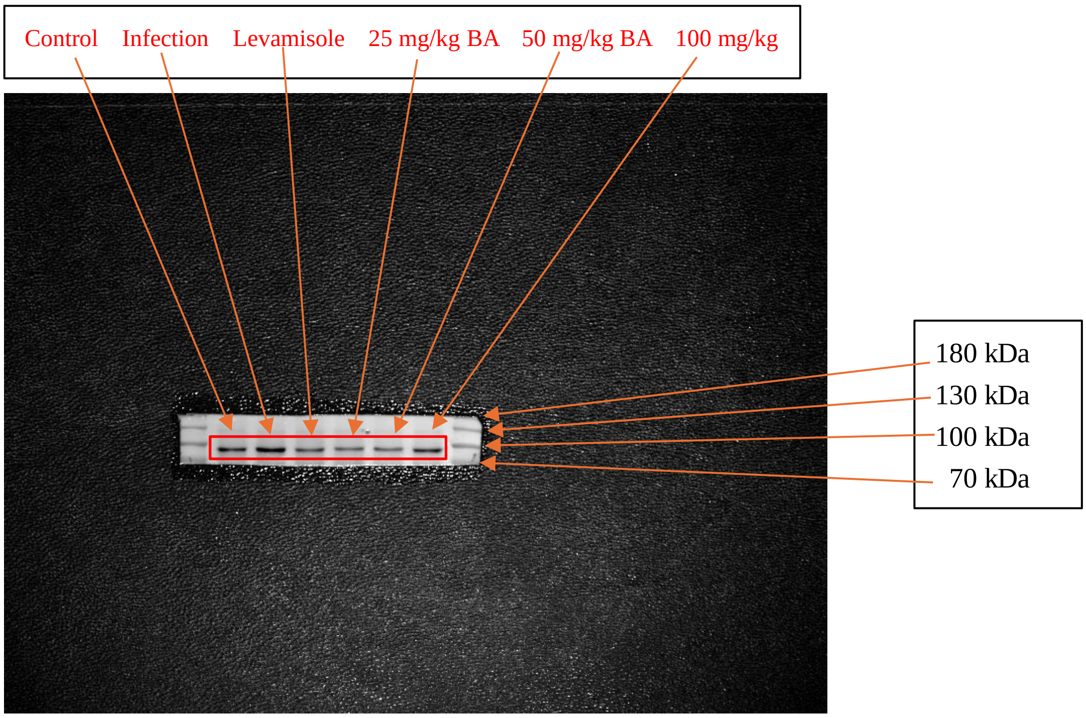

Fig S3

Fig S3. B:

PI3K (repeat 2)

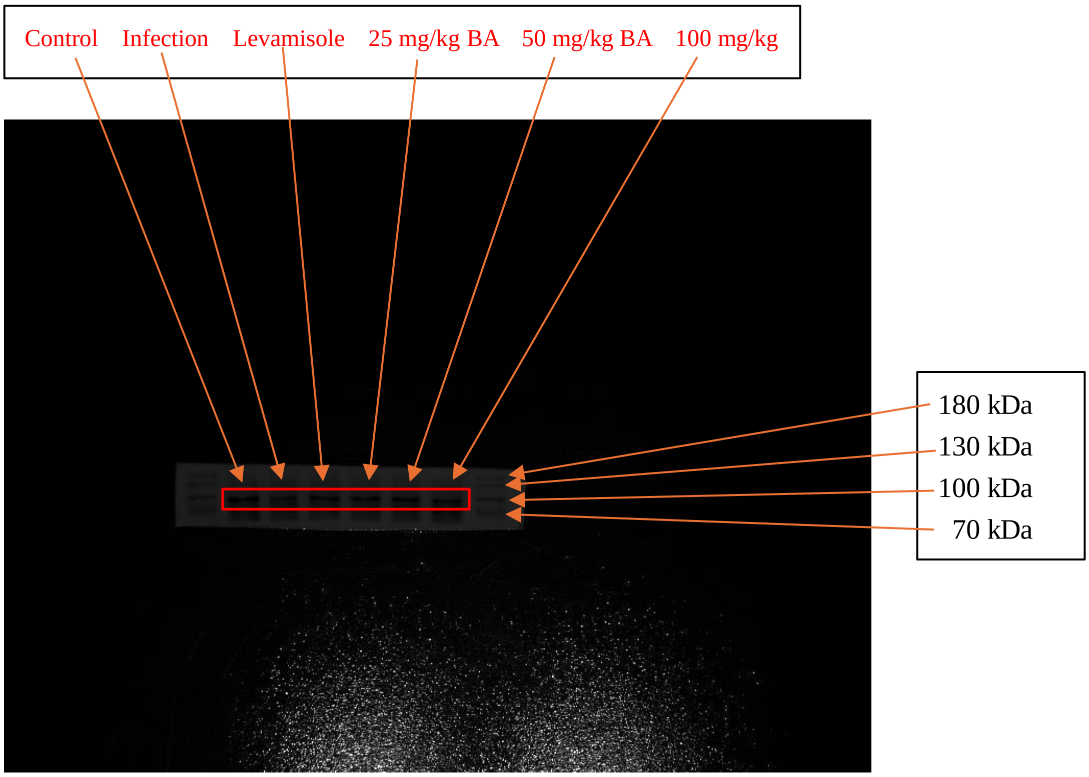

Fig S3

Fig S3. B:

PI3K (repeat 3)

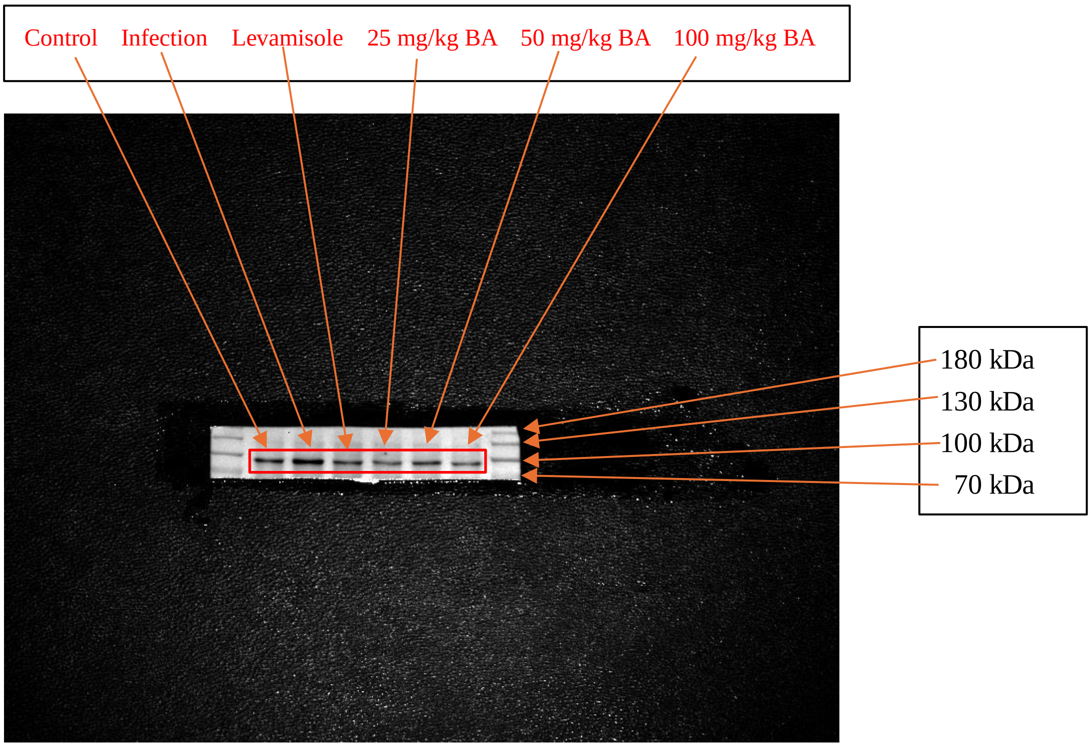

Fig S3

Fig S3. B:

p-PI3K (repeat 1)

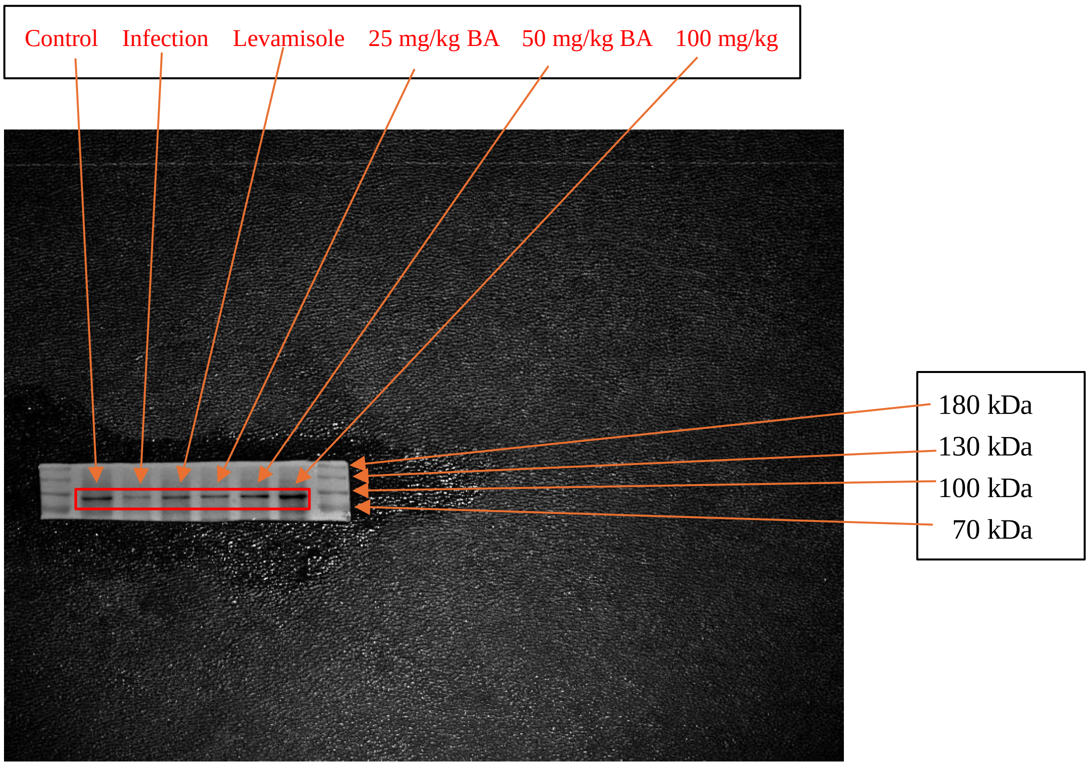

Fig S3

Fig S3. B:

p-PI3K (repeat 2)

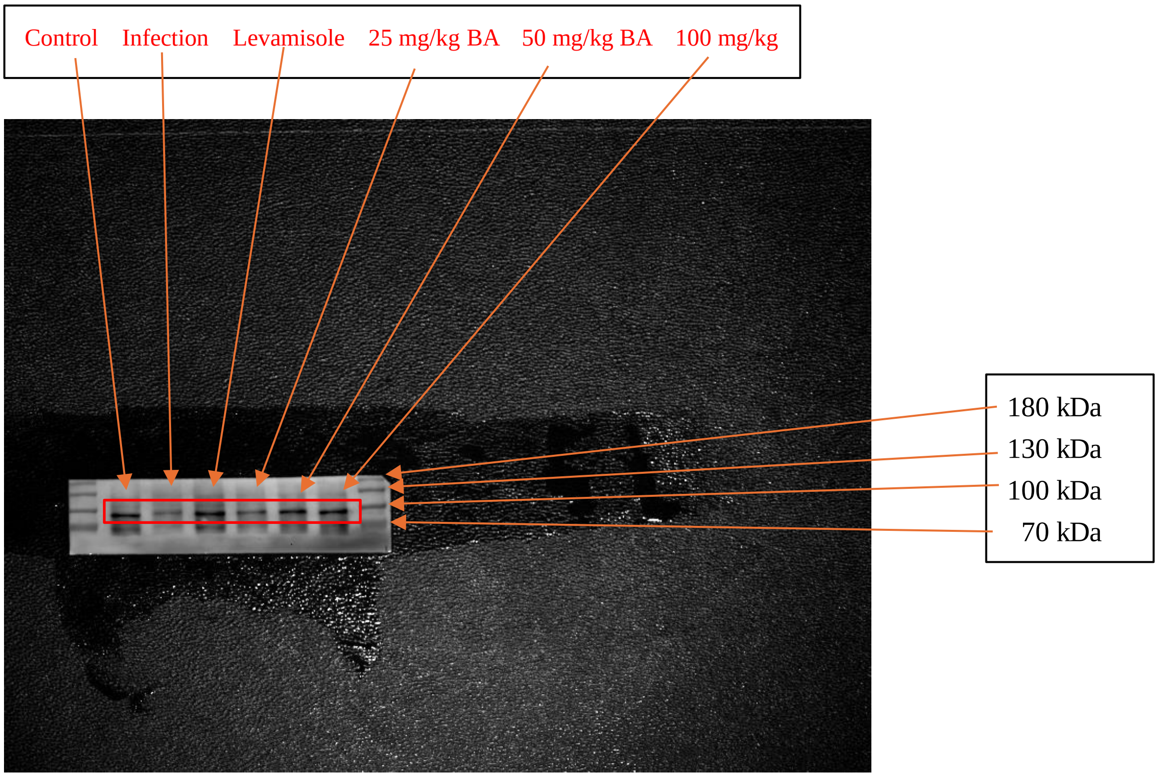

Fig S3

Fig S3. B:

p-PI3K (repeat 3)

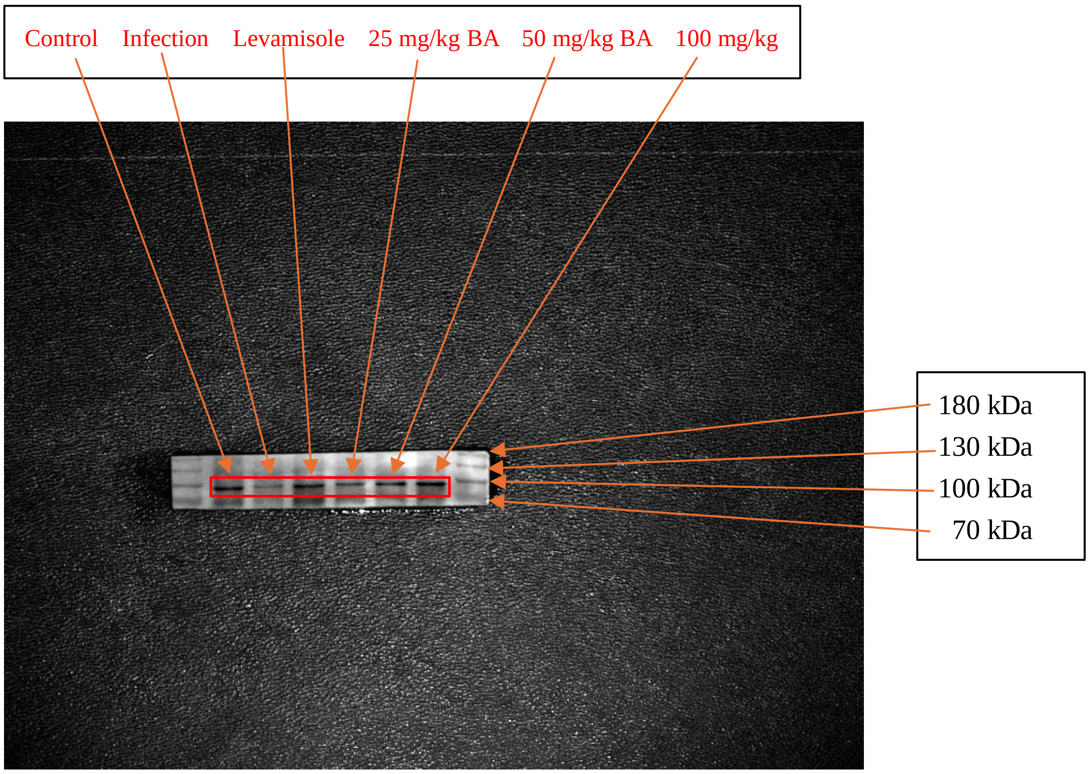

Fig S3

Fig S3. E:

GAPDH (repeat 1)

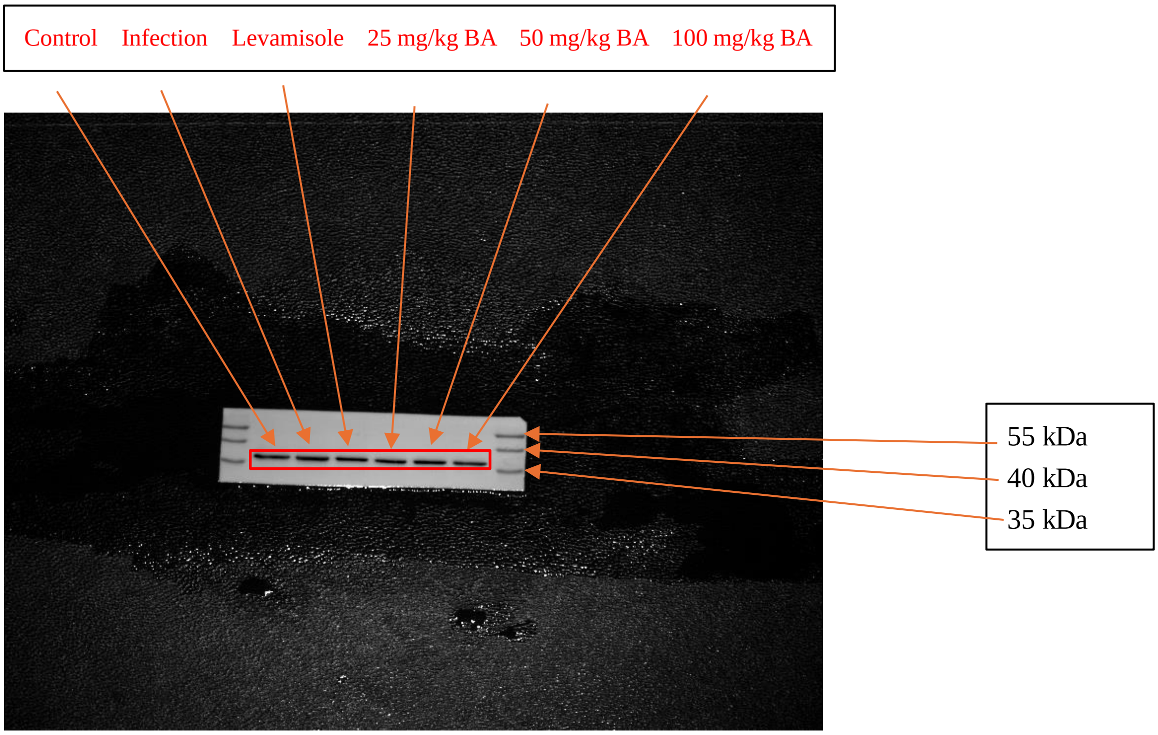

Fig S3

Fig S3. E:

GAPDH (repeat 2)

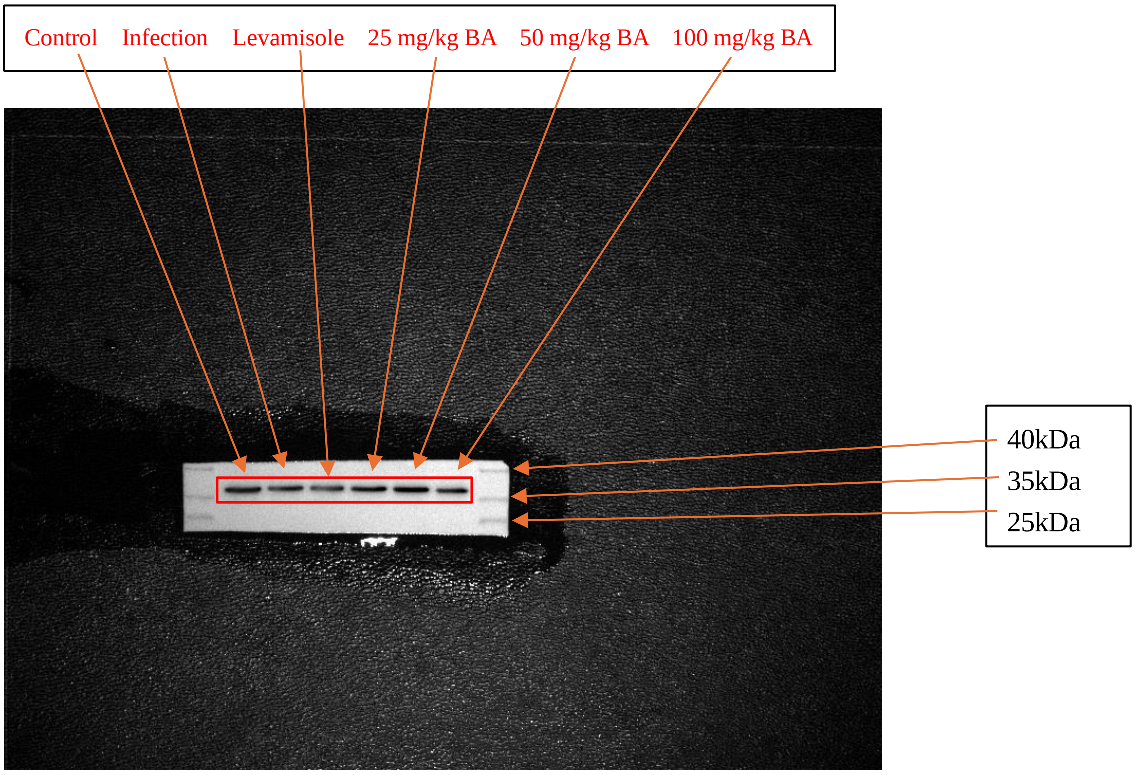

Fig S3

Fig S3. E:

GAPDH (repeat 3)

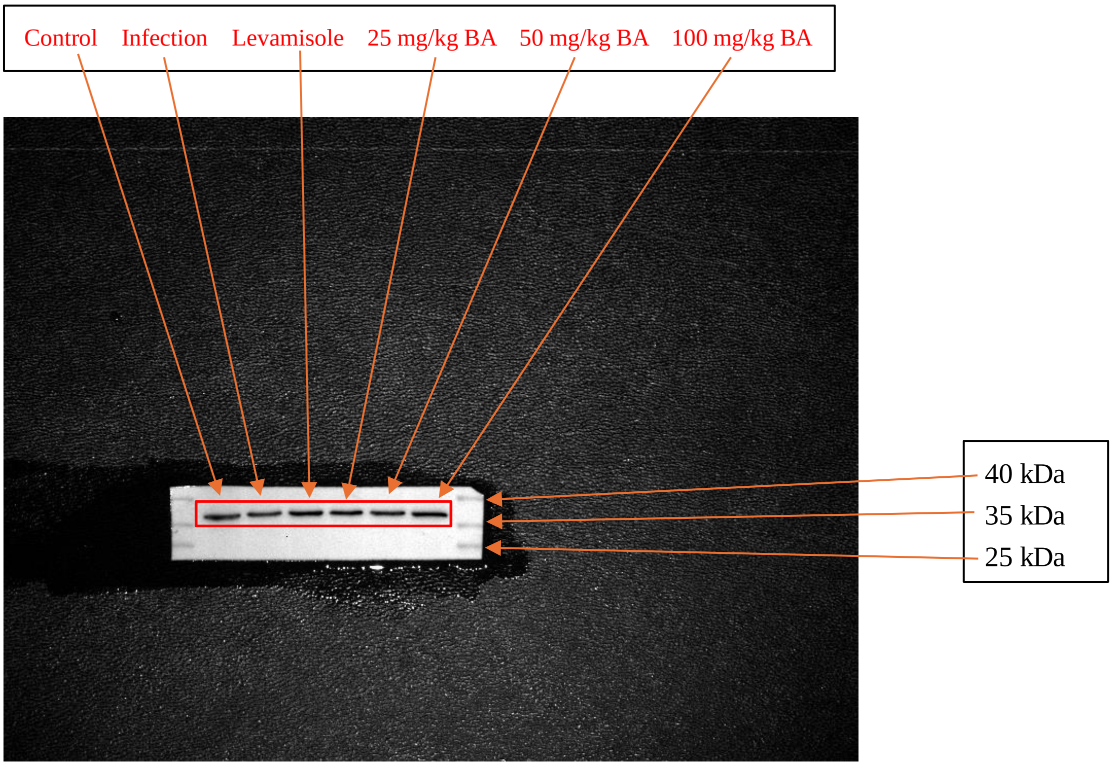

Fig S3

Fig S3. E:

Akt (repeat 1)

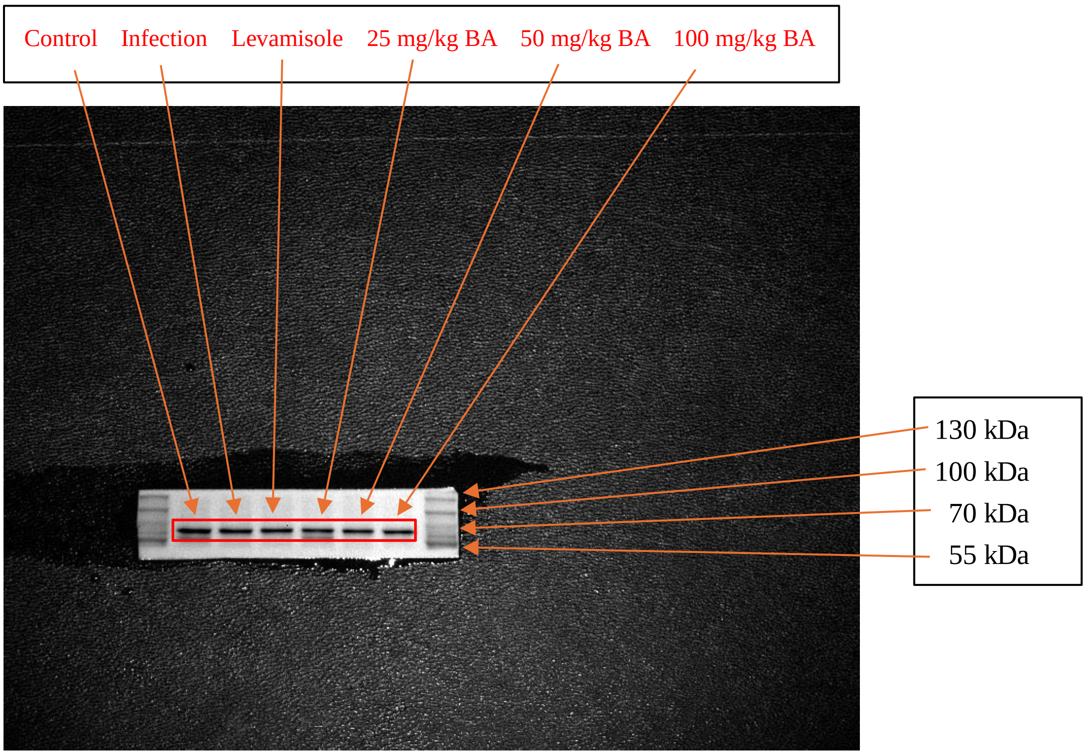

Fig S3

Fig S3. E:

Akt (repeat 2)

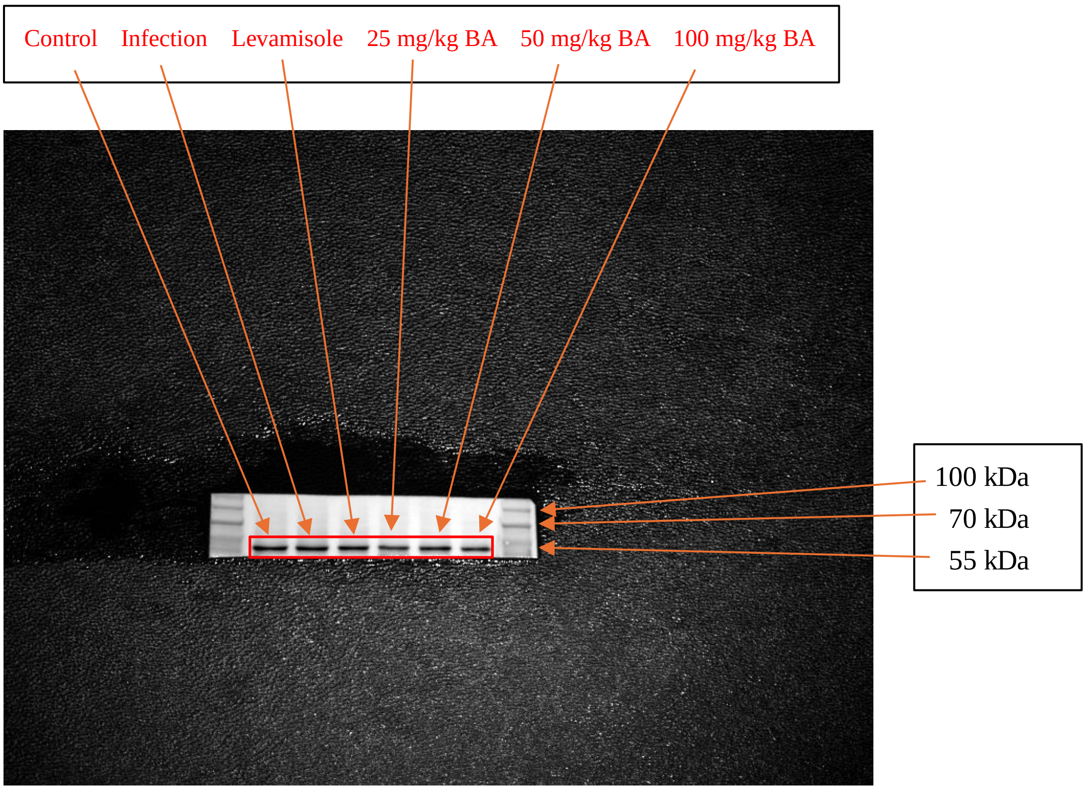

Fig S3

Fig S3. E:

Akt (repeat 3)

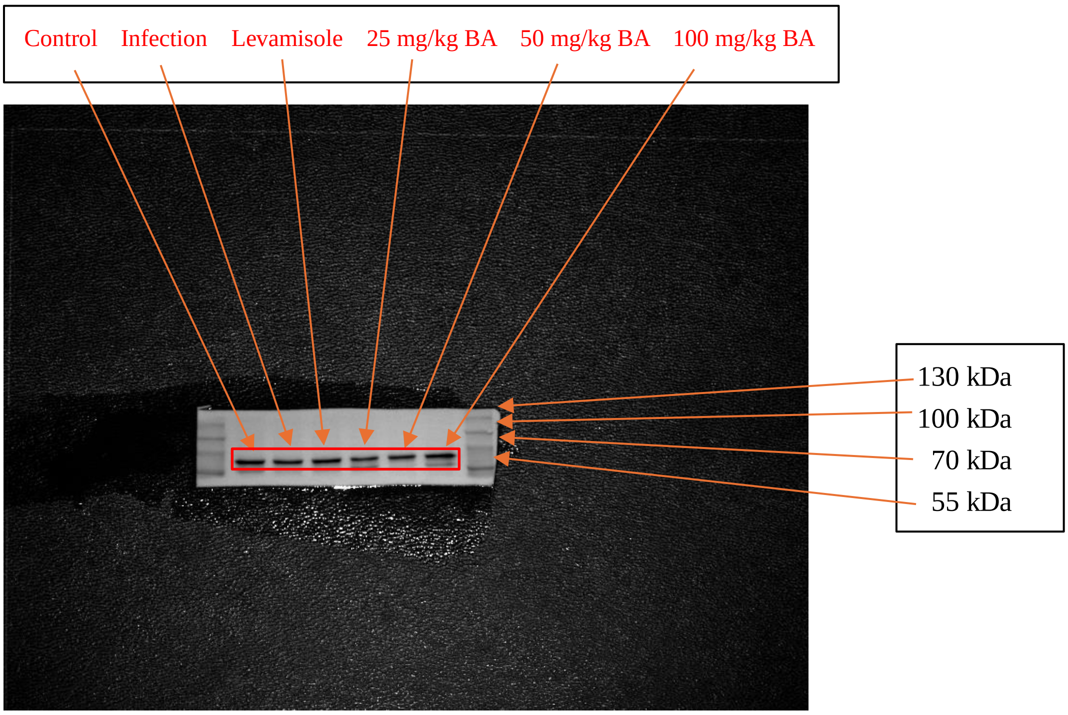

Fig S3

Fig S3. E:

p-Akt (repeat 1)

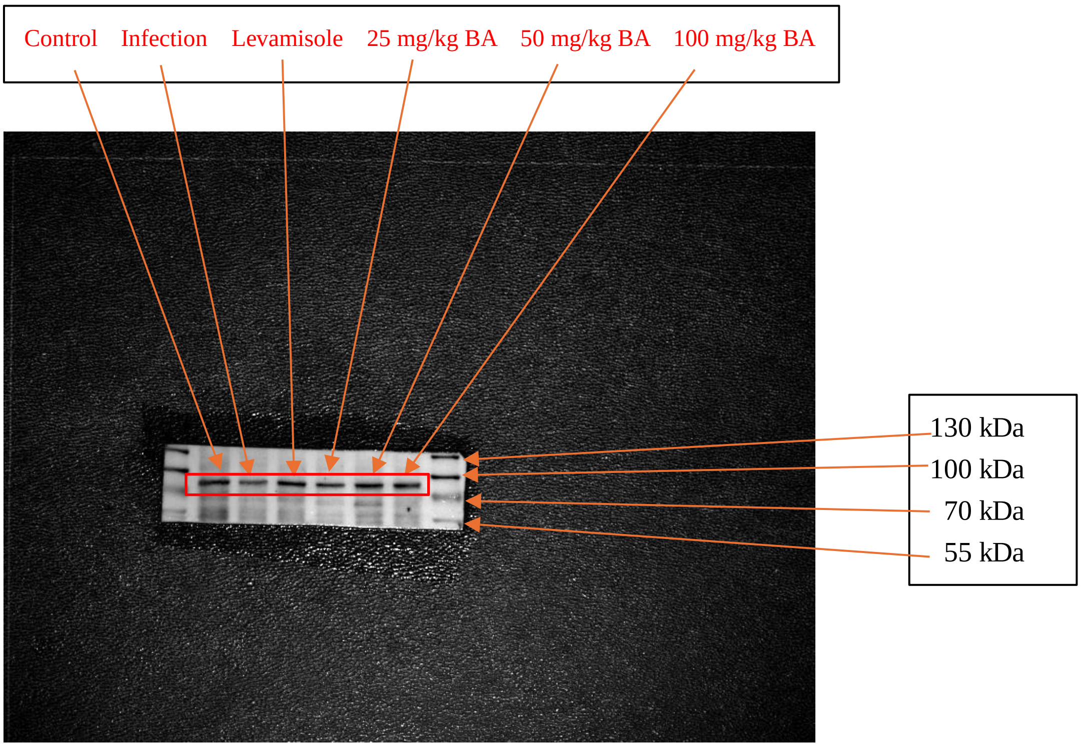

Fig S3

Fig S3. E:

p-Akt (repeat 2)

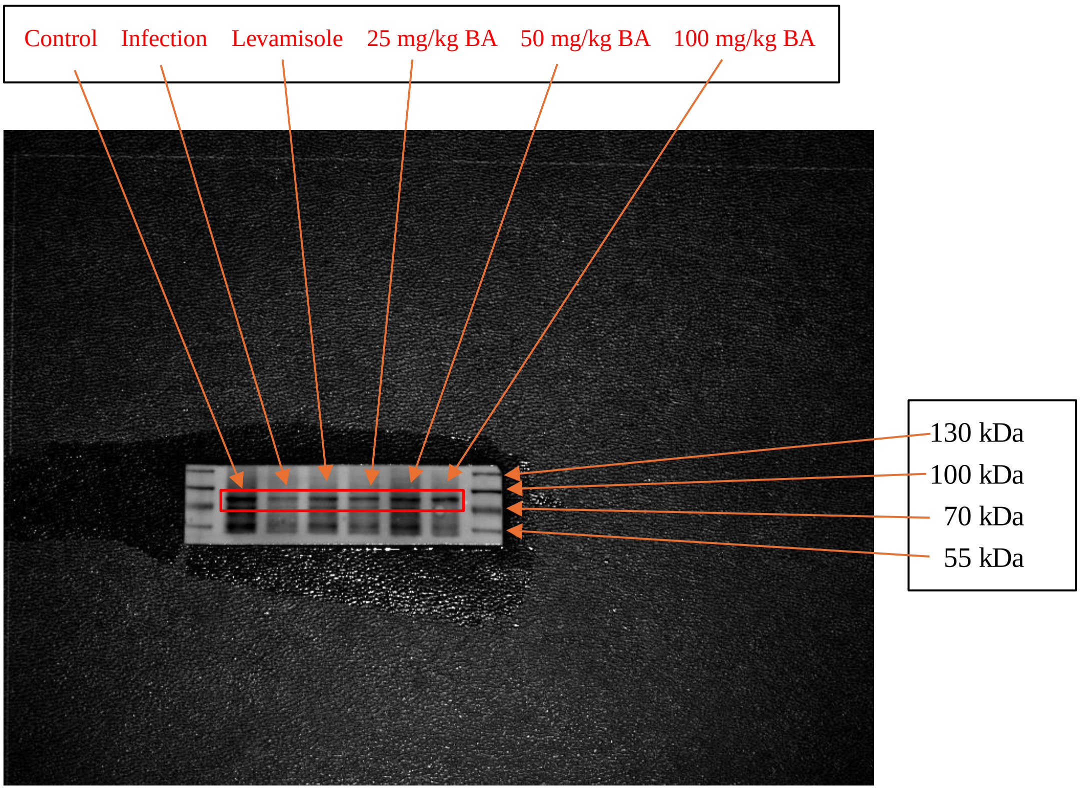

Fig S3

Fig S3. E:

p-Akt (repeat 3)

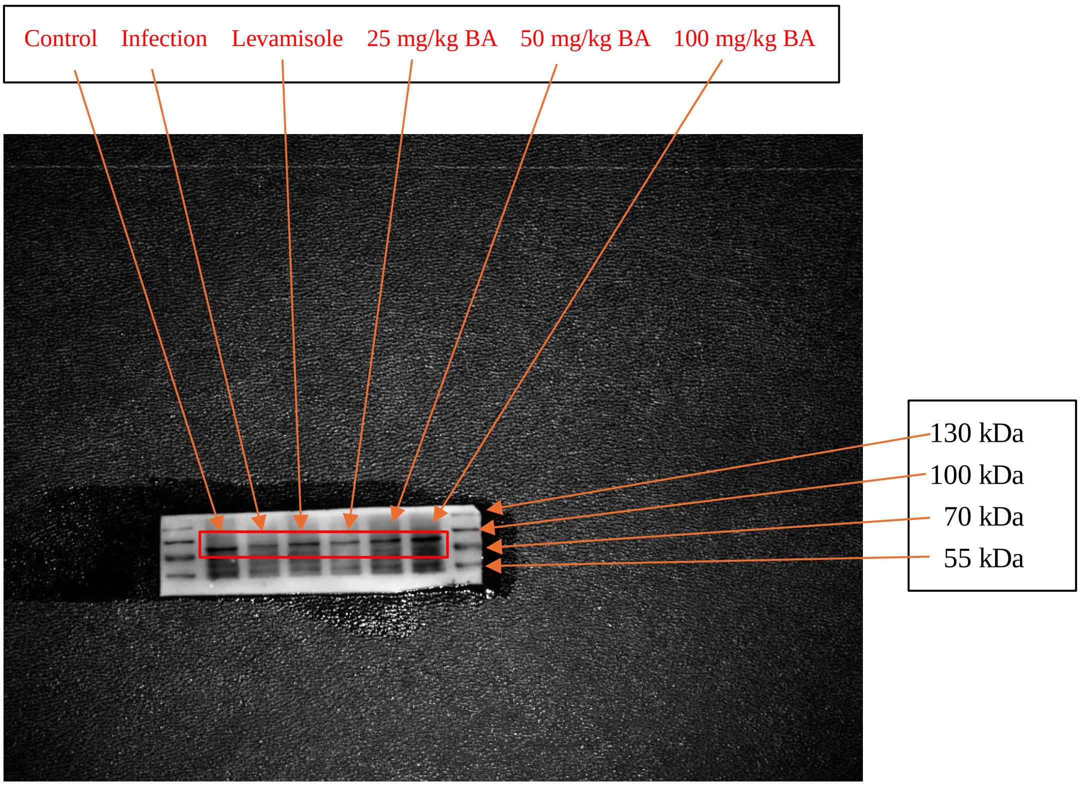

Fig S3

Fig S3. H:

GAPDH (repeat 1)

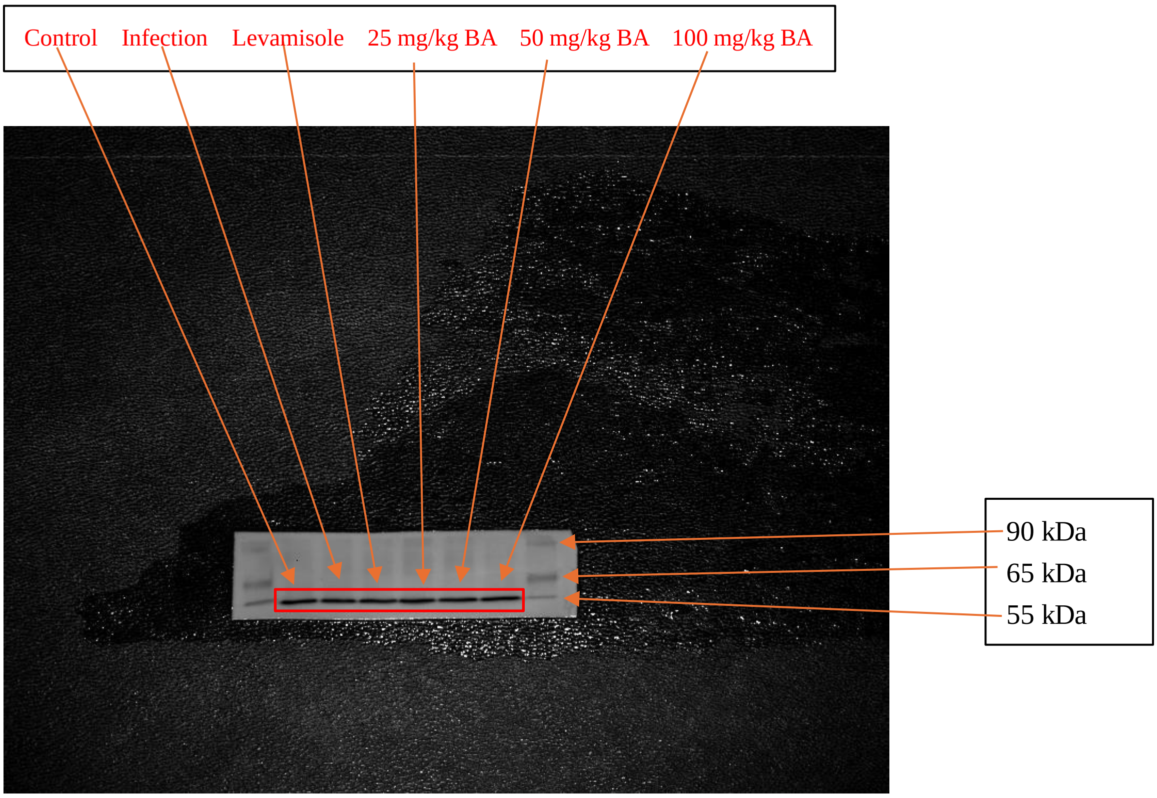

Fig S3

Fig S3. H:

GAPDH (repeat 2)

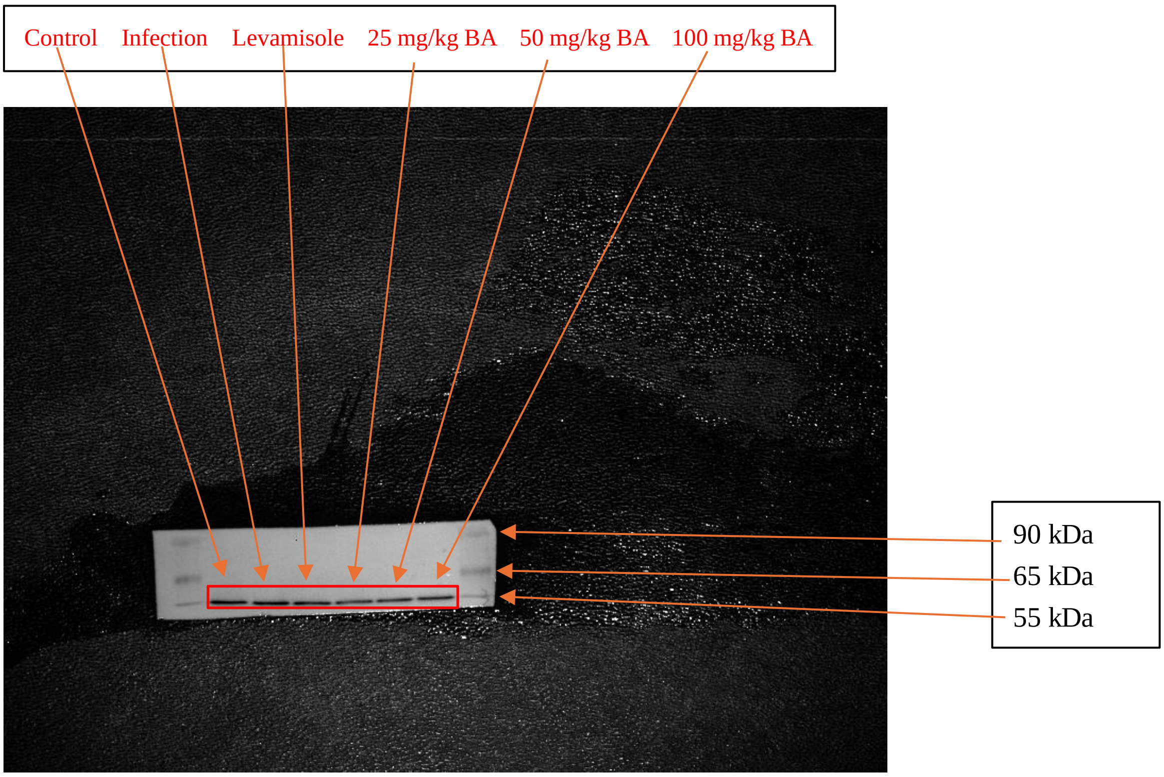

Fig S3

Fig S3. H:

GAPDH (repeat 1)

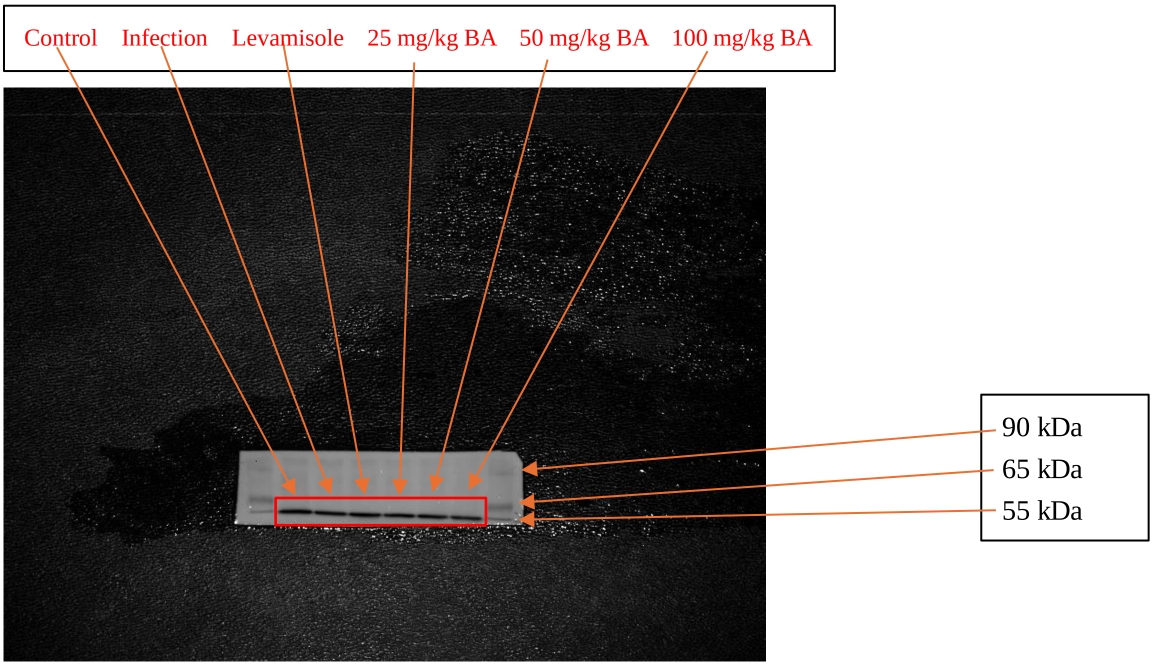

Fig S3

Fig S3. H:

mTOR (repeat 1)

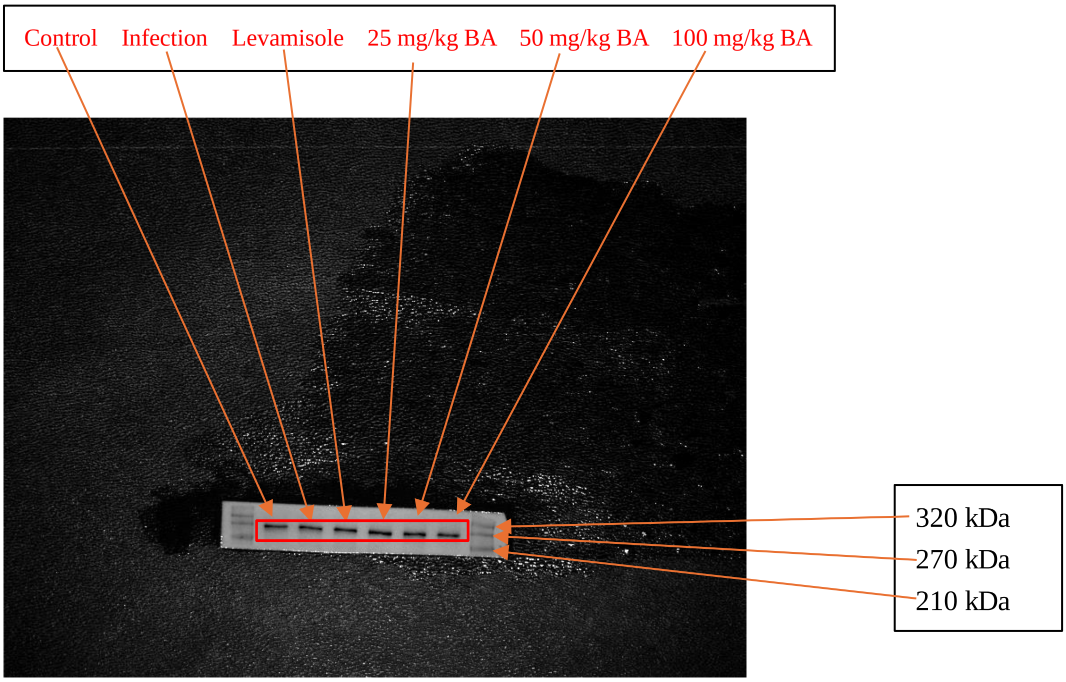

Fig S3

Fig S3. H:

mTOR (repeat 2)

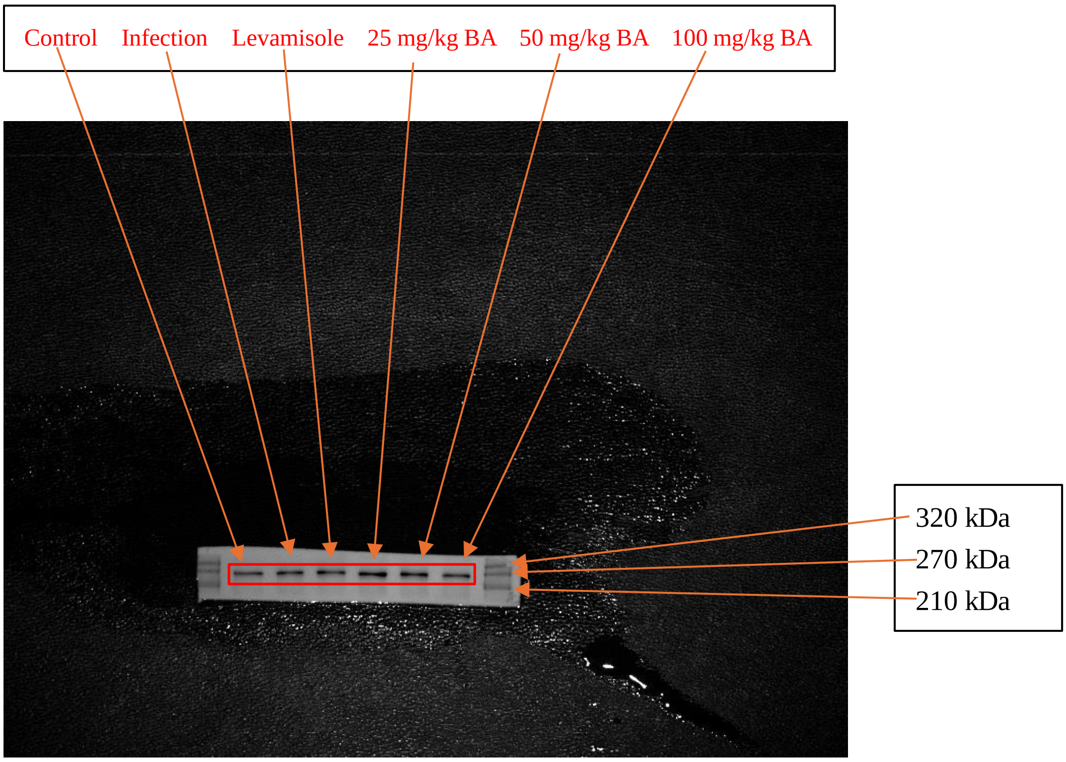

Fig S3

Fig S3. H:

mTOR (repeat 3)

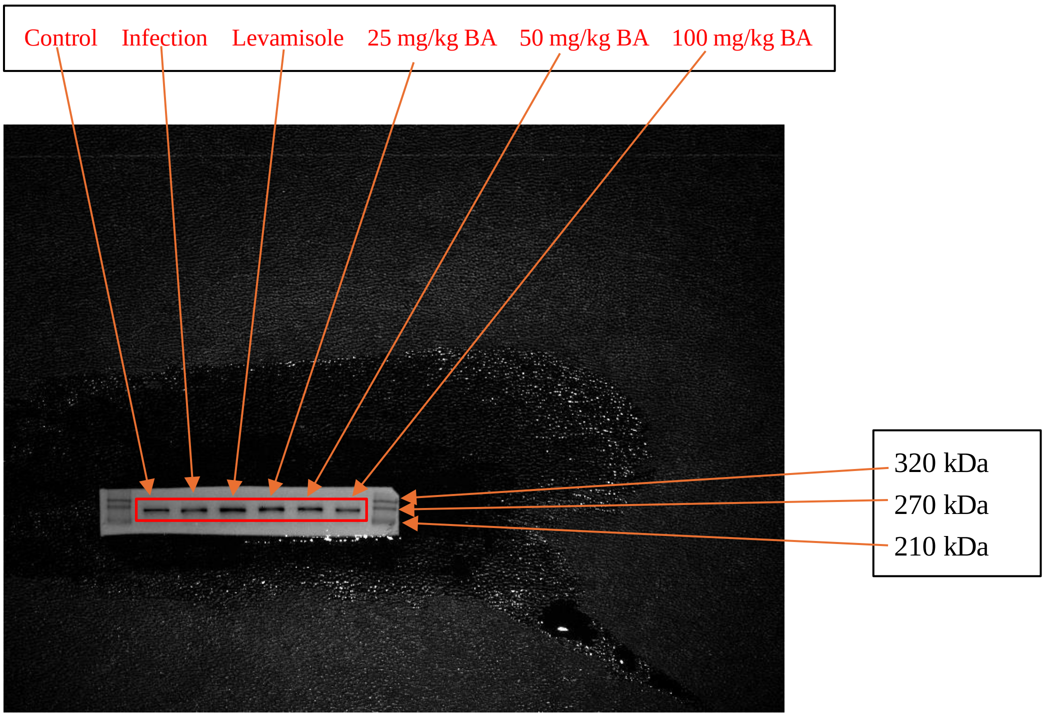

Fig S3

Fig S3. H:

p-mTOR (repeat 1)

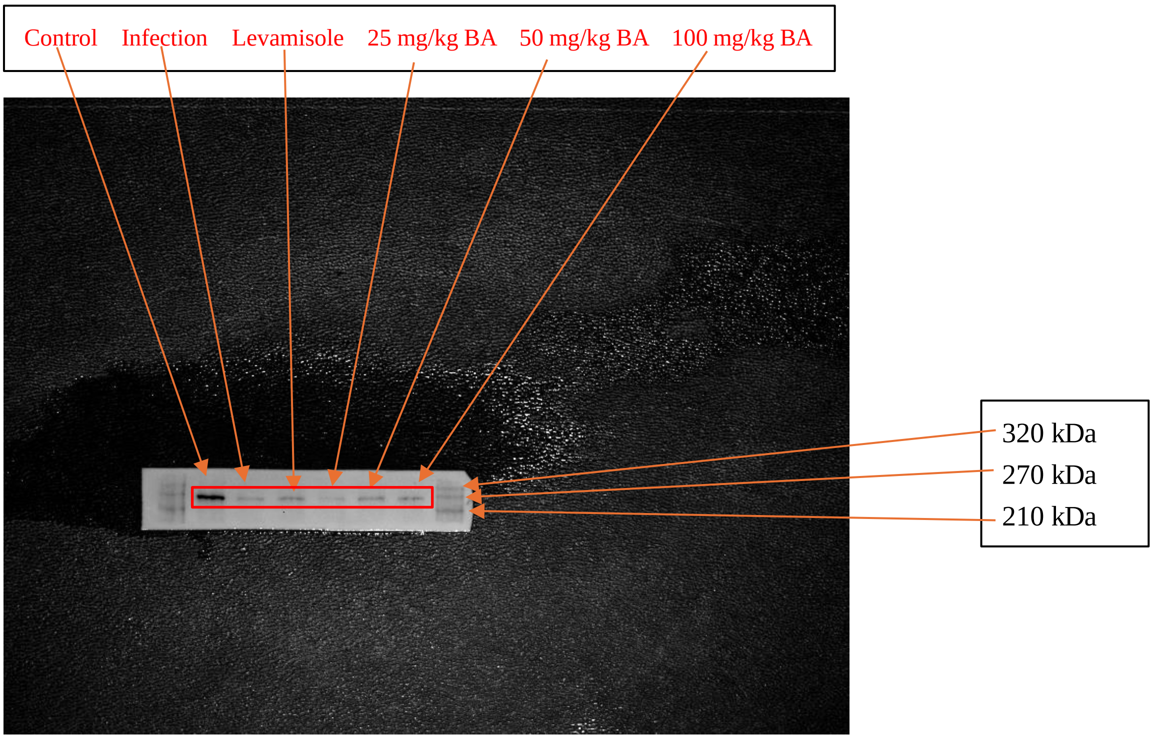

Fig S3

Fig S3. H:

p-mTOR (repeat 2)

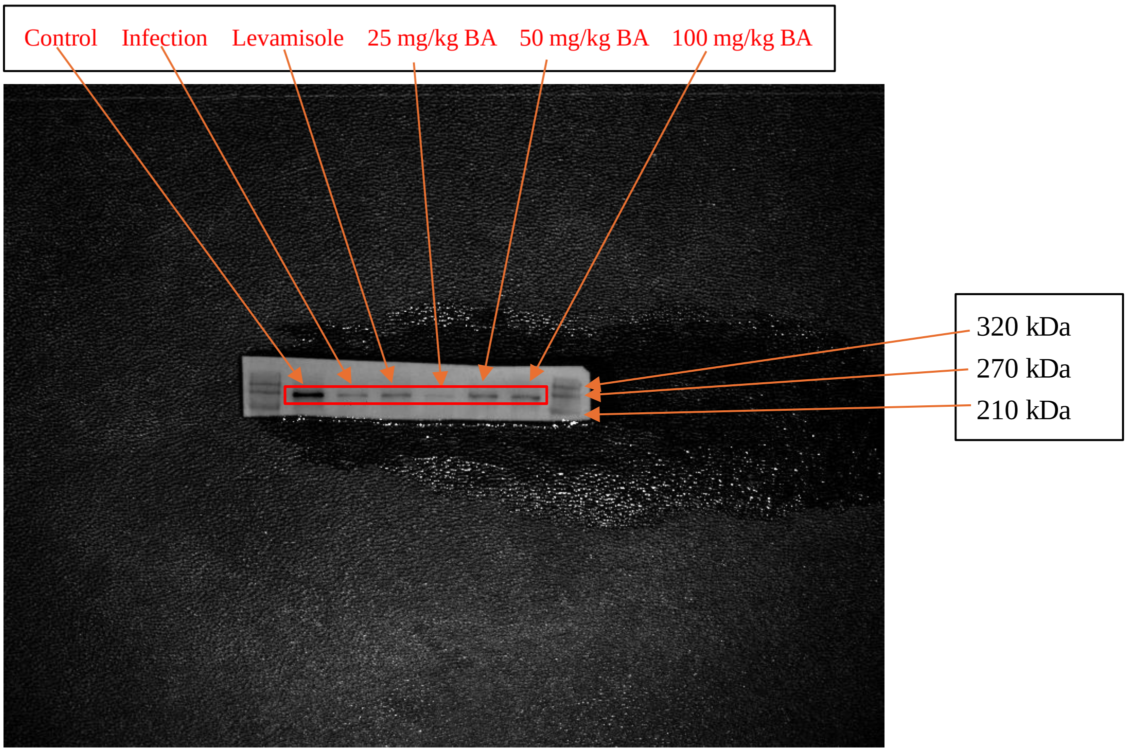

Fig S3

Fig S3. H:

p-mTOR (repeat 3)

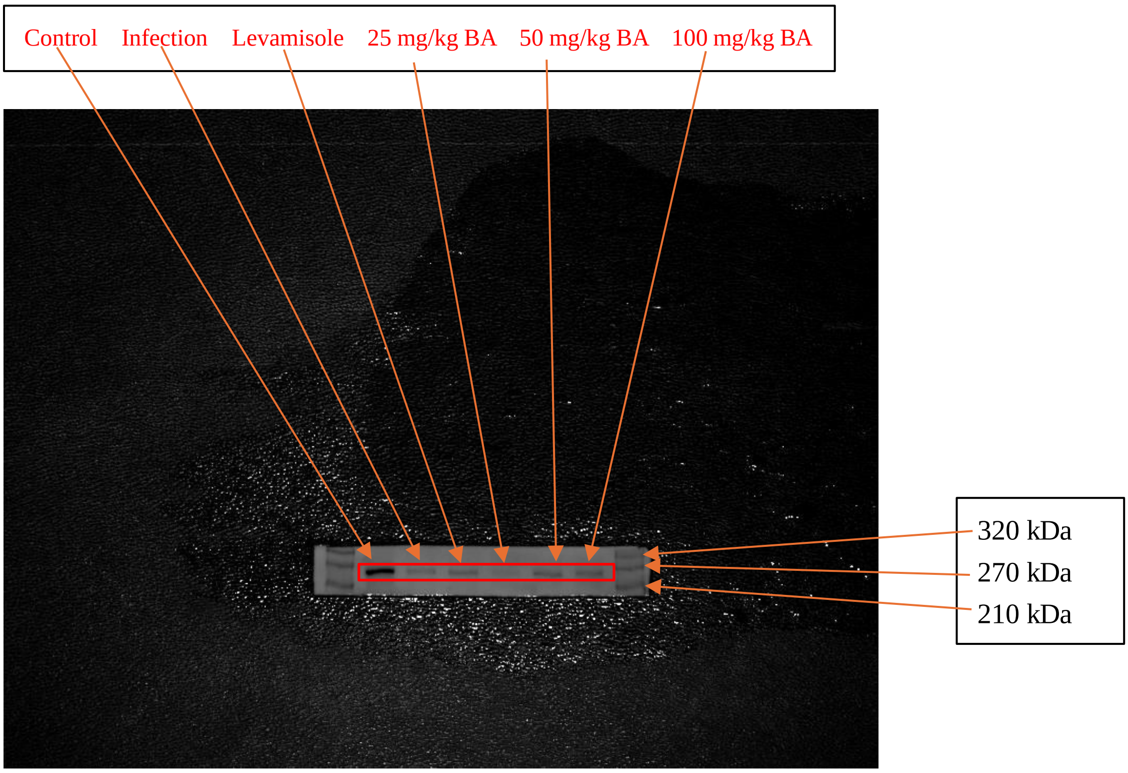

Fig S4

Fig S4. B:

GAPDH (repeat 1)

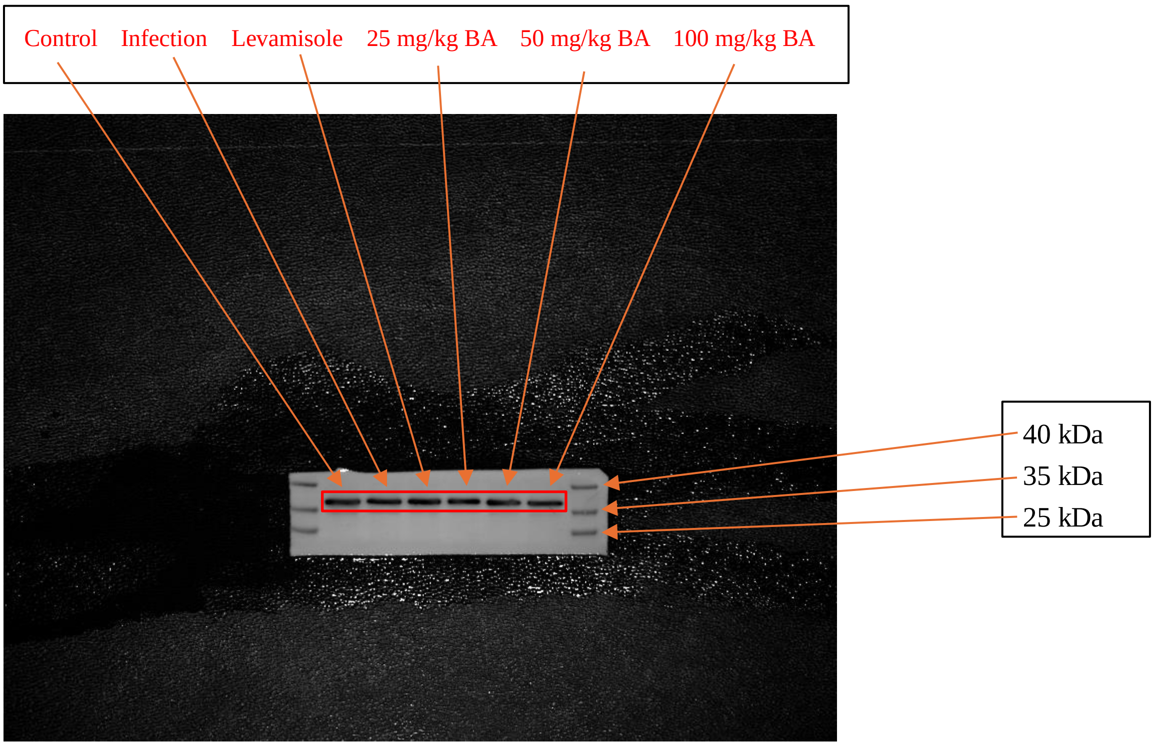

Fig S4

Fig S4. B:

GAPDH (repeat 2)

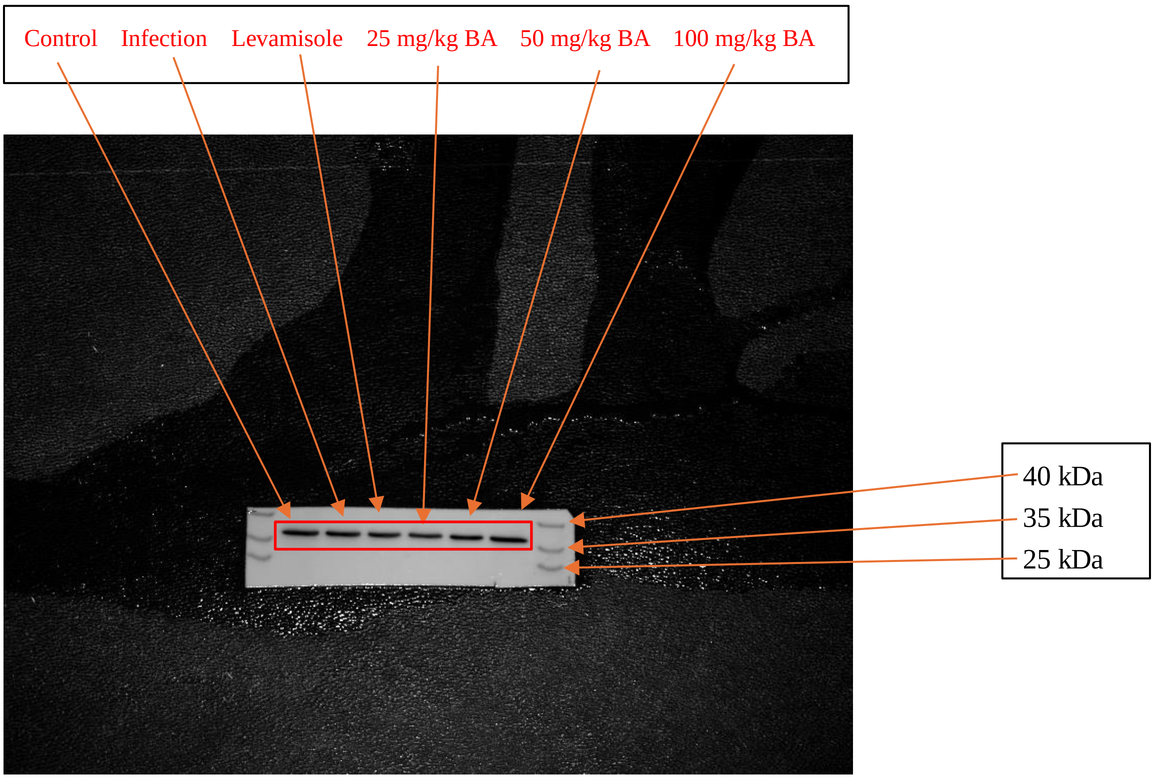

Fig S4

Fig S4. B:

GAPDH (repeat 3)

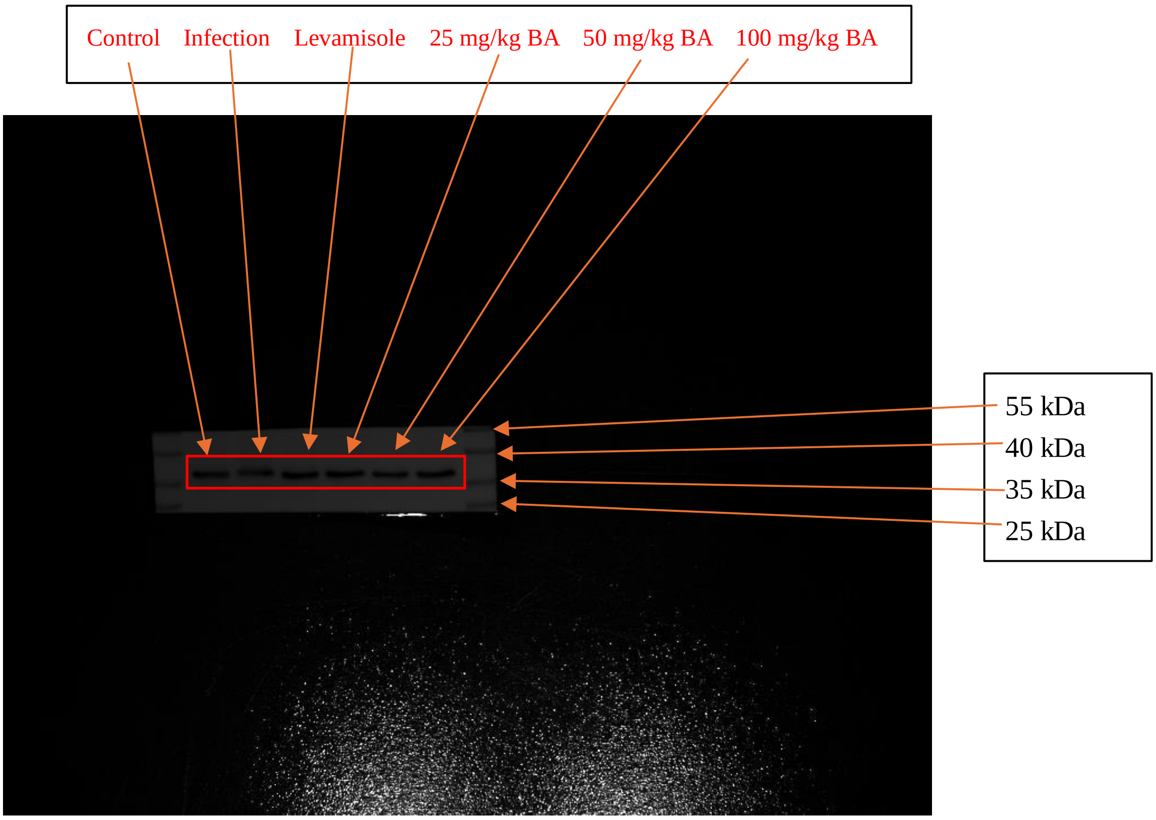

Fig S4

Fig S4. B:

RAF (repeat 1)

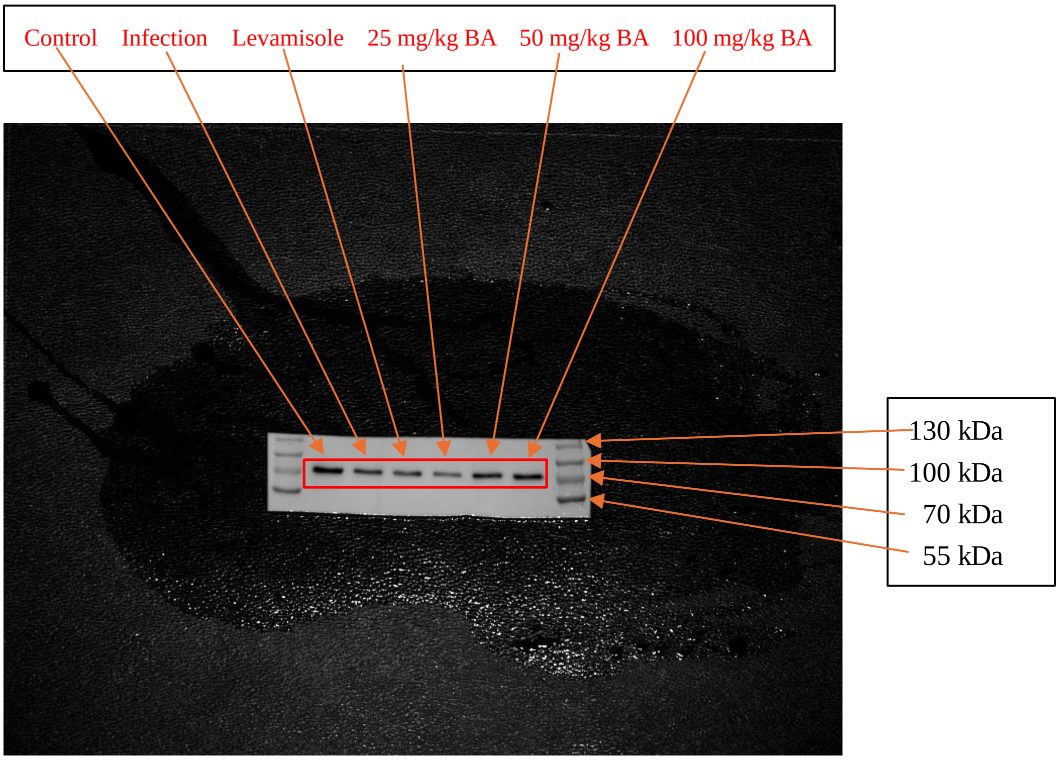

Fig S4

Fig S4. B:

RAF (repeat 2)

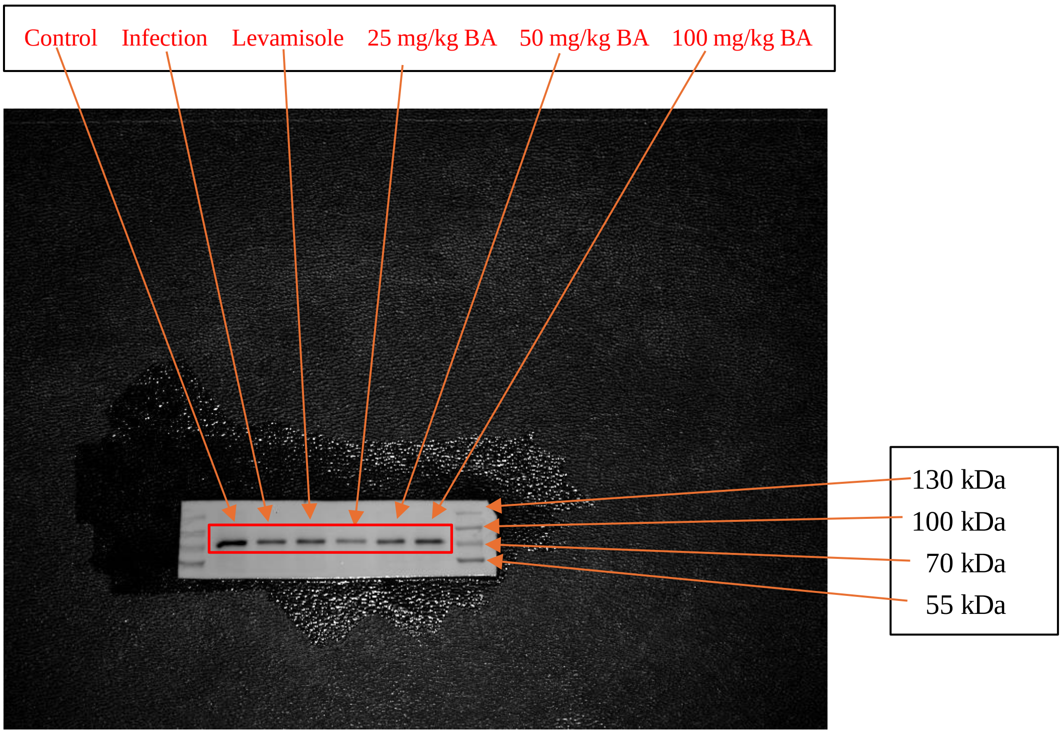

Fig S4

Fig S4. B:

RAF (repeat 3)

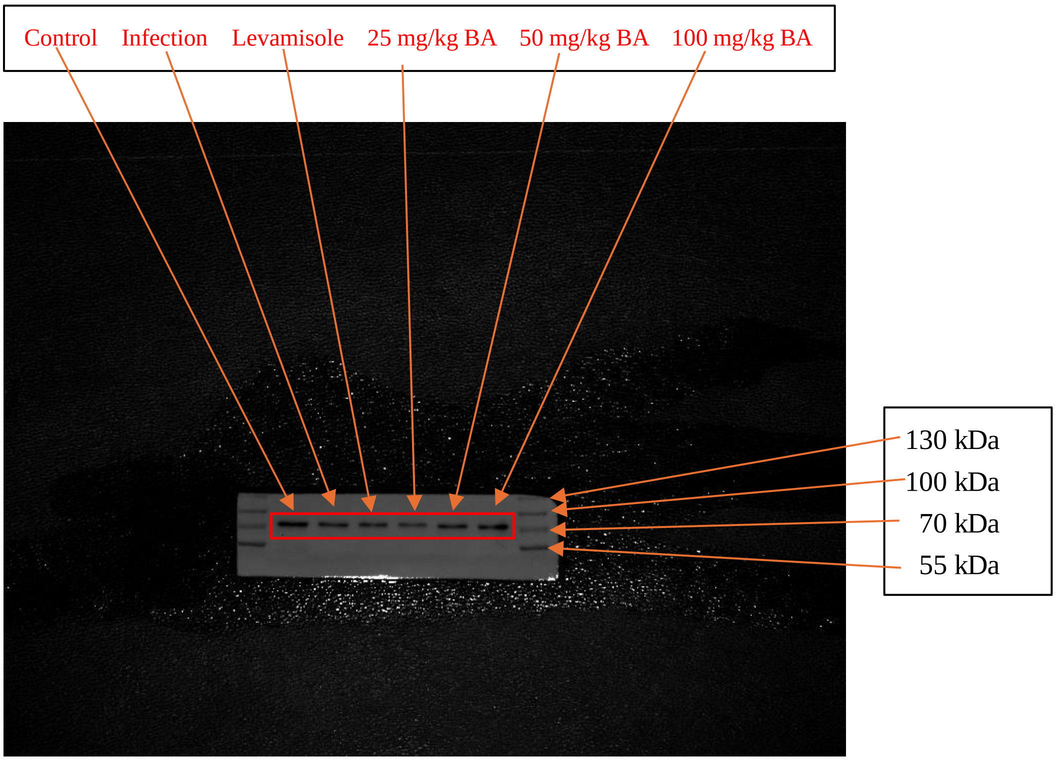

Fig S4

Fig S4. E:

GAPDH (repeat 1)

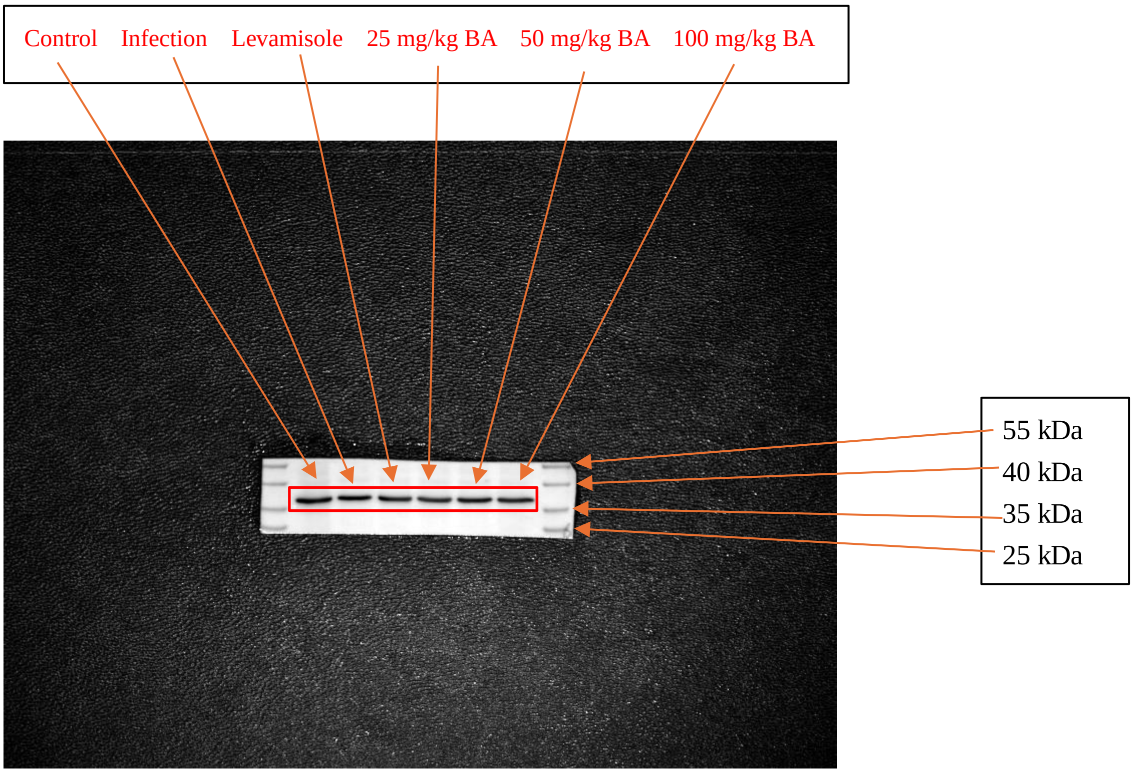

Fig S4

Fig S4. E:

GAPDH (repeat 2)

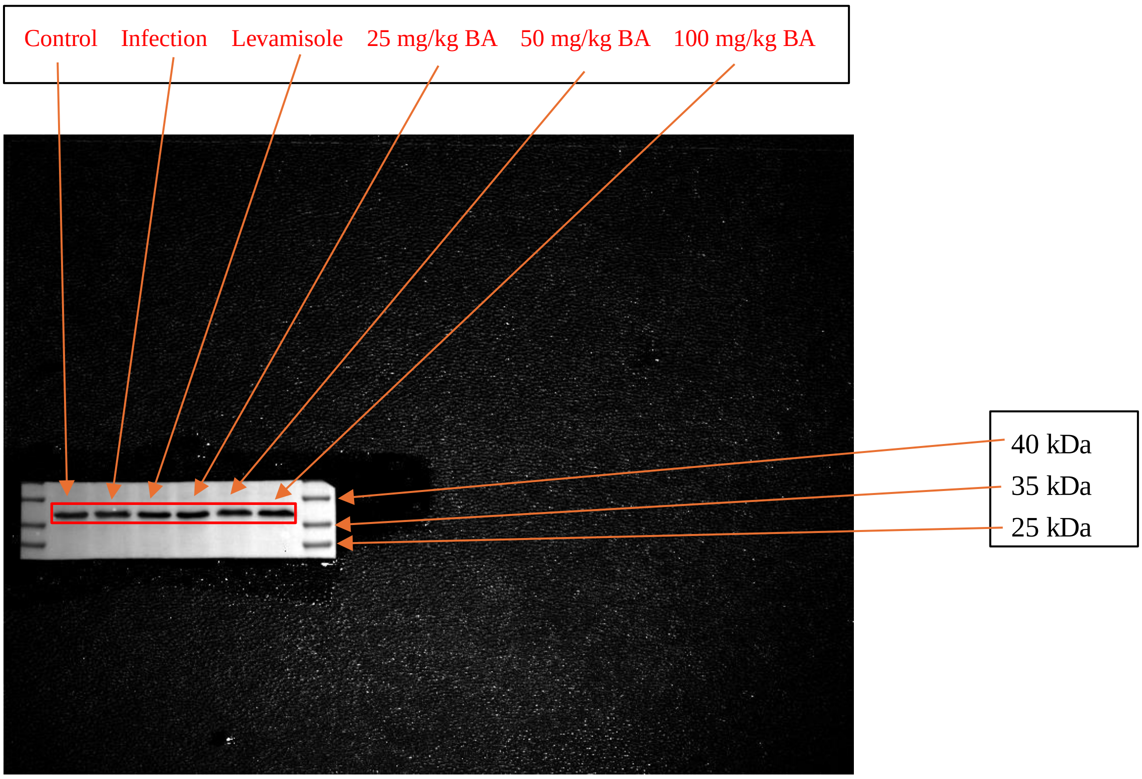

Fig S4

Fig S4. E:

GAPDH (repeat 3)

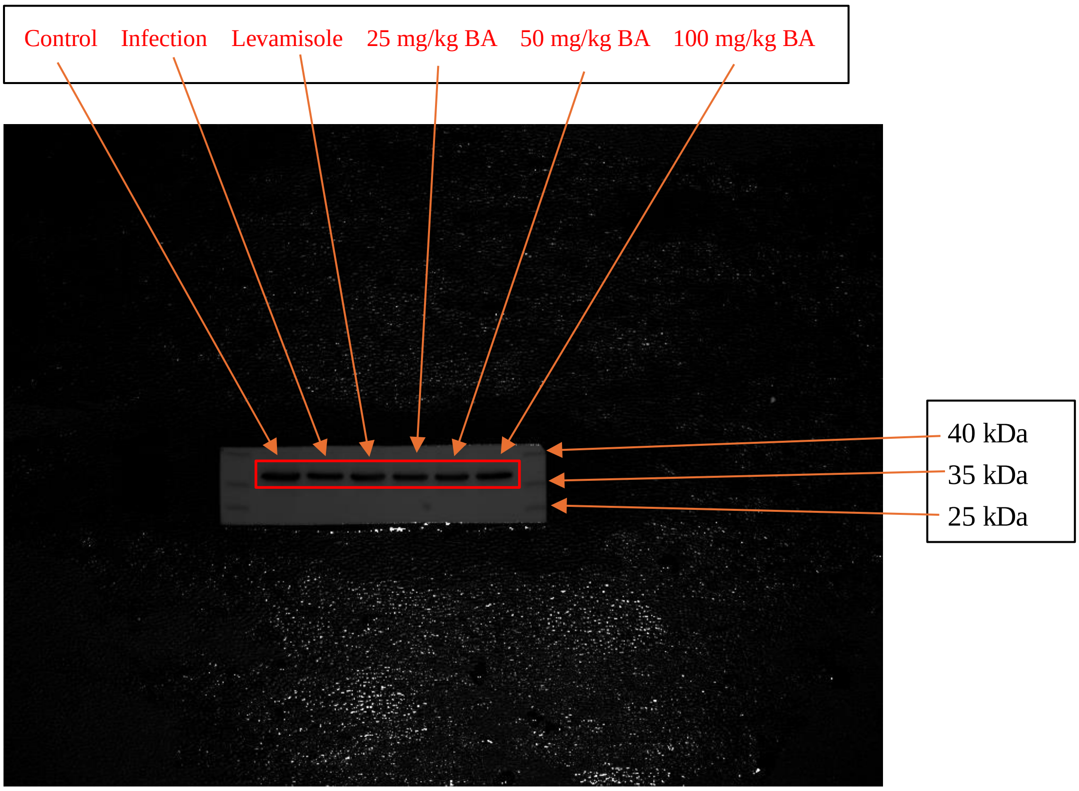

Fig S4

Fig S4. E:

MEK (repeat 1)

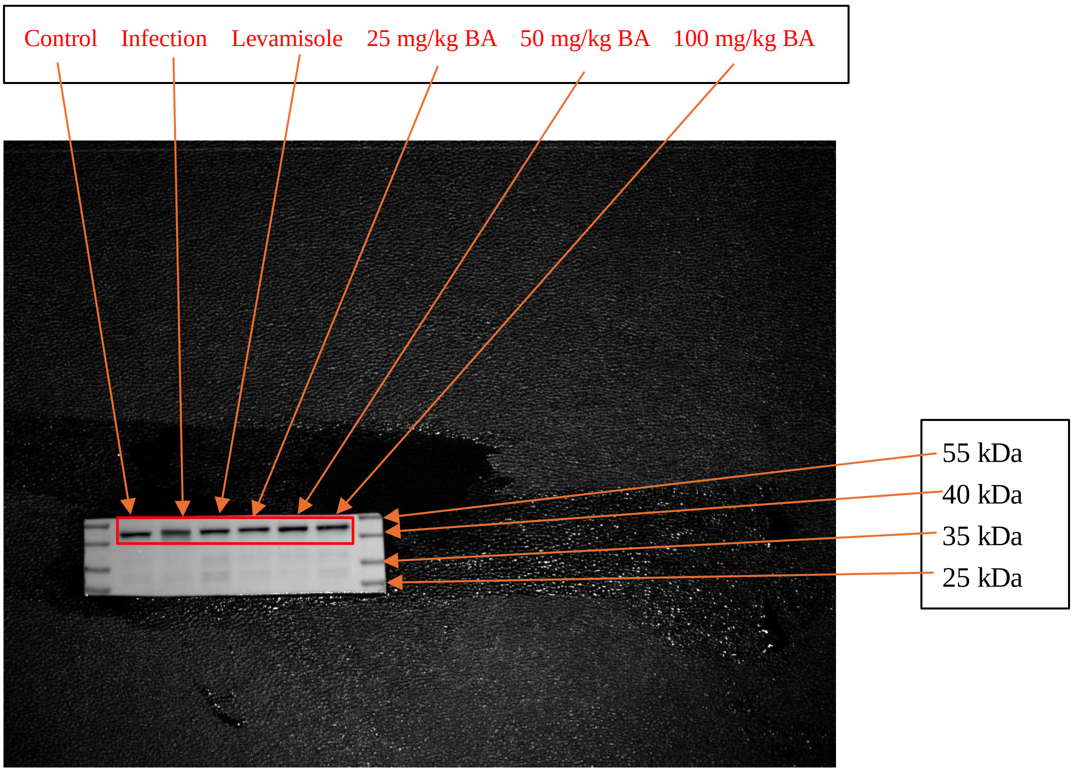

Fig S4

Fig S4. E:

MEK (repeat 2)

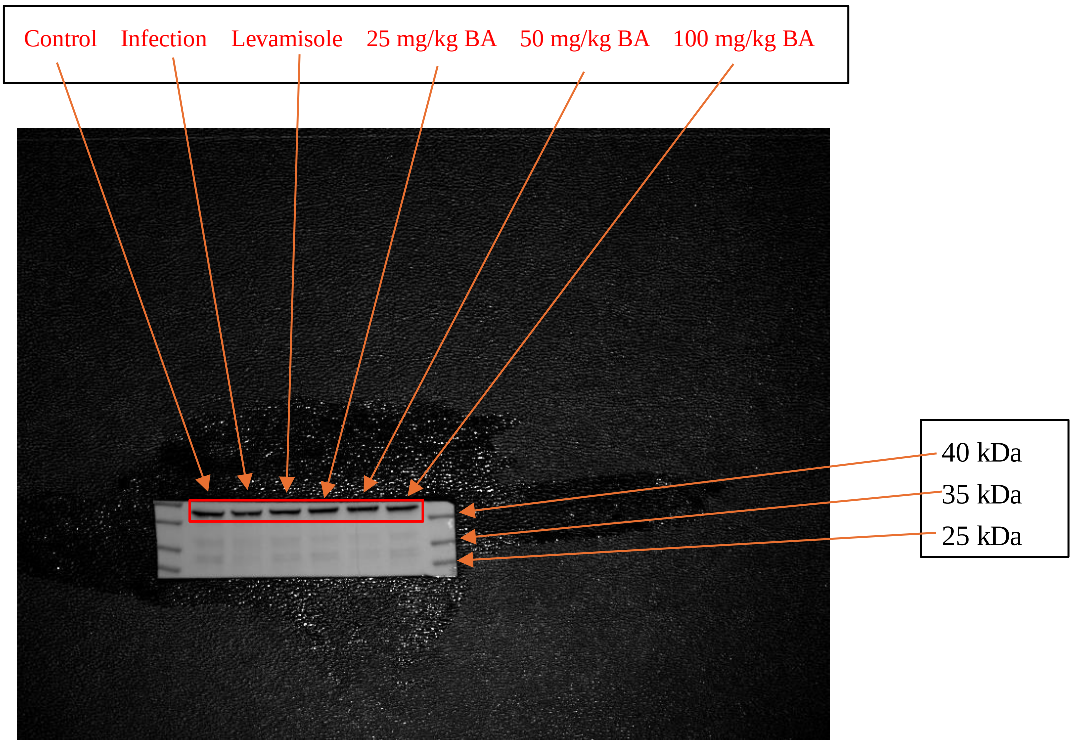

Fig S4

Fig S4. E:

MEK (repeat 3)

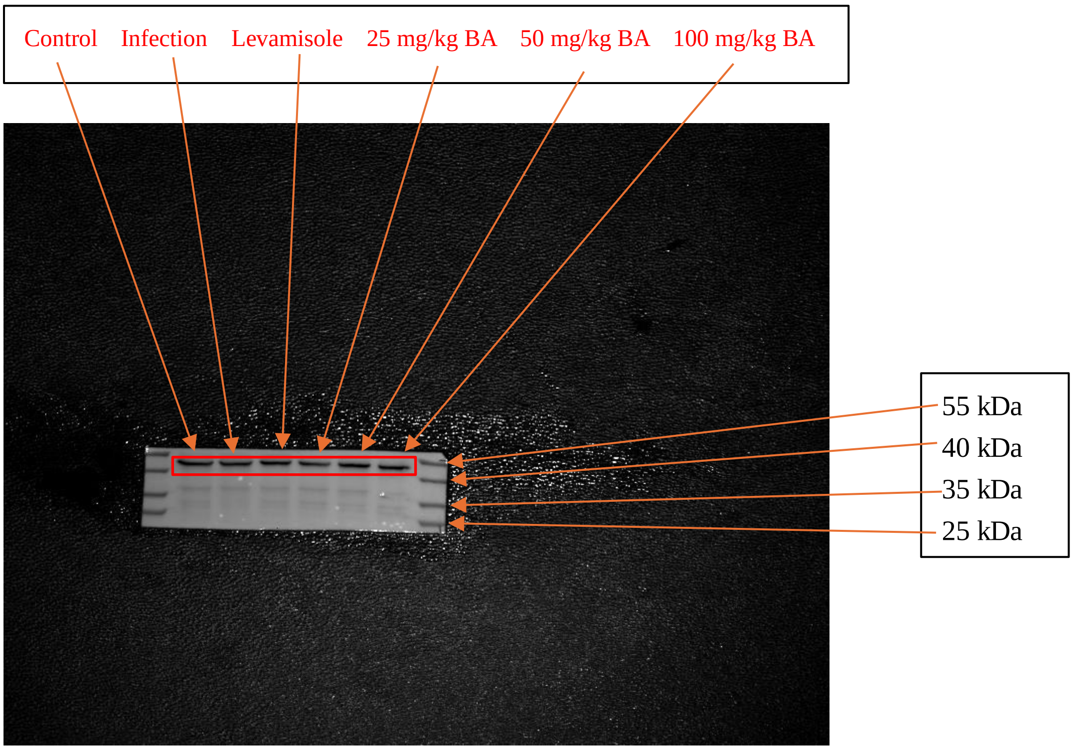

Fig S4

Fig S4. E:

p-MEK1/2 (repeat 1)

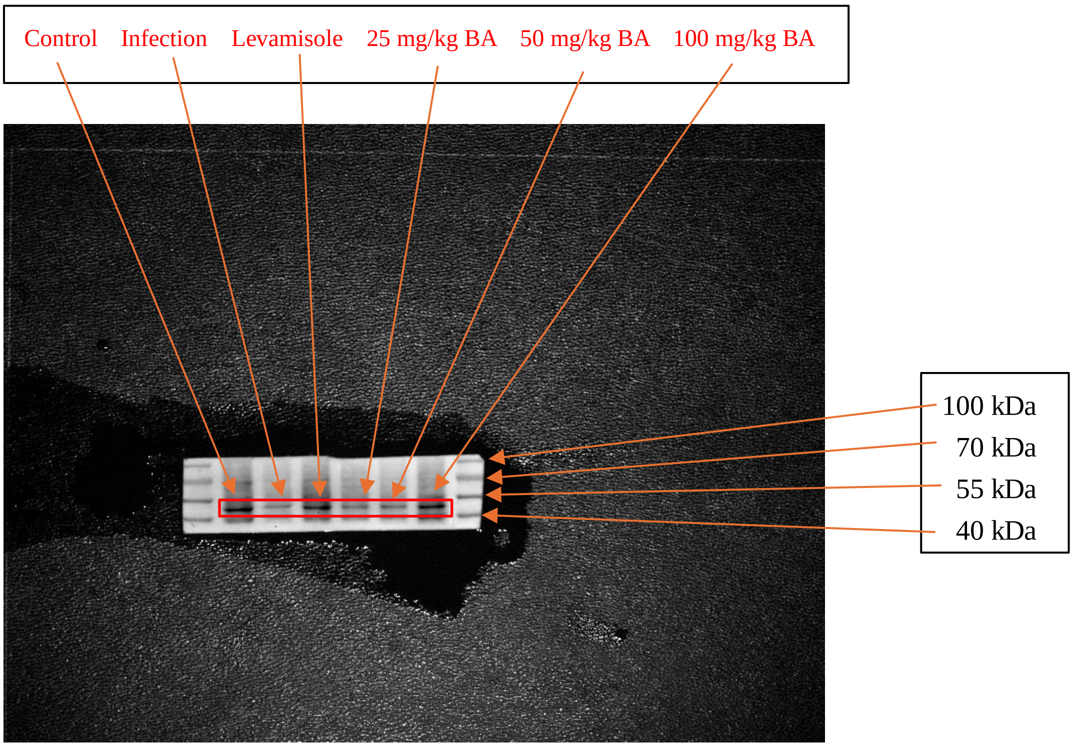

Fig S4

Fig S4. E:

p-MEK1/2 (repeat 2)

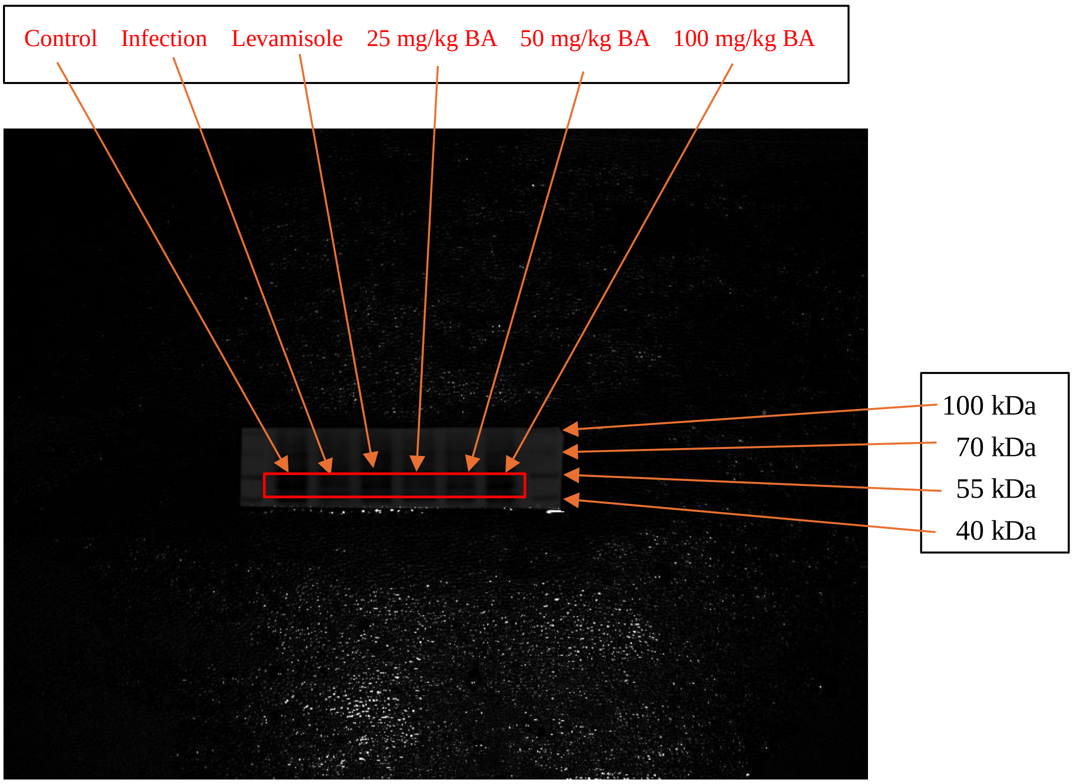

Fig S4

Fig S4. E:

p-MEK1/2 (repeat 3)

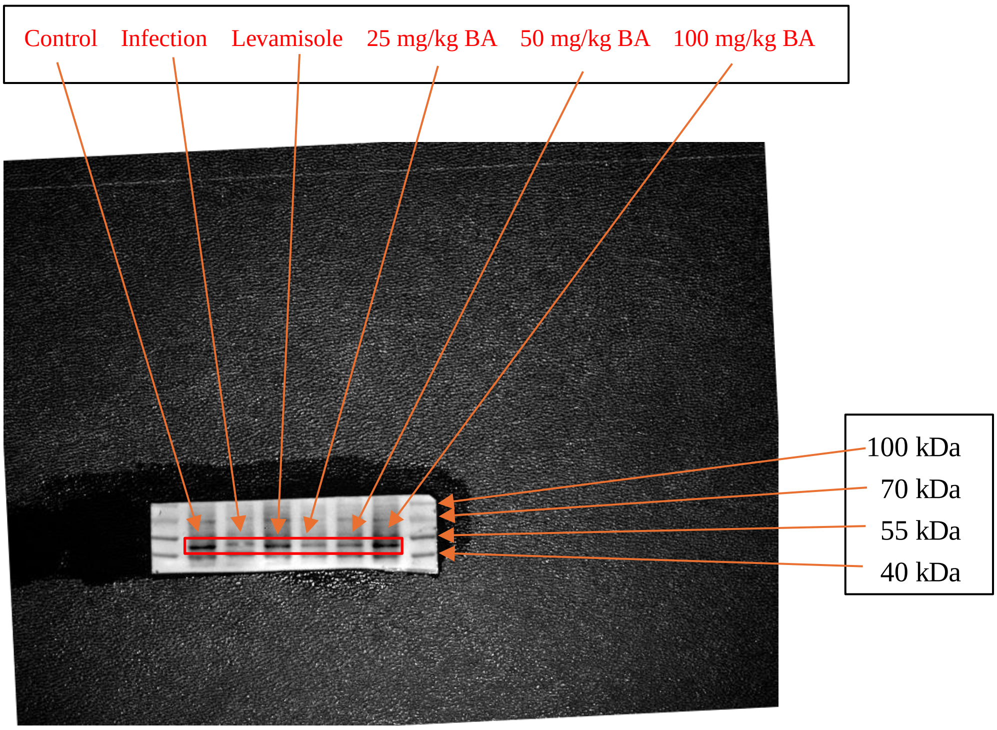

Fig S4

Fig S4. H:

GAPDH (repeat 1)

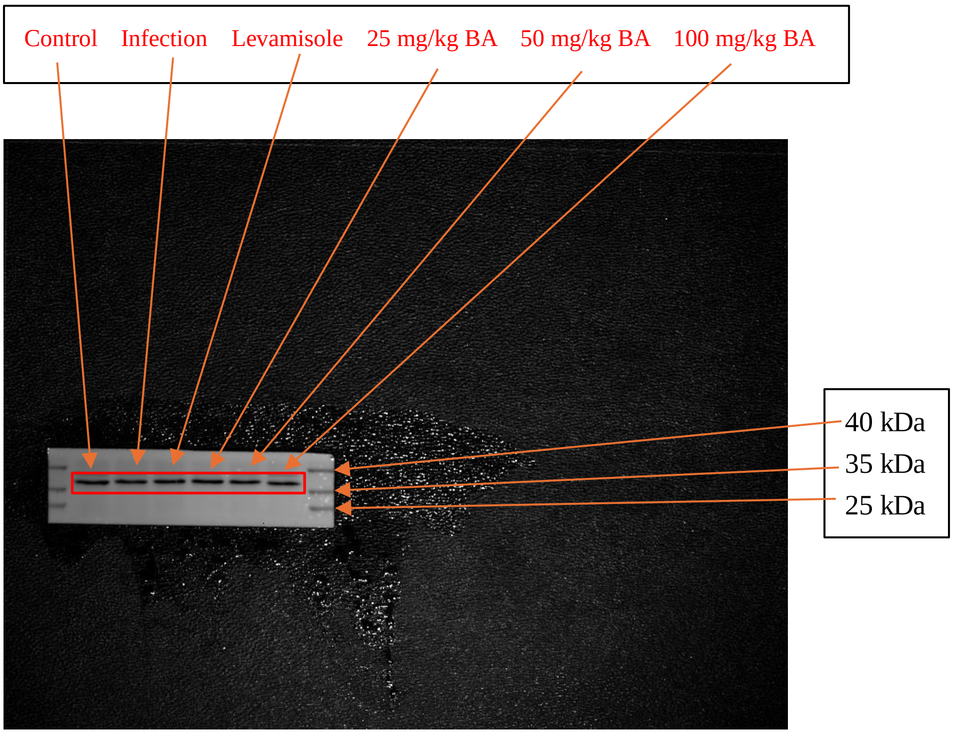

Fig S4

Fig S4. H:

GAPDH (repeat 2)

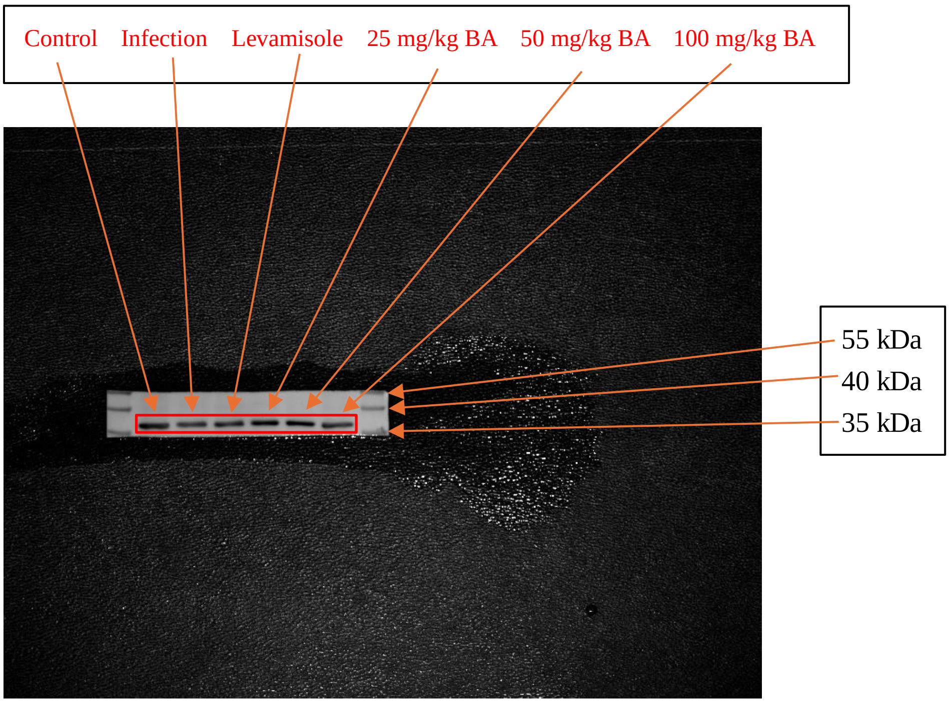

Fig S4

Fig S4. H:

GAPDH (repeat 3)

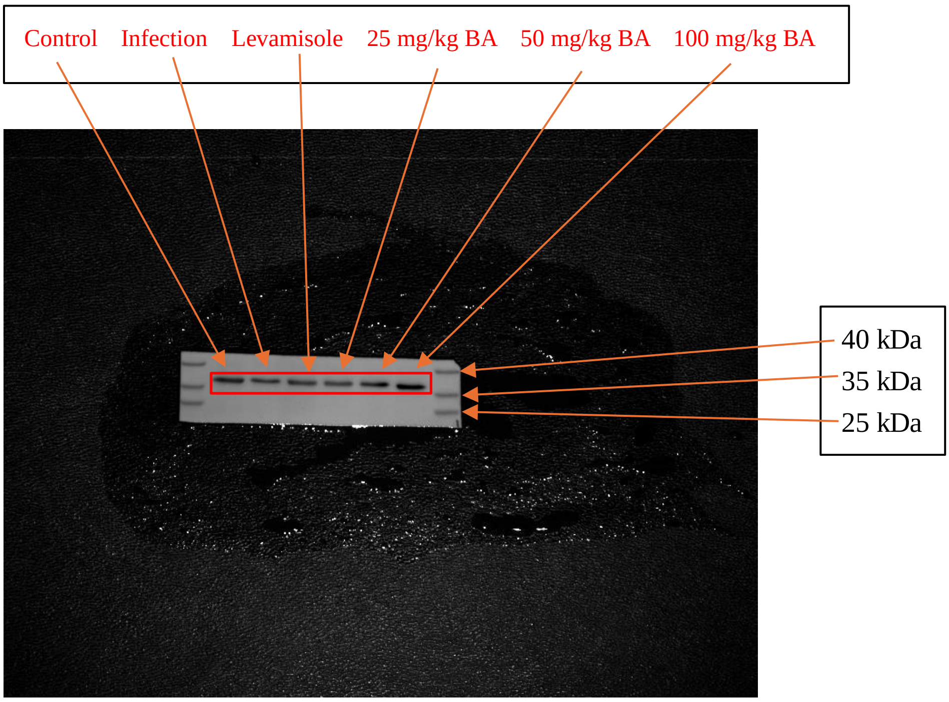

Fig S4

Fig S4. H:

ERK (repeat 1)

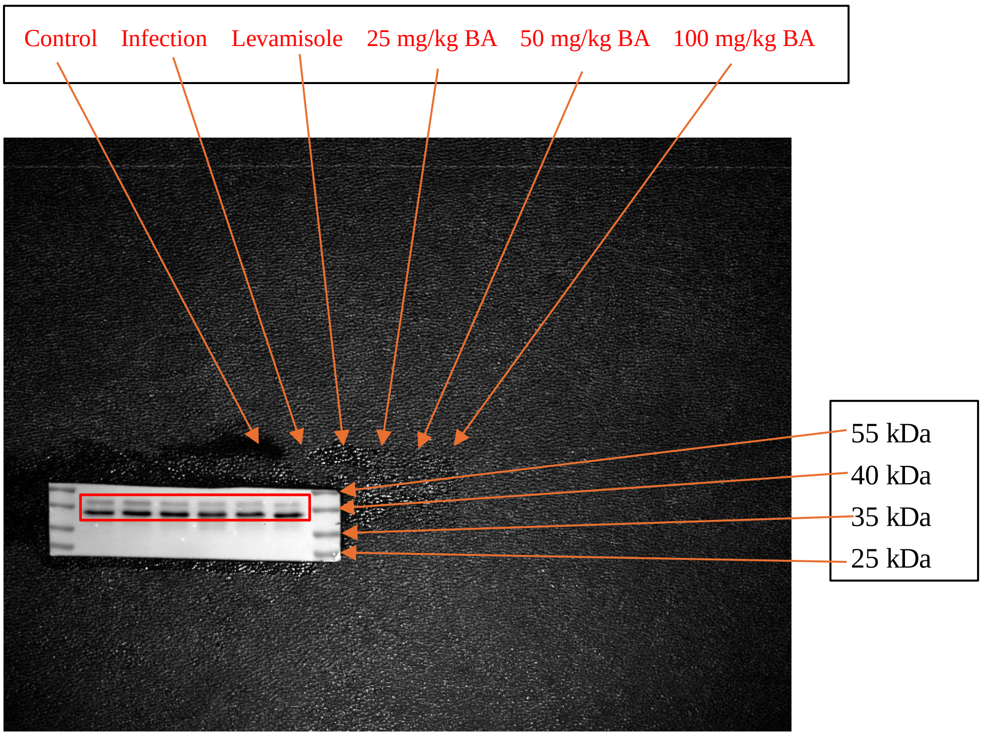

Fig S4

Fig S4. H:

ERK (repeat 2)

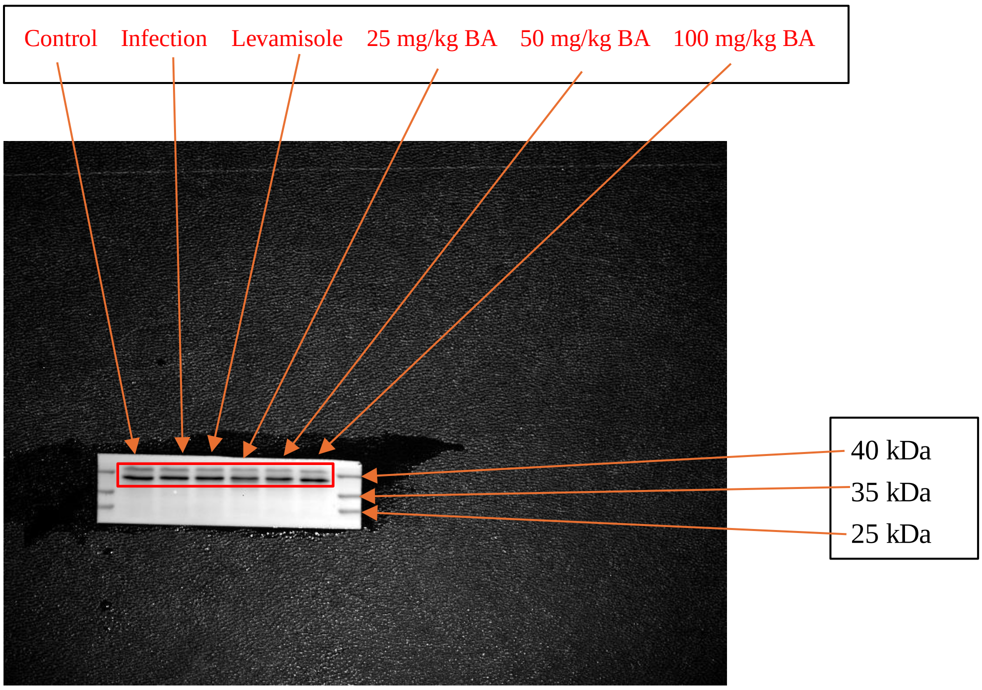

Fig S4

Fig S4. H:

ERK (repeat 3)

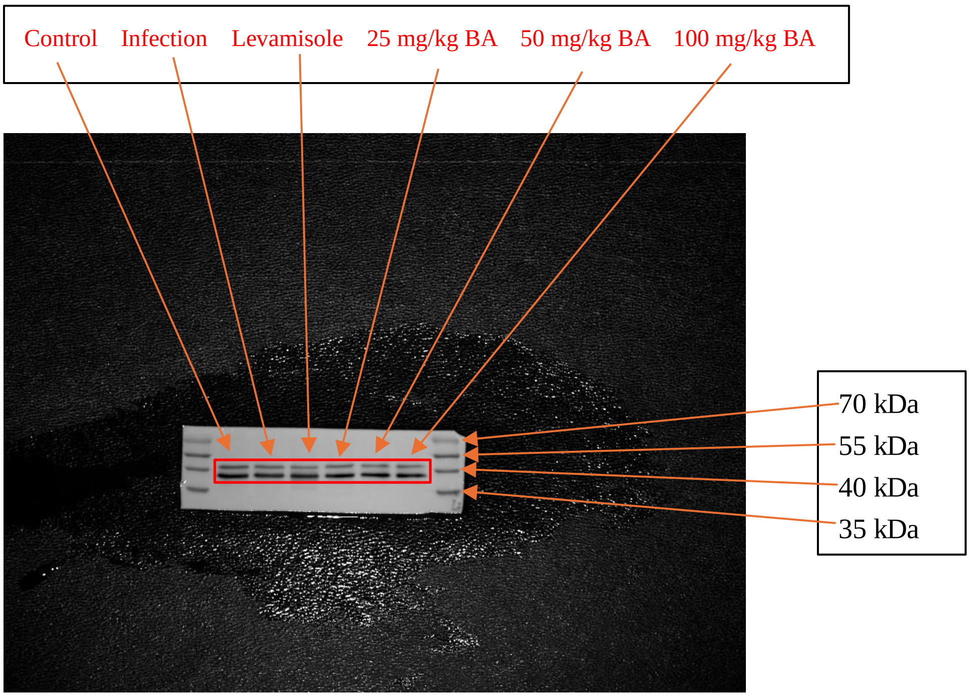

Fig S4

Fig S4. H:

p-ERK1/2 (repeat 1)

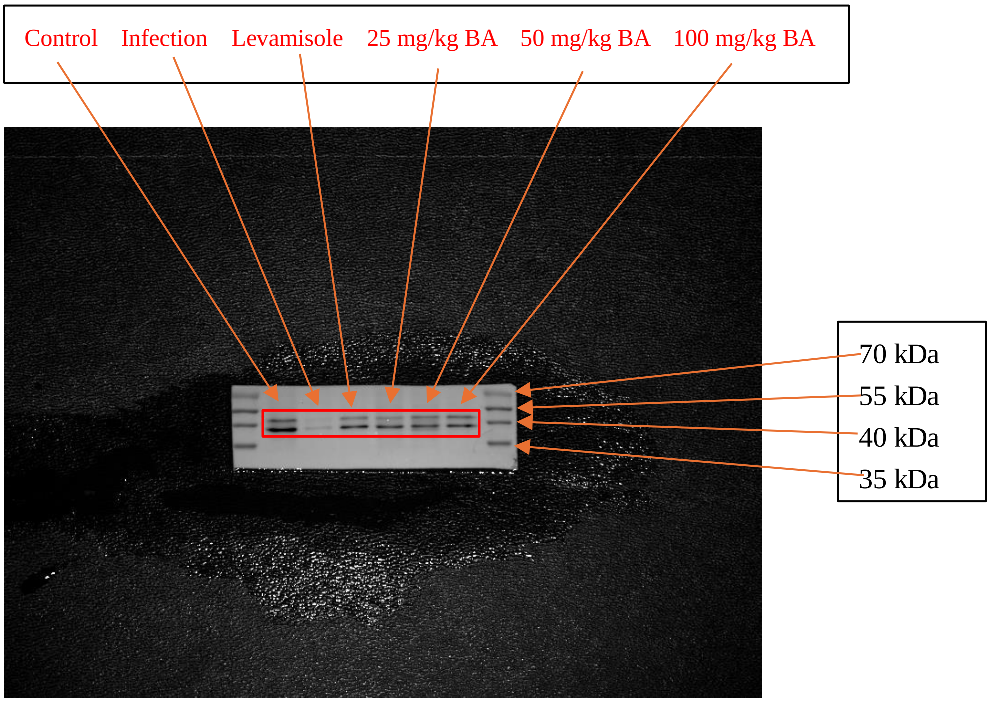

Fig S4

Fig S4. H:

p-ERK1/2 (repeat 2)

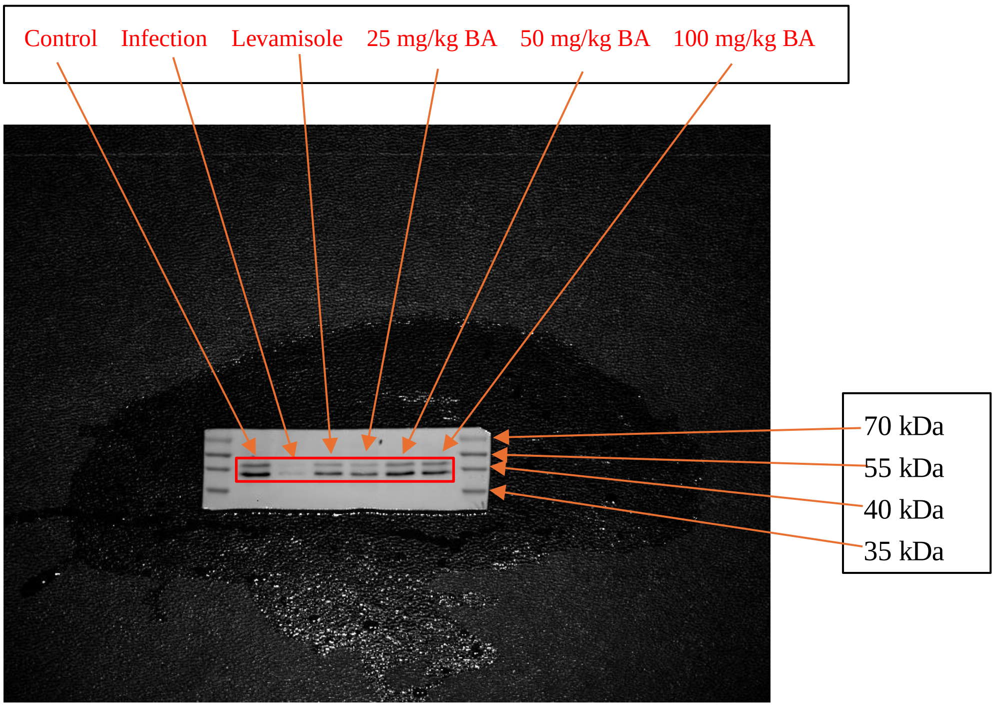

Fig S4

Fig S4. H:

p-ERK1/2 (repeat 3)

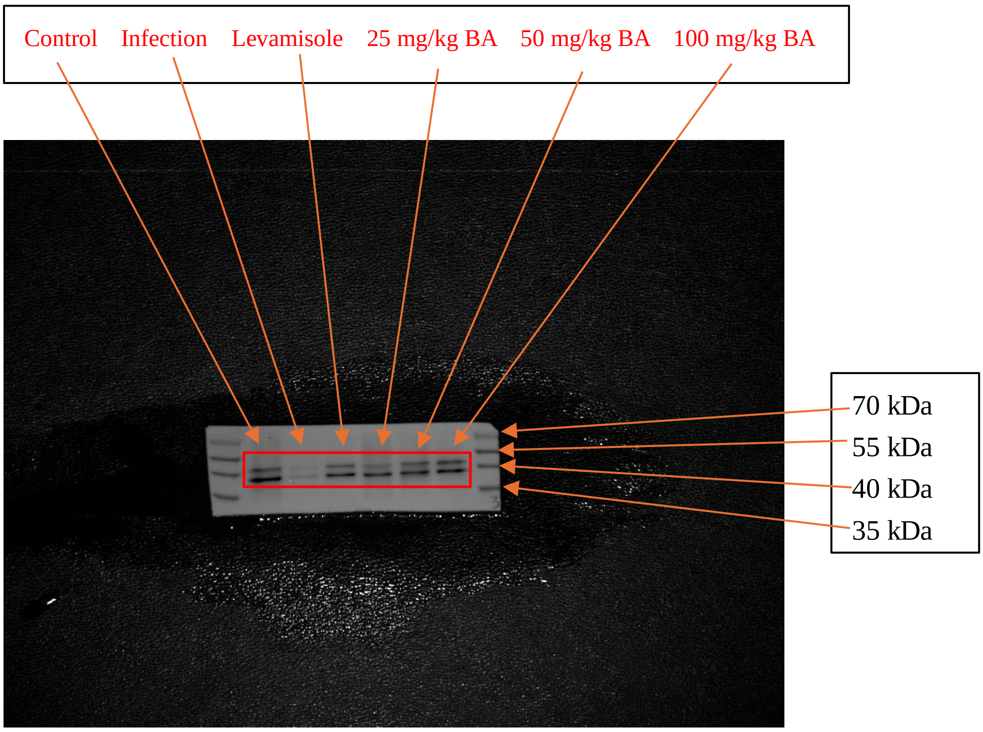

Fig S5

Fig S5. B:

GAPDH (repeat 1)

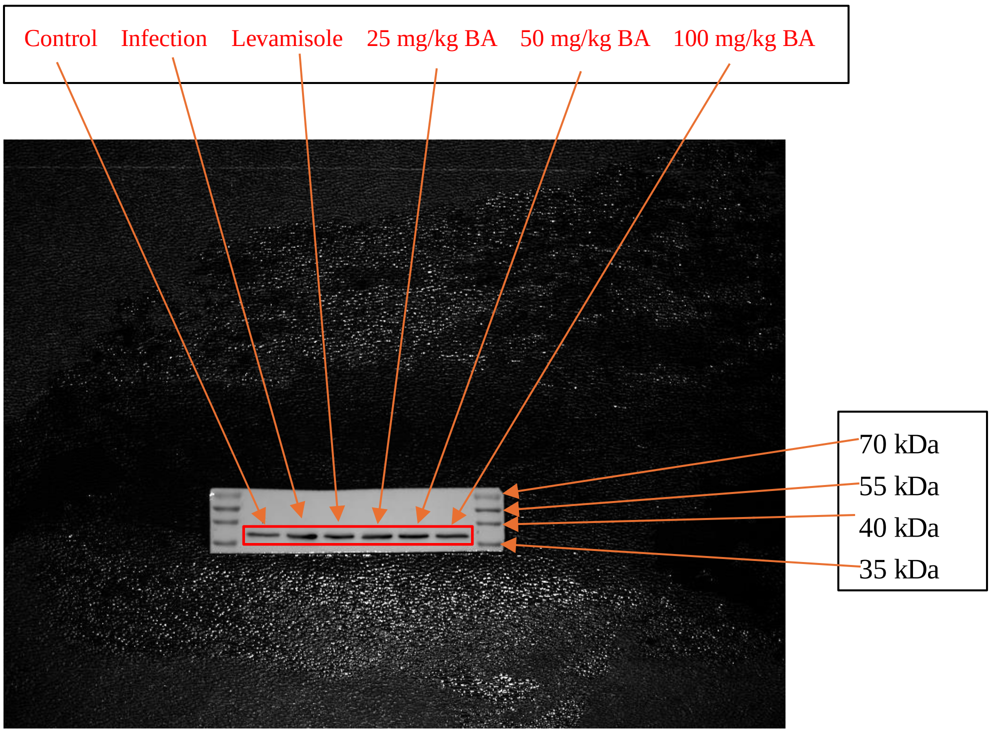

Fig S5

Fig S5. B:

GAPDH (repeat 2)

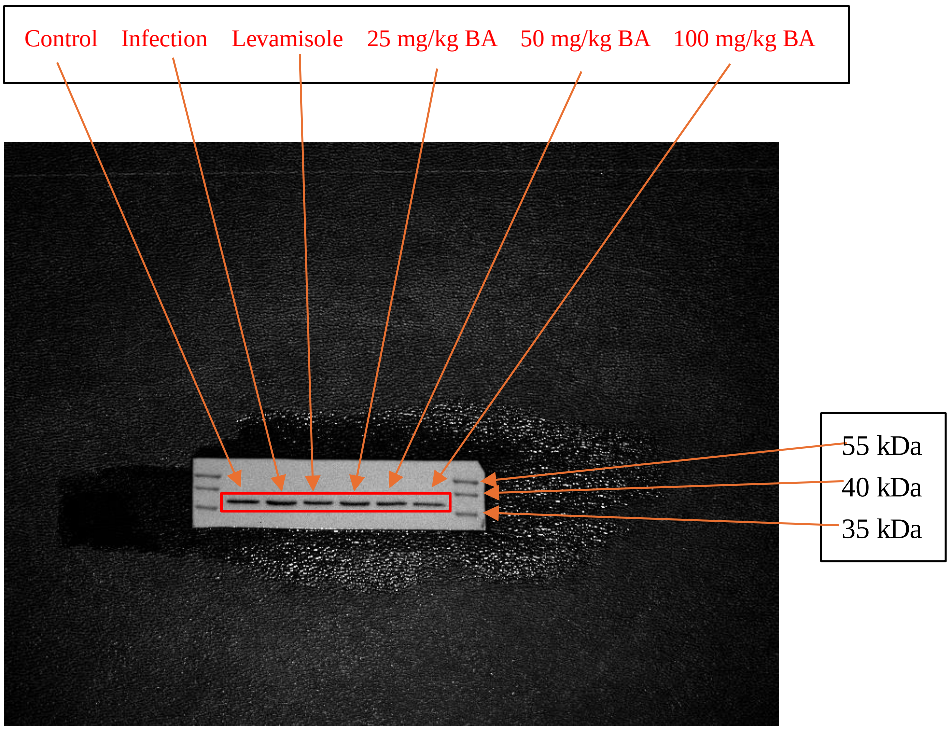

Fig S5

Fig S5. B:

GAPDH (repeat 3)

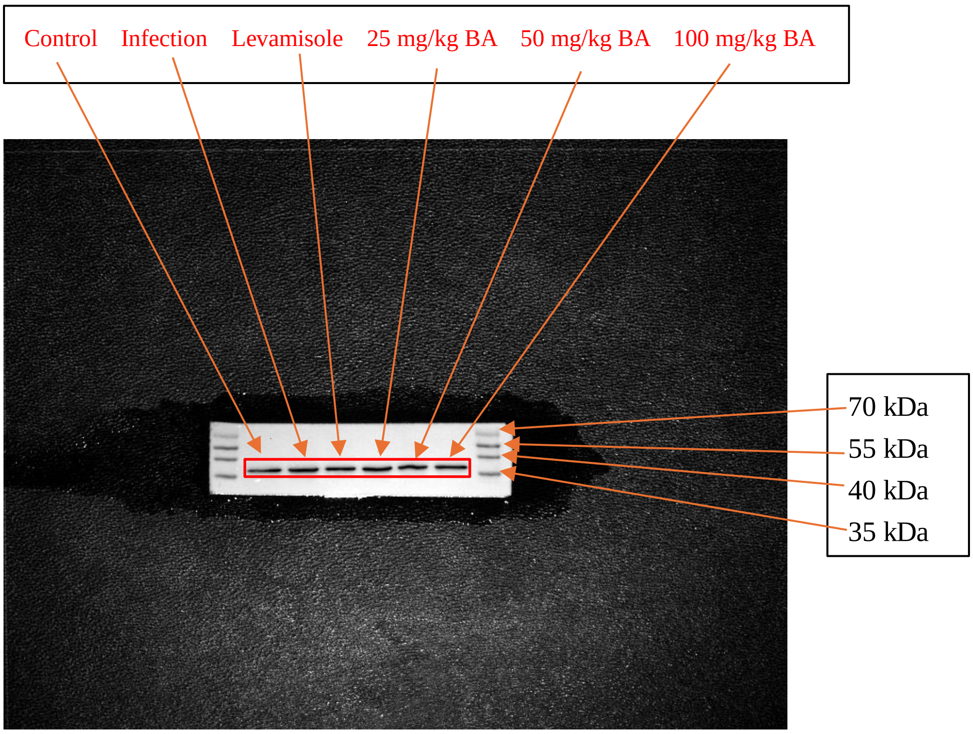

Fig S5

Fig S5. B:

LC3B (repeat 1)

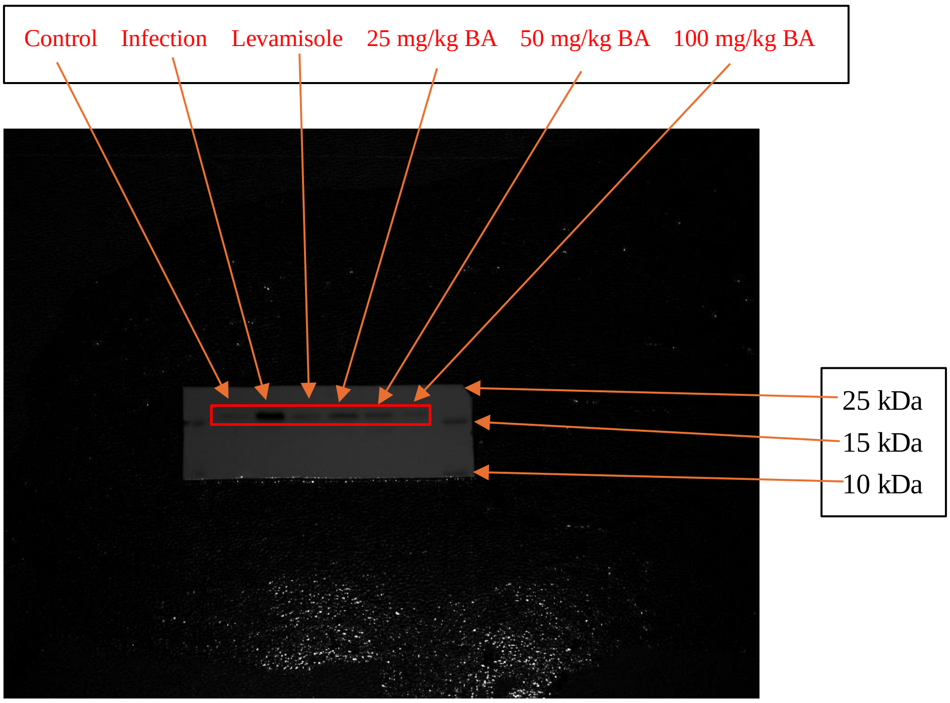

Fig S5

Fig S5. B:

LC3B (repeat 2)

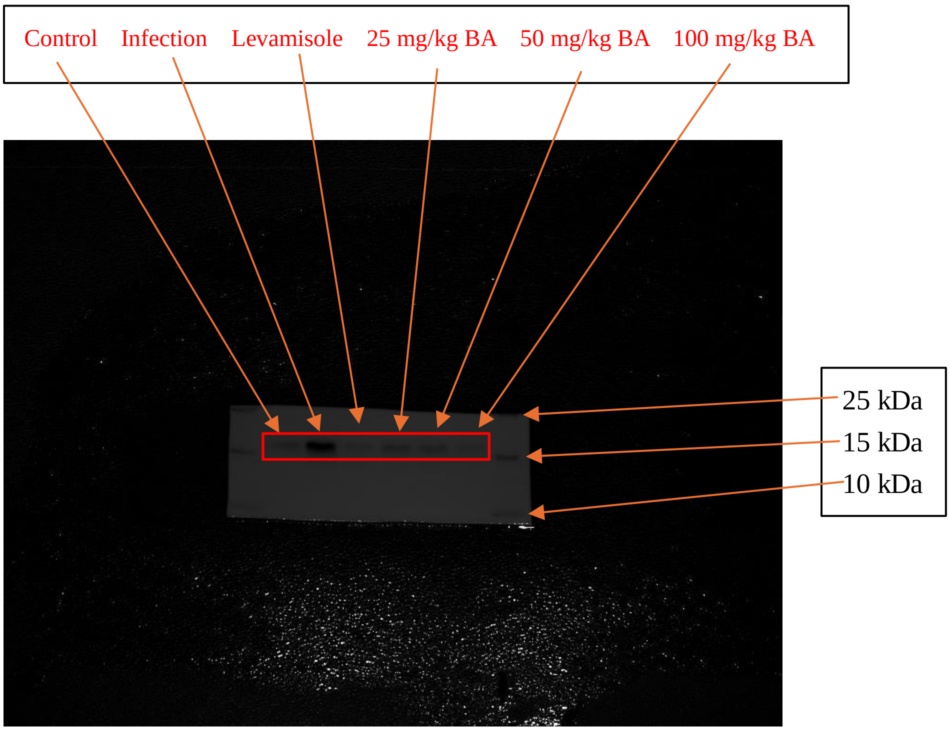

Fig S5

Fig S5. B:

LC3B (repeat 3)

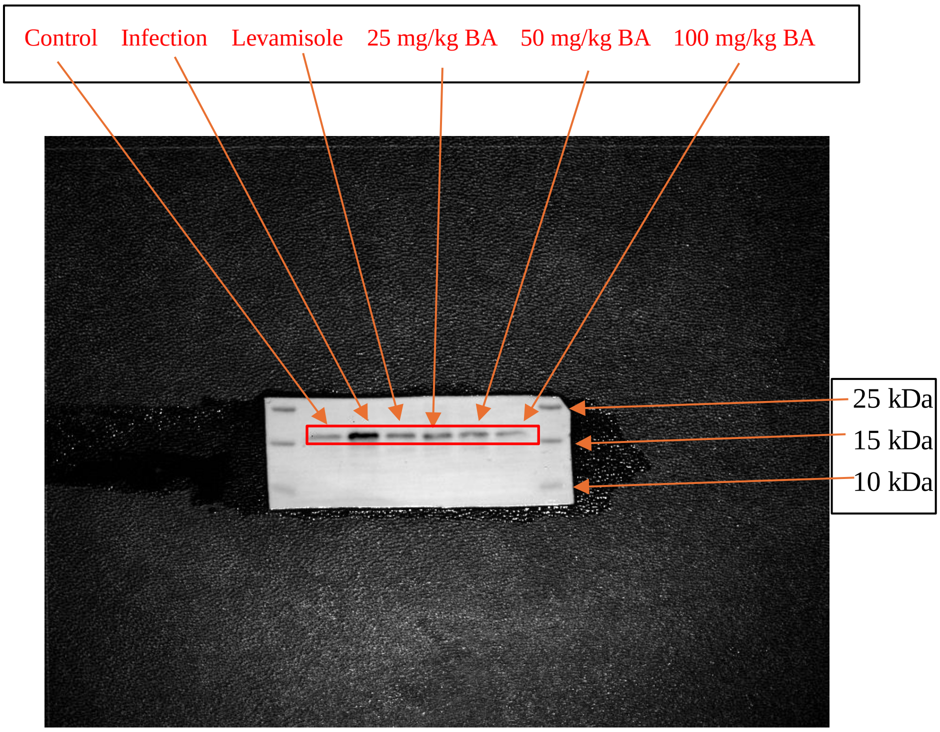

Fig S5

Fig S5. E:

GAPDH (repeat 1)

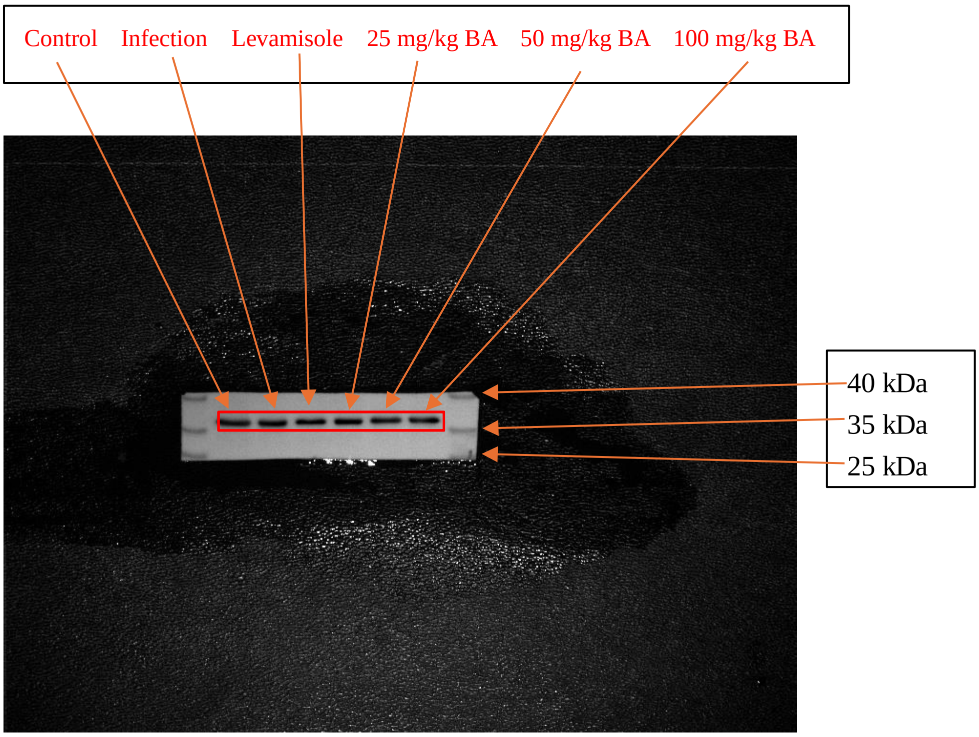

Fig S5

Fig S5. E:

GAPDH (repeat 2)

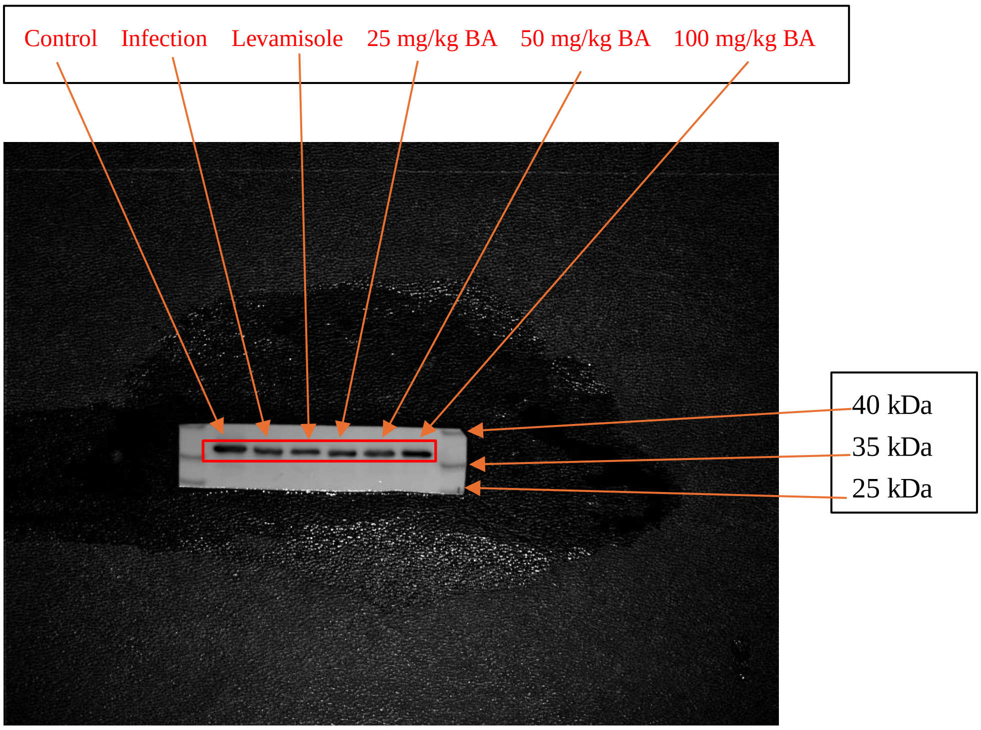

Fig S5

Fig S5. E:

GAPDH (repeat 3)

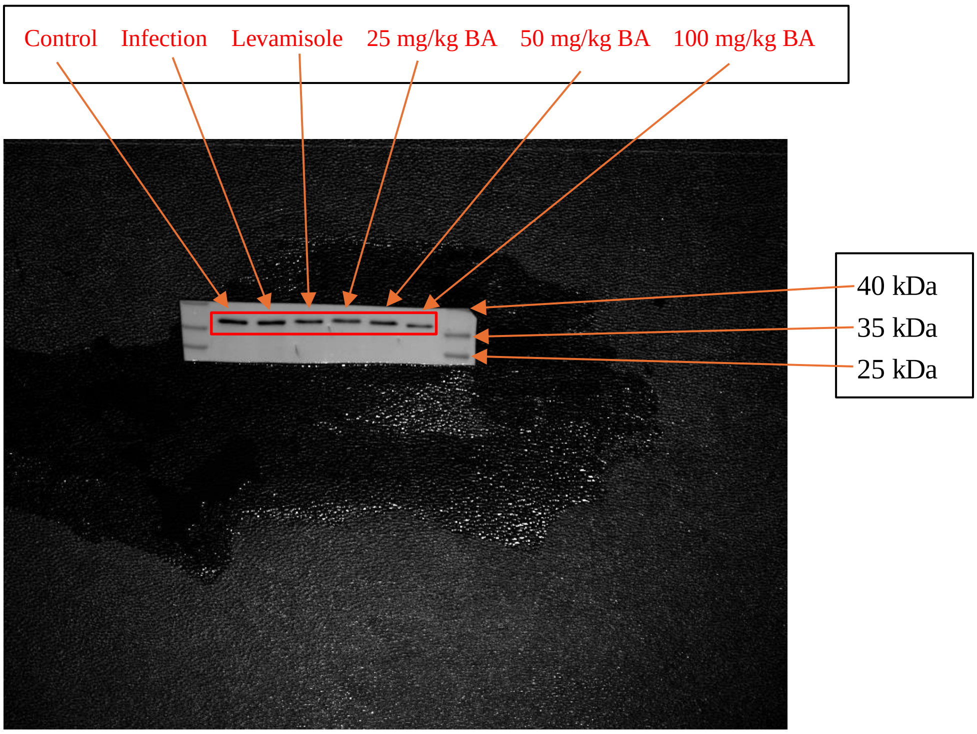

Fig S5

Fig S5. E:

P62 (repeat 1)

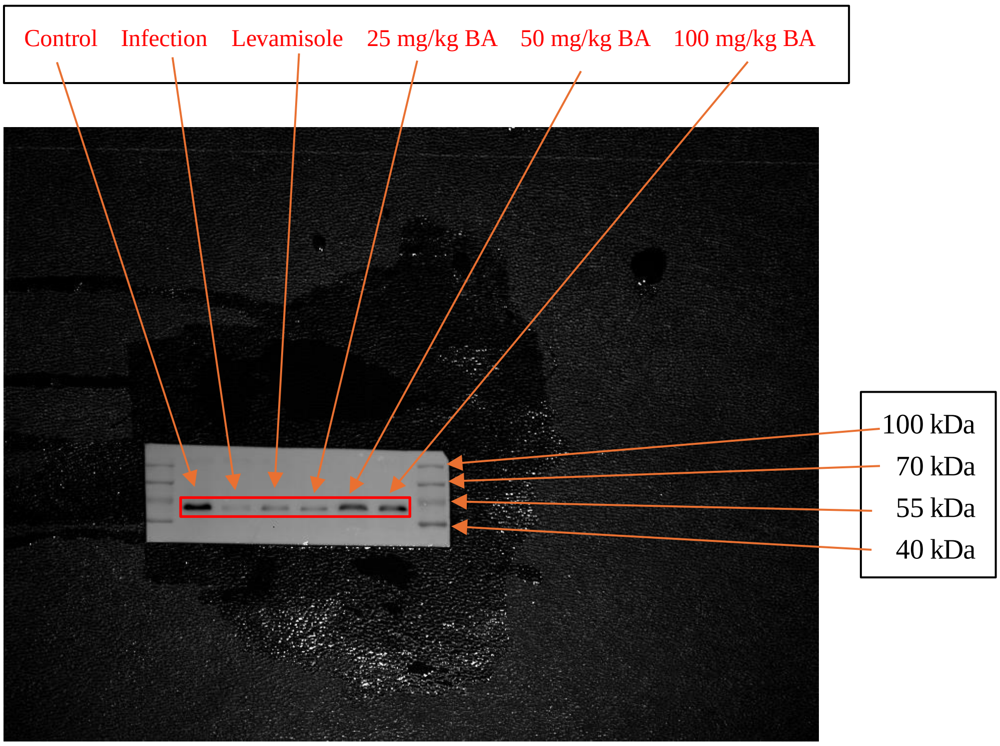

Fig S5

Fig S5. E:

P62 (repeat 2)

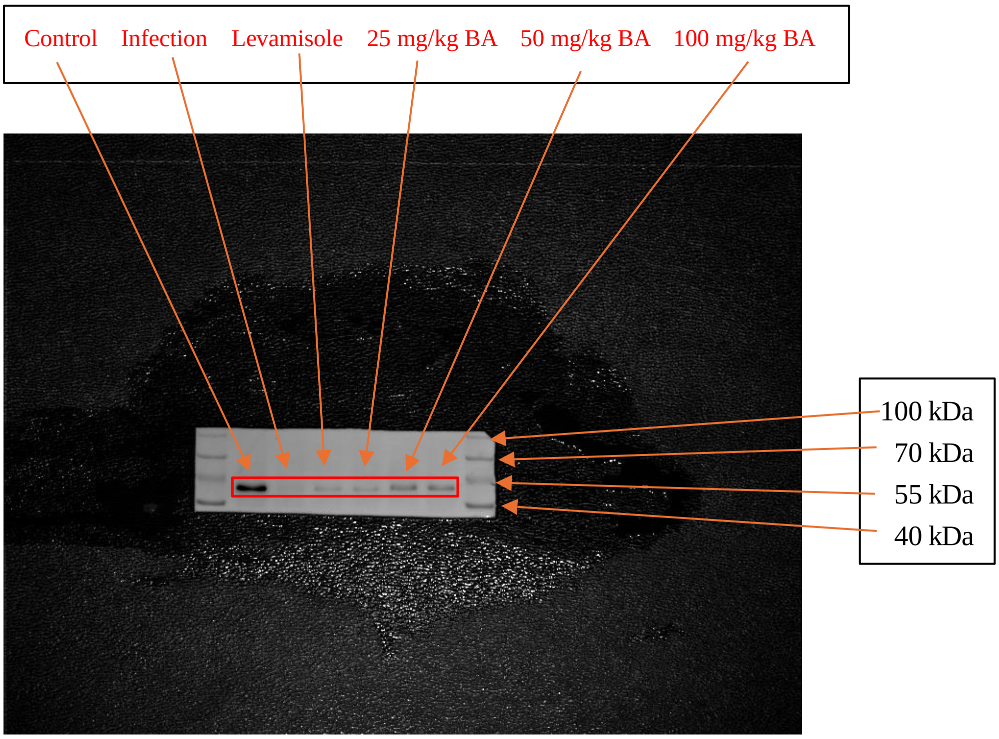

Fig S5

Fig S5. E:

P62 (repeat 3)

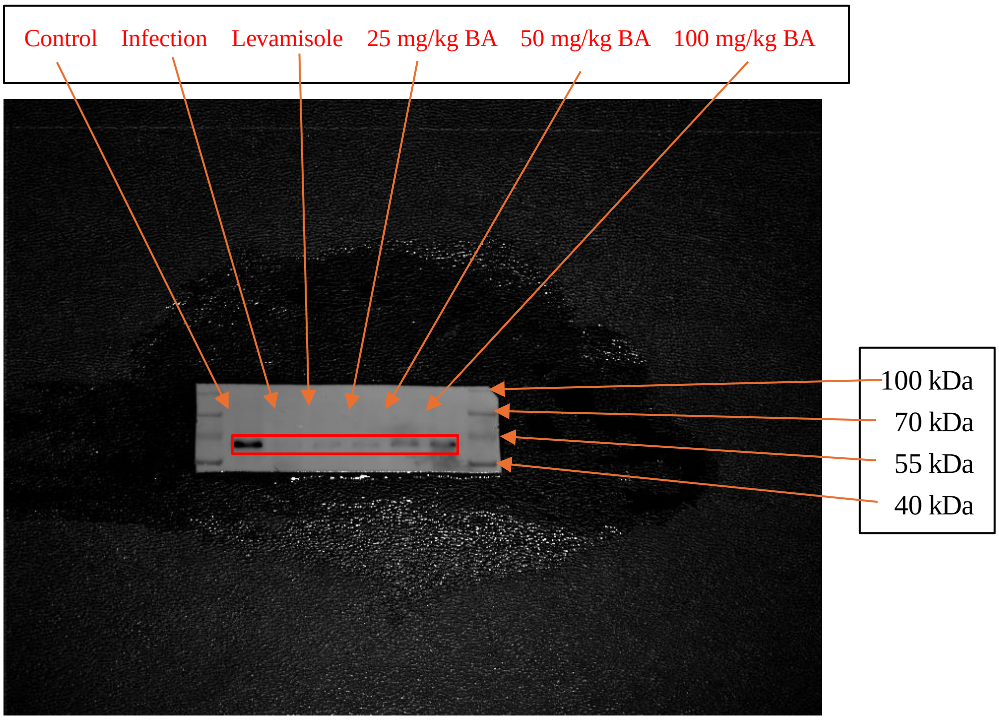

Fig S5

Fig S5. H:

GAPDH (repeat 1)

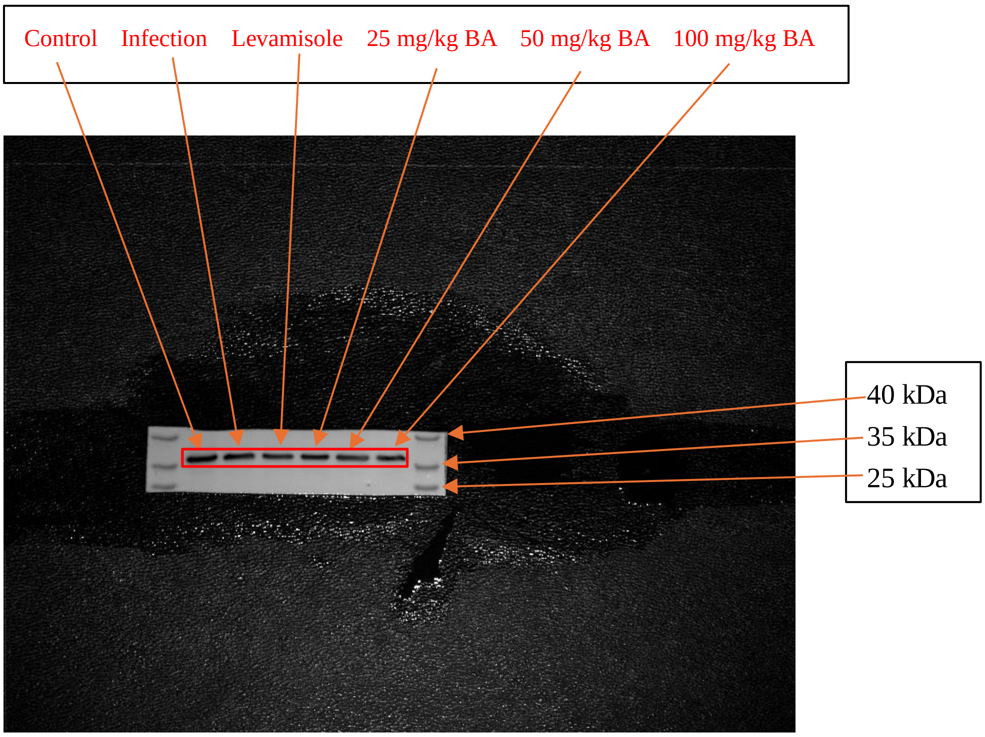

Fig S5

Fig S5. H:

GAPDH (repeat 2)

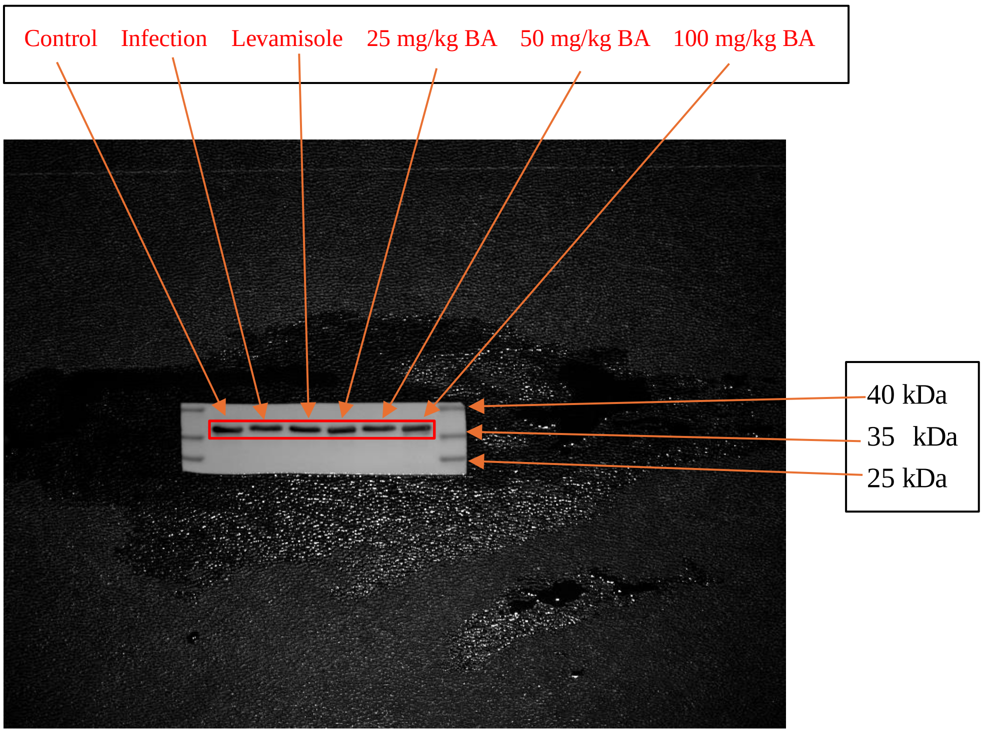

Fig S5

Fig S5. H:

Beclin1 (repeat 1)

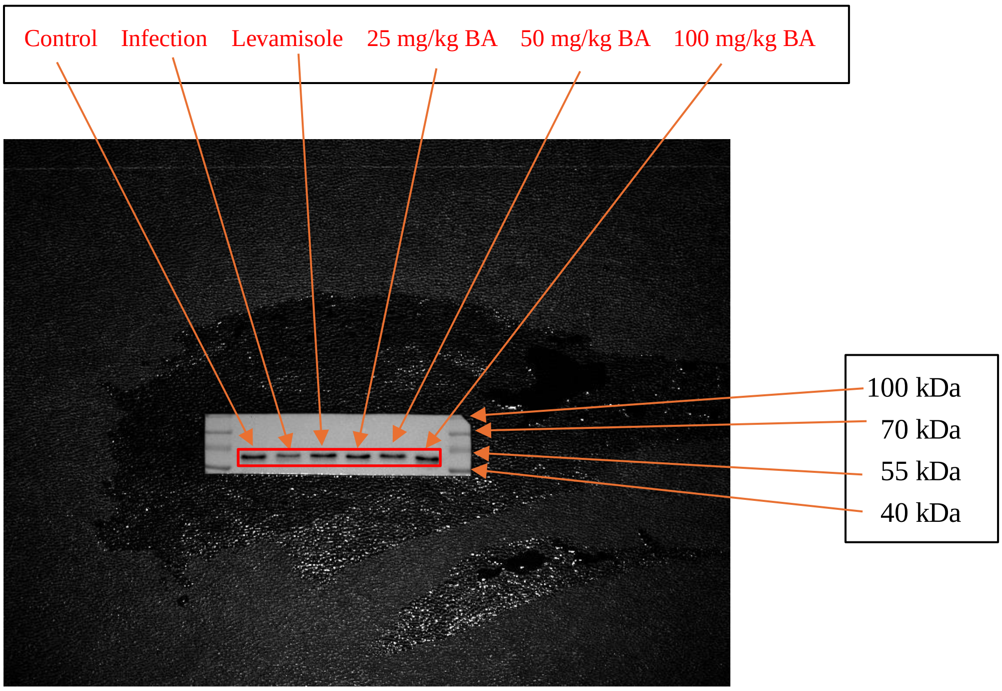

Fig S5

Fig S5. H:

Beclin1 (repeat 2)

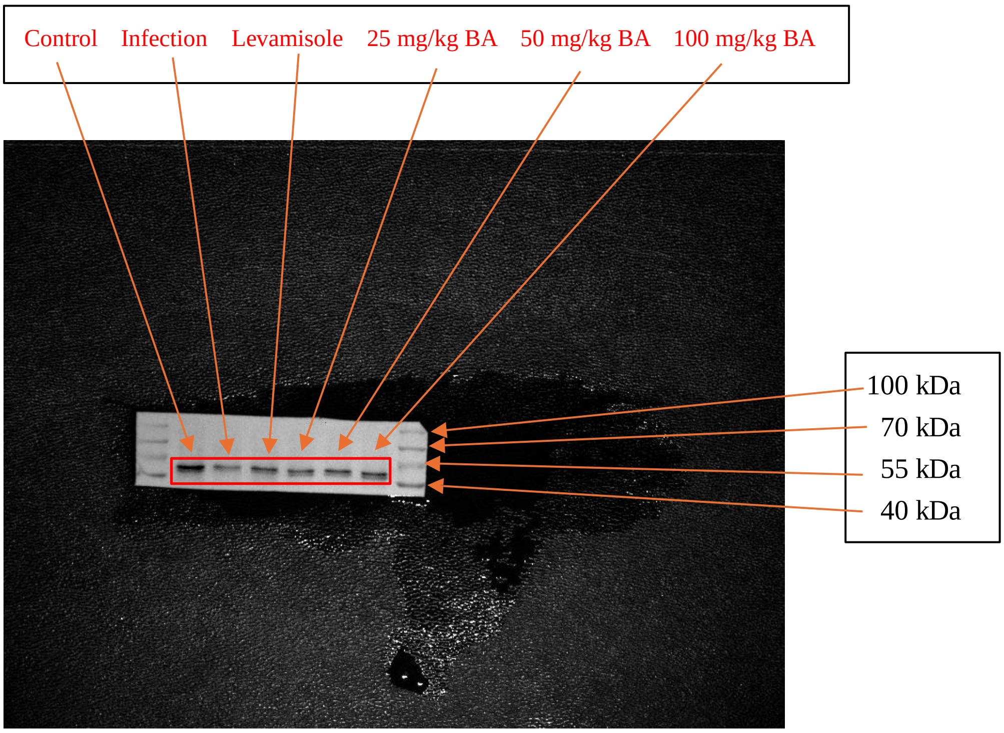

Fig S5

Fig S5. H:

Beclin1 (repeat 3)

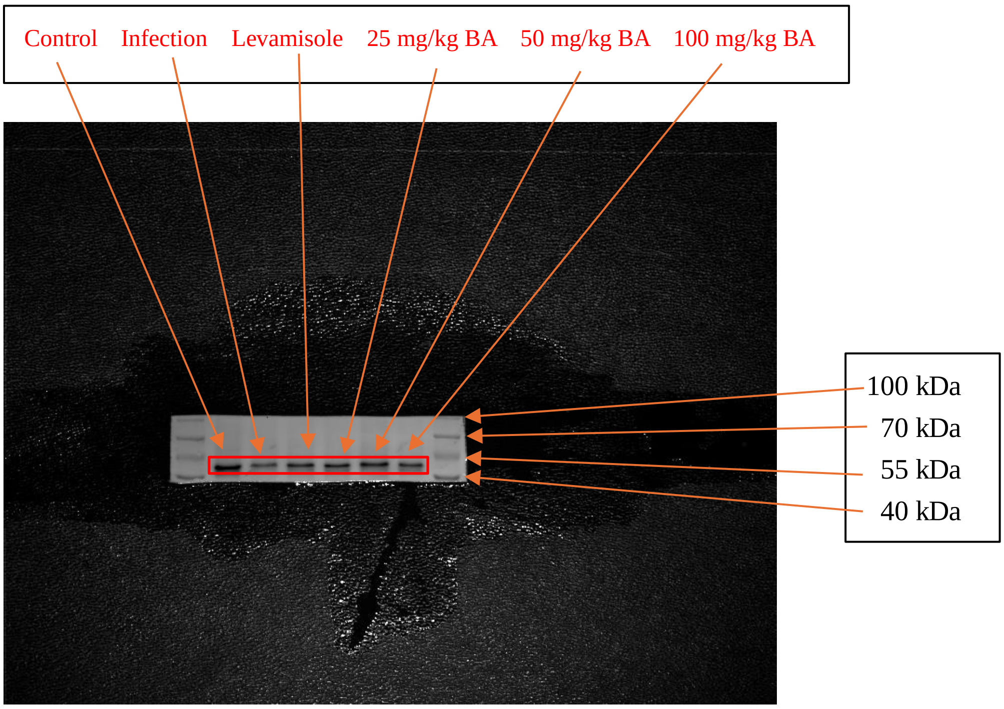

Supplement: Supplementary file 1 [file biomolecules-15-00640-s001.zip › biomolecules-3520128-supplementary.pdf]
